# Circ_0060927 regulates miR-331-3p/ERK/MAPK pathway reaction in non-small cell lung cancer through METTL14-driven methylation

**DOI:** 10.3389/fonc.2025.1609215

**Published:** 2025-11-13

**Authors:** Yang Liao, Ji Song, Chun Luo, Jin Xiang, Sheng Cheng

**Affiliations:** 1 Department of Pulmonary and Critical Care Medicine, University-Town Hospital of Chongqing Medical University, Chongqing, China; 2 Department of Otorhinolaryngology, University-Town Hospital of Chongqing Medical University, Chongqing, China

**Keywords:** m6A methylation, NSCLC, miR-331-3p, circ_0060927, ERK/MAPK signaling axis

## Abstract

**Background:**

Non-coding RNA is one of the most paramount genetic regulators. circRNAs (Circular RNAs) are a potential modulator of various tumors, especially in non-small cell lung cancer (NSCLC). This study focused on elucidating the underlying influence of circ_0060927 on NSCLC progression and relevant regulatory mechanisms.

**Methods:**

Manipulating circ_0060927, MAP2K7, and METTL14 expression in NSCLC cells by lentiviral vectors. The expression of MAP2K7, ERK, p-ERK, p38MAPK, and p-p38MAPK was measured by RT-qPCR and Western Blot experiments. RIP-RT-qPCR was performed to quantify the m6A methylation status of circ_0060927. Cell Counting Kit-8, cell apoptosis assay, wound healing assay, and transwell assay were employed to identify the discrepancy of tumor cells’ malignant phenotypes, including cell proliferation, cell motility, and apoptosis.

**Results:**

L78 and A549 cells showed high expression of circ_0060927 and its 6A methylation level compared to normal lung epithelial cells. Circ_0060927 could promote tumor cells’ proliferation and motility, and reduce cell apoptosis. These effects depended on the m6A methylation derived from METTL14. Besides, circ_0060927 combined miR-331-3p specifically in NSCLC cells to repress its function; simultaneously, miR-331-3p impeded NSCLC cells through suppressing the ERK/MAPK pathway activation by interfering with the expression of MAP2K7 protein.

**Conclusions:**

Circ_0060927 positively regulates NSCLC cells’ malignant biological behaviors via targeted binding to miR-331-3p. MiR-331-3p could be recognized as a promising inhibitor for the ERK/MAPK signal pathway in NSCLC cells since it has an interfering effect on MAP2K7 protein. These results cast a meaningful insight into the shadow regarding molecular mechanisms underlying NSCLC and the potential therapeutic perspective of circRNA methylation.

## Introduction

Non-small cell lung cancer (NSCLC) is recognized as the most prevalent type of lung cancer among patients diagnosed with this disease, accounting for a staggering 80% to 85% of all lung cancer cases. Over the past few decades, both the incidence and mortality rates associated with NSCLC have shown a concerning upward trend on a global scale, solidifying its status as one of the leading causes of cancer-related deaths worldwide (1). The complexity inherent in NSCLC is evident in its wide array of molecular characteristics and clinical manifestations, which together pose numerous challenges for effective treatment strategies. However, with the rapid advancements in the fields of molecular biology and immunology, research focused on NSCLC has expanded significantly, particularly with the emergence of groundbreaking developments in targeted molecular therapy and immunotherapy. These innovative treatment modalities are providing substantial clinical benefits for patients, marking a shift in the therapeutic landscape (2). Traditional chemotherapy, once the cornerstone of cancer treatment, is gradually being supplanted by more precise and tailored approaches, including targeted therapy and immunotherapy. These new strategies have been shown to significantly enhance patients’ survival rates and improve their overall quality of life by specifically targeting unique molecular markers associated with the disease (3, 4). Despite the ongoing evolution of treatment methodologies, NSCLC continues to present as a highly heterogeneous disease, and a considerable number of patients still grapple with the challenges of drug resistance and disease progression, even after undergoing treatment (5).

Circular RNAs (CircRNAs) are a distinctive type of regulatory non-coding RNA formed through a specialized splicing mechanism that sets them apart from linear RNA forms (6, 7). Instead of being mere byproducts of gene expression, circRNAs play a variety of essential biological roles that are vital for numerous cellular processes (8). They are known to influence developmental pathways, regulate gene expression, and actively engage in the complex processes associated with tumorigenesis (9, 10). Recent research has highlighted the significant role circRNAs may have in the development of lung cancer, also providing a new perspective on the molecular mechanisms at the transcriptional layer (11, 12). For instance, circRNA (circ_100395) is closely involved in lung cancer pathogenesis by regulating key cellular functions such as cell growth, migration, and invasion ability, all of which are critical for cancer progression (13, 14). Circ_0000677 was found to promote the proliferation of NSCLC cells by regulating the miR-106b/CCND1 axis (15). Circ_0008594 has been confirmed to promote the proliferation and metastasis of NSCLC cells by regulating the miR-760-mediated PI3K/AKT and MEK/ERK signaling pathways (16). The expression level of circRNA is closely related to clinical features such as tumor size, degree of differentiation, lymph node metastasis, and TNM staging. The high expression of hsa_circ_0046264 is significantly associated with larger tumor size and higher TNM staging (17), suggesting that it may serve as a prognostic marker for lung cancer progression. However, it is crucial to recognize that the functions of most circRNAs in various biological processes remain largely ambiguous, presenting a huge opportunity that could lead to important discoveries in cancer research.

Compelling evidence suggests that circ_0060927 is significantly overexpressed in colorectal cancer, uterine leiomyomas, and oral squamous cell carcinoma (18–20). This specific circular RNA, circ_0060927, undertakes a vital role in modulating the activity of miR-331-3p, a microRNA that targets and suppresses the expression of MAP2K7 (mitogen-Activated Protein Kinase 7), an essential element of the MAPK (mitogen-Activated Protein Kinase) signaling pathway (21, 22). The regulation occurs through RNA interference, resulting in a decrease in the MAPK signaling pathway activation (22). N6-methyladenosine (m6A) is a common epigenetic modification form in non-coding RNA, usually derived by the METTL3/METTL14 methyltransferase complex catalysis (23, 24). METTL14, involved in the dynamic reversible process of m6A modification, is a core component of the m6A methylation transferase complex (25). The dynamic characteristic and flexibility of m6A modification make it an important mechanism for regulating gene expression. METTL14 not only plays a role by directly influencing the fate of mRNA but may also regulate gene expression through interactions with other transcription factors and modifying enzymes (26). As exploration on m6A modification deepens, more and more evidence suggests that METTL14 plays an important role in various cancer types (27–29). This provides us with a new perspective on understanding the role of m6A modification in tumorigenesis. Therefore, in-depth research on the role of METTL14 in m6A modification and its biological significance is of great clinical importance for elucidating its functions in health and disease.

Based on the limited background of m6A methylation of circRNA in NSCLC, we undertook a comprehensive investigation into the latent relationship between circ_0060927 and its regulation through m6A methylation, specifically mediated by the methyltransferase METTL14, and how this interplay influences the progression of NSCLC. Furthermore, we directed our attention towards identifying the direct effectors induced by circ_0060927, which play a crucial role in modulating the biological behaviors of tumor cells. In summary, this research unveiled a complex and integral regulatory network that is intricately associated with the methylated modification of circRNA, highlighting the relevant molecular signaling pathways and their significant biological effects on the progression and development of NSCLC.

## Methods

### Cell culture

Human normal lung epithelial cell (BEAS-2B) was bought from Beyotime Biotechnology (Shanghai, China, C6106). Human non-small cell lung cancer cells (L78 and A549) were acquired from Otwo Biotechnology company (Shenzhen, China, HTX-2985) and Pricella (Wuhan, China, CL-0016), respectively. All cell lines were validated by short tandem repeats (STR) assay and identified as free of mycoplasma. BEAS-28, L78, and A549 cells were maintained with RPMI-1640 medium (Gibco, China, 11875093), high-glucose DMEM (Gibco, China, 11995065), and Ham’s F-12K medium (Pricella, China, PM150910), respectively. All mediums were added with 10% fetal bovine serum (FBS) (Excell Bio, China, FSD500) and 1% penicillin/streptomycin (Beyotime, China, C0222). All cultivation processes were carried out in a 37°C and 5% CO_2_ incubator.

### RNA immunoprecipitation

RNA immunoprecipitation (RIP) assays were conducted across three human cell lines (BEAS-2B, L78, and A549 cells) using the BersinBio RNA Immunoprecipitation Kit (Bes5150, BersinBio, China). Briefly, cellular lysates were prepared using ice-cold RNA lysis buffer supplemented with RNase/protease inhibitors (1:100 v/v). Magnetic Protein A/G beads pre-coupled with either anti-METTL3 m6A-specific monoclonal antibody or species-matched IgG isotype control were incubated with lysates for 4 hr at 4°C under gentle rotation. Post-immunoprecipitation complexes were treated with Proteinase K (20 mg/mL) at 55°C for 1 h to digest protein-RNA crosslinks. Purified RNA was subsequently quantified via Nanodrop 2000 (Thermo Scientific) and analyzed by TaqMan-based RT-qPCR.

### RT-qPCR

Total RNA of BEAS-2B, L78, and A549 was acquired by the Cell/Tissue Total RNA Isolation Kit V2 (Vazyme, China, RC112), and miRNA was separated by the MiPure Cell/Tissue miRNA Kit (RC201). All procedures accorded with the manufacturer’s description rigorously. RNA concentration of each sample was confirmed with the Nano 6000 (Jiapeng Tech, Shanghai, China). Complementary DNA (cDNA) was synthesized utilizing the cDNA Synthesis Kit (China, Vazyme, R312) and miRNA 1st Strand cDNA Synthesis Kit (Vazyme, China, MR101). RT-qPCR procedure was conducted using the Taq Pro Universal SYBR qPCR Master Mix reagent (Vazyme, Q712, China) and miRNA Universal SYBR qPCR Master Mix (MQ101, Vazyme, China). Reactions were performed, and data were analyzed via CFX96 Touch Real-Time PCR machine (1855195, Bio-Red, USA). All target gene expression levels were normalized by GAPDH expression and quantified by the ΔCT method, presenting results as fold changes in expression. The sequences information of primer is shown as: U6-forward: CAAATTCGTGAAGCGTTCCA, U6-reverse: AGTGCAGGGTCCGAGGTATT; circ_0060927-forward: GAACTCCCCATCGCGTTTTG, circ_0060927-reverse: TGTCCAGCTTCATCACTTCCC; miR-331-3p-forward: CGGCCCCTGGGCCTATC, miR-331-3p-reverse: AGTGCAGGGTCCGAGGTATT; GAPDH-forward: GAAAGCCTGCCGGTGACTAA, GAPDH-reverse: GCATCACCCGGAGGAGAAAT.

### Vector construction and transfection

Overexpression and knockdown lentiviral vectors of circ_0060927, MAP2K7, and METTL14 and a relative control vector were constructed by GenePharma (Suzhou, China). Briefly, the DNA segment of circ_0060927, MAP2K7, and METTL14 was cloned on the pLV-Puro vector, and the relative shRNA coding sequences were cloned into the pLKO.1 with the shRNA construct vector. HEK293T cells (IM-H222, Immocell, China) were used to package lentiviral particles, then L78 and A549 cells were infected with lentiviral particles in order to stably overexpress or knockdown circ_0060927, MAP2K7, and METTL14. Commercial miR-331-3p mimic and miR-331-3p inhibitor were bought from Ribo Biotec (Guangzhou, China). Finally, Lipofectamine 3000 reagent (L3000015, ThermoFisher, USA) was used to execute the cell transfection procedure.

### Wound healing assay

Cellular monolayers were established by seeding 1×10^6^ logarithmically growing cells per well in six replicate culture plates. Following attainment of ≥95% confluency, standardized mechanical wounding was performed using sterile 200 μL pipette tips aligned perpendicularly to pre-etched grid coordinates on the culture vessel base. Post-wounding protocol involved sequential PBS rinses (2×5 mL) for debris removal, followed by immediate brightfield imaging (0 h timepoint) using an inverted phase-contrast microscope equipped with a calibrated ocular micrometer. Subsequent to initial documentation, the wounded monolayers underwent 24-hour incubation under normal culture environment (37°C, 5% CO_2_) before terminal imaging under identical optical parameters for comparative wound closure analysis.

### Transwell assay

The extracellular matrix solution was prepared by reconstituting Matrigel™ (Corning Inc., NY, USA, #364230) in serum-free culture medium at 1:4 (v/v) dilution. Following reconstitution, 50 μL aliquots were applied uniformly across Transwell^®^ membrane inserts (8 μm pores, Corning #CLS3422) and polymerized under standard culture conditions (37°C, 6 h, 5% CO_2_). For migration assays, 100 μL cell suspensions (2×10^5^ cells/mL in serum-free medium) were transferred to the coated upper chambers, while 600 μL complete medium containing 10% FBS (v/v) was introduced into the lower compartment to establish chemotactic gradients. Post 24-h incubation, non-migratory cells were removed through mechanical detachment followed by PBS-immersion cleaning protocols. Fixed specimens were processed through sequential treatments with 4% paraformaldehyde (Beyotime Biotech, #P0090, 30 min RT) and 0.1% crystal violet solution (Sigma-Aldrich, #V5265; 20 min RT). Quantitative analysis involved (i) systematic microscopic enumeration of transmembranous cells across five non-overlapping 200× fields per insert using differential interference contrast optics, and (ii) subsequent normalization of migration indices against control groups via digital image analysis (ImageJ v1.53a, NIH).

### CCK-8 experiment

Cells in logarithmic growth phase were plated in a 96-well plate. After adhering to the plate, cells were cultured for 24 h continuously. Every well was infused with 100 µL CCK-8 working solution, then incubated at room temperature for 2 h. CCK-8 assay kit was purchased from Bioss Biotechnology (Beijing, China, BA00208). Last, microplate reader (Multiskan FC, 357-714018) was used to read every well’s absorbance at 450 nm.

### Flow cytometric analysis

Apoptotic characterization was conducted using Annexin V-FITC/PI dual staining (Yeasen Biotechnology, Shanghai, Cat#40302ES60) following standardized procedures. Logarithmic-phase cell suspensions were harvested via trypsin-EDTA solution (0.25%, Gibco™) and washed twice with ice-cold PBS. Following density adjustment to 1×10^6^ cells/mL in Annexin V binding buffer (1×, pH 7.4), samples underwent sequential fluorescent labeling: 5 μL Annexin V-FITC (Ex/Em 488/519 nm) with 10-min dark incubation (22°C), followed by propidium iodide counterstaining (5 μg/mL, Ex/Em 535/617 nm) according to manufacturer’s specifications (Yeasen Biotechnology, rev.2023). Quantitative analysis was performed within 1 h post-staining using a Beckman Coulter CytoFLEX S4 cytometer equipped with CytExpert 2.4 software, employing standardized gating strategies for viable (Annexin V^-^/PI^-^), early apoptotic (Annexin V^+^/PI^-^), and late apoptotic (Annexin V^+^/PI^+^) populations.

### Western blotting

Western immunoblotting was executed for proteomic analysis under standardized conditions. Cell lysates were prepared in RIPA buffer (Beyotime Biotechnology, P0013B) supplemented with 1 mM PMSF protease inhibitor (ST506, Beyotime) through mechanical homogenization (30 min, 4 °C). Protein quantification was performed via bicinchoninic acid assay (BCA Kit, WB6501, NCM Biotech) with bovine serum albumin calibration curves (0.1-2.0 mg/mL range). Equal protein aliquots (30 μg) underwent electrophoretic separation through 12% SDS-PAGE (Mini-PROTEAN^®^ Tetra System, Bio-Rad) followed by semi-dry transfer to PVDF membranes (0.45 μm, Millipore). Post-transfer blocking employed 5% non-fat milk/TBST (1 h, 25 °C) prior to 16-h incubation (4°C) with primary antibodies (diluted 1:1000 in TBST) targeting specific epitopes. Membranes received three TBST washes (10 min/wash) before 2-h exposure to HRP-conjugated secondary antibodies (1:5000, 25°C). Chemiluminescent signals were detected using an ECL Advanced™ substrate (P2100, NCM Biotech) with digital acquisition via ChemiDoc™ MP imaging system (Bio-Rad). Band densitometry analysis was conducted using ImageJ (v1.53a, NIH) with GAPDH serving as the endogenous loading control for data normalization. The detail and concentration of antibodies used in Western blotting assay are listed as: MAP2K7 (ab52618, Abcam, UK); ERK (ab32537, Abcam, US); p-ERK (ab229912, Abcam, UK); p38MAPK (#9212, CST, UK); p-p38MAPK (ab178867, Abcam, UK); GAPDH (ab8245, Abcam, UK); Goat Anti-Rabbit IgG H&L/HRP (bs-0295G-HRP, Bioss, China).

### Dual-luciferase reporter assay

Bioinformatic prediction of RNA interactions was systematically performed using the Circular RNA Interactome database (https://circinteractome.nia.nih.gov/) for circ_0060927:miR-331-3p pairing and Targetscan (https://www.targetscan.org/vert_80/) algorithm for miR-331-3p::MAP2K7 targeting. Chimeric luciferase reporter constructs were commercially synthesized (Shanghai Sangon Biotech, SYN-2305P) containing either conserved (WT) or mutated (MUT) miR-331-3p response elements within both circ_0060927 and MAP2K7 3'-UTR sequences, directionally cloned into the pmirGLO dual-luciferase vector (Promega, E1330) via XhoI/NotI restriction sites. For functional validation, L78 and A549 cell lines were transiently transfected with 500 ng plasmid DNA using Lipofectamine™ 3000 transfection reagent (Invitrogen, L3000015) under serum-free Opti-MEM conditions. Luciferase activity was systematically quantified 48 h post-transfection and normalized to Renilla luciferase internal controls.

### Statistics

All quantitative data were analyzed using GraphPad Prism (v9.5.1, GraphPad Software Inc.) for statistical computations and visualization, with ImageJ (v1.53t, NIH) employed for densitometric quantification of immunoblots. Continuous variables are expressed as mean ± standard deviation (SD) unless otherwise specified. Intergroup comparisons were performed as follows: Two-group comparisons: Unpaired Student’s t-test with Welch’s correction for unequal variances (assessed via F-test). Multi-group comparisons: One-way ANOVA followed by Tukey’s *post hoc* test for normally distributed data. Statistical significance thresholds were defined as: *p < 0.05, **p < 0.01, ***p < 0.001.

## Results

### Circ_0060927 is up-regulated in NSCLC and positively associated with cancer cells’ malignant phenotypes

**Figure 1:**
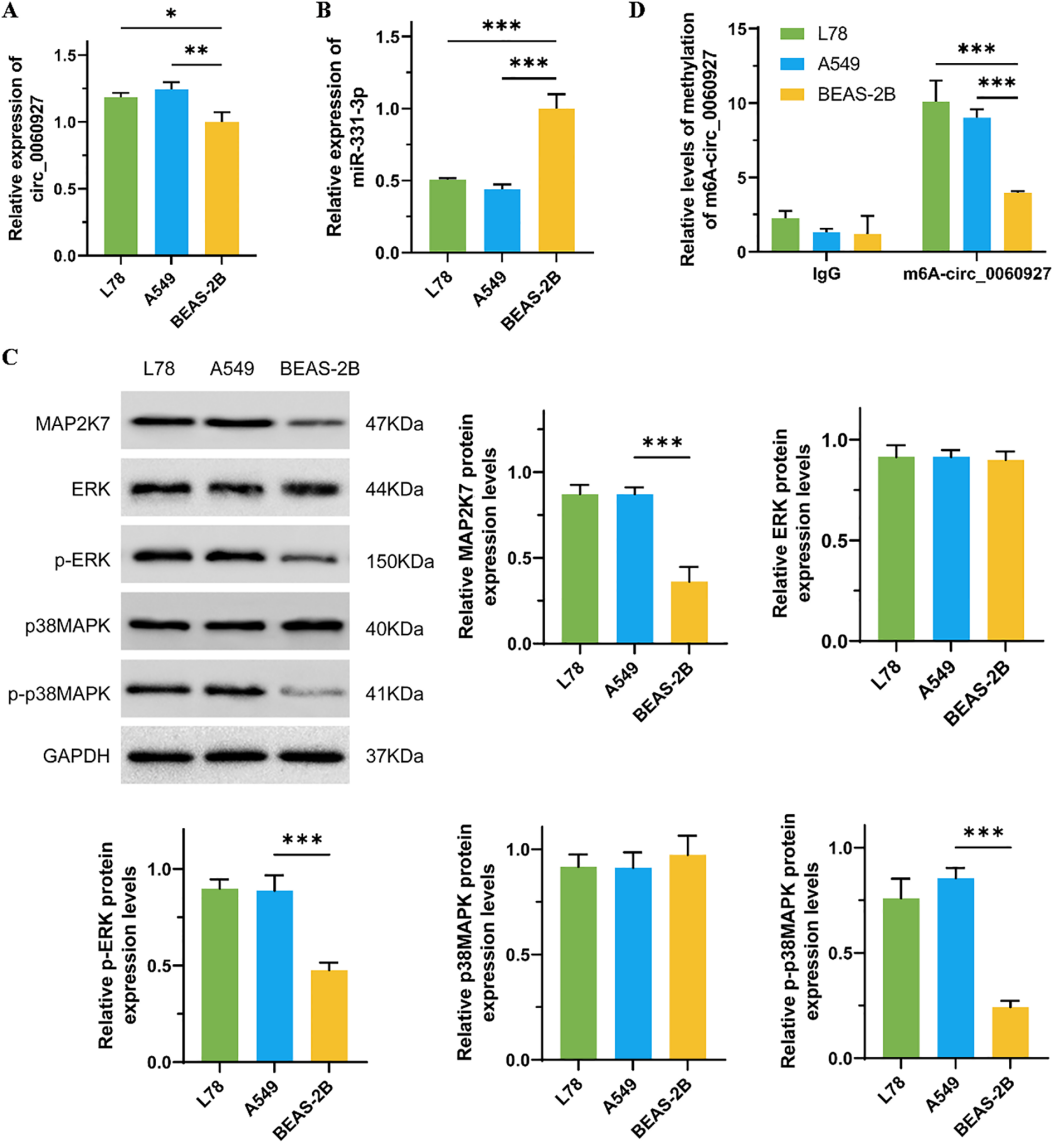
The relevant molecules’ expression in NSCLC and normal lung epithelial cells. **(A)** The comparison of the expression of circ_0060927 in BEAS-2B, L78, and A549 cells. **(B)** The differential expression of miR-331-3p in BEAS-2B, L78, and A549 cells. **(C)** The protein level of ERK/MAPK pathway markers in BEAS-2B, L78, and A549 cells. **(D)** The m6A modification level of circ_0060927 in BEAS-2B, L78, and A549 cells. n=3, *(p < 0.05), **(p < 0.01), ***(p < 0.001).

We first used qRT-PCR assay to measure the expression levels of circ_0060927 and miR-331-3p in NSCLC cell lines (L78 and A549) and normal lung epithelial cells (BEAS-2B). The results showed that circ_0060927 had significantly increasing expression in L78 and A549 cells compared to BEAS-2B cells (**Figure 1A**), and miR-331-3p expression in BEAS-2B cells was significantly higher than that in L78 and A549 cells (**Figure 1B**). Although circ_0060927 expression in limited lung cancer samples is not pronounced higher in GEO datasets, the expression of its host gene CYP24A1 in lung cancer tissues is much higher both in GEO and TCGA datasets (**Supplementary Figure 1**). The ERK/MAPK signal transduction pathway is one of the most common cascades in human tumors, and its anomalous activation could endow tumor cells with various abnormal phenotypes, including uncontrolled cell proliferation, metabolic reprogramming, and immune evasion (30). As shown in **Figure 1C**, compared to BEAS-2B cells, the protein levels of MAP2K7, p-ERK, and p-p38MAPK were specifically reduced in L78 and A549 cells; however, there were no apparent differences in the total protein levels of ERK and p38MAPK among these cell lines. In recent years, the development of epigenetics has revealed that methylation modifications are important factors affecting the stability of circular RNAs. Therefore, to explore the reasons for the increased expression of circ_0060927 in NSCLC cells, we undertook RIP-qPCR to detect the m6A modification levels of circ_0060927, and results showed that the m6A modification levels in L78 and A549 cells were significantly higher than those in BEAS-2B cells (**Figure 1D**).

**Figure 2:**
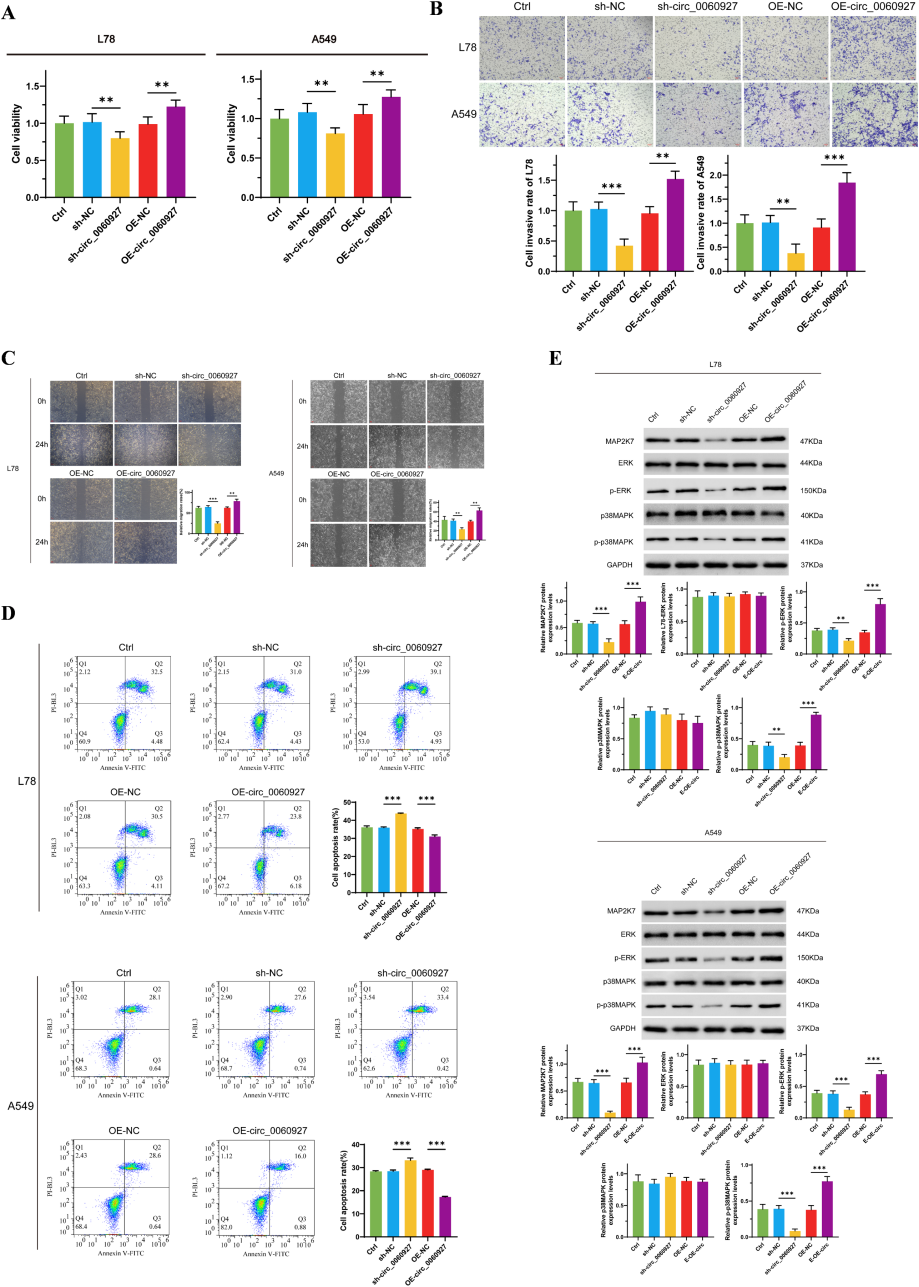
Circ_0060927 regulated NSCLC cells’ malignant biological behavior positively. **(A)** Circ_0060927 promotes L78 and A549 cells proliferation. **(B)** Circ_0060927 strengthens L78 and A549 cells invasion ability. **(C)** Circ_0060927 accelerates L78 and A549 cells migration **(D)** Circ_0060927 alleviates L78 and A549 cells apoptosis. **(E)** Circ_0060927 enhances the expression of MAP2K7 and the phosphorylation of ERK and p38MAPK. n=3 **(p < 0.01), ***(p < 0.001).

To further clarify the function of circ_0060927 in tumor cells, we knocked down and overexpressed circ_0060927 in L78 and A549 cells to observe changes in a series of malignant phenotypes. We found the proliferation capability of L78 and A549 cells with circ_0060927 knockdown significantly decreased, with cell viability reduced by more than 30% compared to the control group. In contrast, cells overexpressing circ_0060927 exhibited enhanced proliferation ability (**Figure 2A**). Additionally, through wound healing assay and Transwell experiment, we evaluated the impact of circ_0060927 on the migration and invasion tendency of NSCLC cells (**Figures 2B, C**). In contrast to the control group, the migration rate of cells with circ_0060927 knockdown obviously slowed down. The Transwell experiments further demonstrated that the cell invasion capacity with circ_0060927 knockdown was reduced, while in the circ_0060927 overexpression group, both cell migration and penetration rate were overtly enhanced, further validating the promoting function of circ_0060927 on NSCLC cell mobility. Flow cytometry made us unravel that the cell apoptosis rate with circ_0060927 knockdown was improved, while overexpressing circ_0060927 reduced the cell apoptosis rate, indicating the role of circ_0060927 in inhibiting cell apoptosis (**Figure 2D**). Interestingly, the suppression of circ_0060927 expression in L78 and A549 cells can inhibit the expression of the ERK/MAPK signaling pathway marker MAP2K7, while also reducing the phosphorylation levels of ERK and p38MAPK proteins in L78 and A549 cells. In contrast, enhancing the endogenous circ_0060927 can promote the activity of the MAP2K7/p-ERK/p-p38MAPK cascade reaction (**Figure 2E**).

### Circ_0060927 fosters NSCLC cells’ progression by targeting miR-331-3p

**Figure 3:**
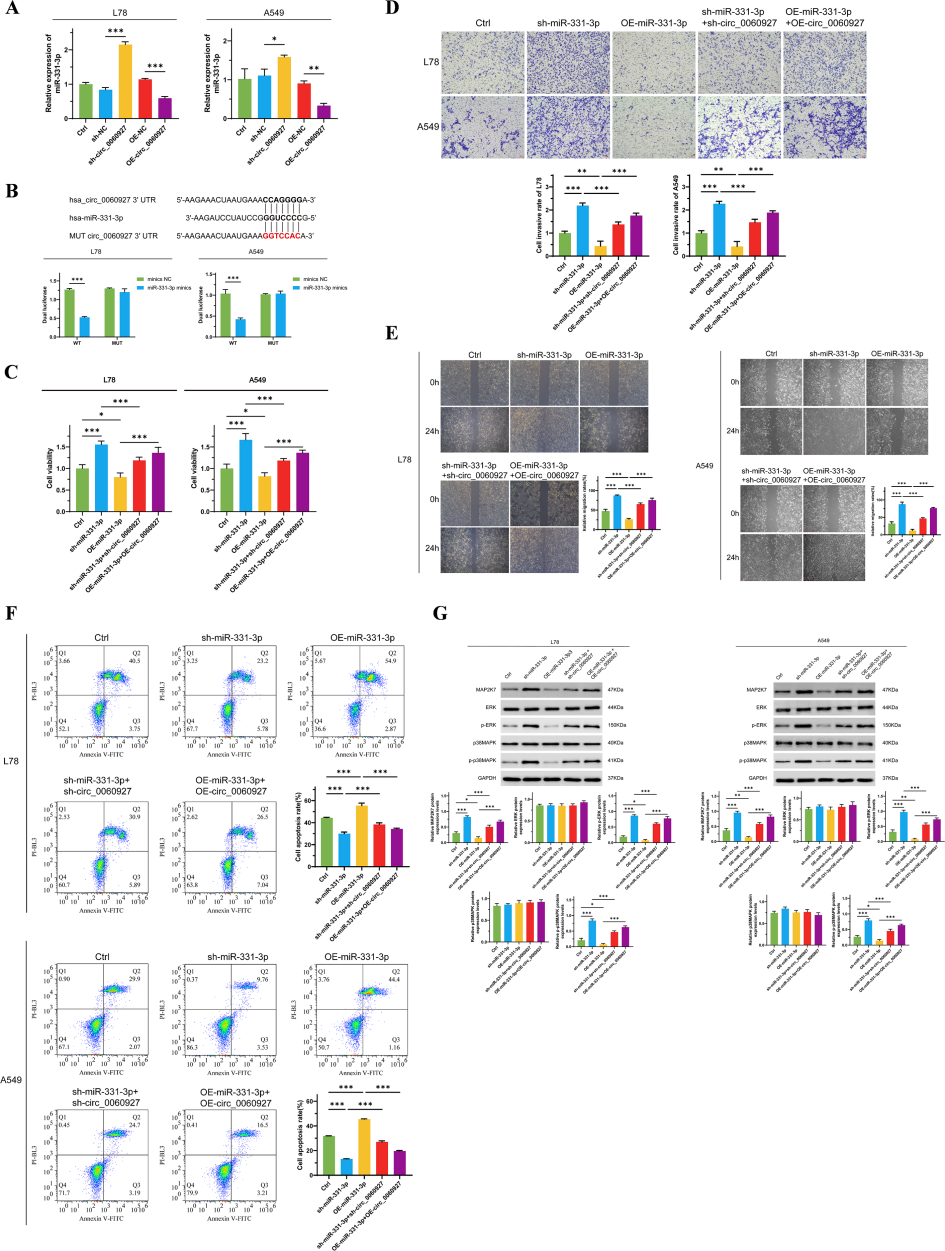
Circ_0060927 targeting binds miR-331-3p to promote NSCLC progression. **(A)** The expression of circ_0060927 was negatively correlated with the miR-331-3p level. **(B)** Dual luciferase reporter assay verified the direct binding effect between circ_0060927 and miR-331-3p. **(C)** The change in L78 and A549 cells’ proliferation after overexpression or inhibition of circ_0060927 and miR-331-3p. **(D)** L78 and A549 cells’ invasion ability alteration after overexpression or inhibition of circ_0060927 and miR-331-3p. **(E)** Cell mortality after overexpression or inhibition of circ_0060927 and miR-331-3p. **(F)** The impact of overexpression or inhibition of circ_0060927 and miR-331-3p on cell apoptosis. **(G)** The expression of MAP2K7 and the phosphorylation of ERK and p38MAPK after co-overexpression or co-inhibition of circ_0060927 and miR-331-3p in L78 and A549 cells. n=3 *(p < 0.05), **(p < 0.01), ***(p < 0.001).

Circular RNA, a special structure of ncRNA, can act as a miRNA sponge to bind miRNA, thereby interfering with the negative regulatory effect of miRNA on mRNA (31, 32). We proved that knocking down circ_0060927 expression can upregulate miR-331-3p expression in L78 and A549 cells (**Figure 3A**). Therefore, we verified the specific binding effect of circ_0060927 with miR-331-3p through dual-luciferase reporter assays. By predicting the specific binding sites of circ_0060927 and miR-331-3p according to the Circular RNA Interactome website, we constructed circ_0060927 mutant cell lines at the corresponding sites, while overexpressing miR-331-3p in both wild-type and circ_0060927 mutant cell lines. As shown in **Figure 3B**, compared to wild-type miR-331-3p high-expressing cells, tumor cells with a mutated circ_0060927 site and overexpressed miR-331-3p exhibited enhanced fluorescence levels. This indicates that there is indeed a binding interaction between circ_0060927 and miR-331-3p in L78 and A549 cells. To further validate that miR-331-3p worked as a downstream factor below circ_0060927 to affect the malignant phenotype of tumor cells, we simultaneously knocked down or overexpressed circ_0060927 and miR-331-3p molecules in L78 and A549. Undoubtedly, results found that knocking down miR-331-3p expression in L78 and A549 cells increased cell proliferation (**Figure 3C**), promoted tumor cell migration and invasion (**Figures 3D, E**). Conversely, after upregulating miR-331-3p expression, L78 and A549 cells’ viability dramatically decreased (**Figure 3C**), apoptosis levels increased (**Figure 3F**), along with a reduction in migration and invasion capabilities (**Figures 3D, E**). Interestingly, compared to cells only knocked down for miR-331-3p expression, knocking down miR-331-3p and circ_0060927 expression resulted in a significant decrease in the proliferation and motility of L78 and A549 cells (**Figures 3C-E**), along with an increase in apoptotic cell numbers (**Figure 3F**). Similarly, compared to cells with only overexpressed miR-331-3p, simultaneously overexpressing miR-331-3p and circ_0060927 facilitated cell multiplication and migration (**Figures 3C-E**) while inhibiting apoptosis levels (**Figure 3F**). Unsurprisingly, the decline of tumor cell miR-331-3p expression promoted the MAP2K7/p-ERK/p-p38MAPK signaling cascade, while the superfluous miR-331-3p downregulated the protein level and activity of this signaling axis factors; however, simultaneously inhibiting or overexpressing miR-331-3p and circ_0060927 reversed the changes in the activity of the MAP2K7/p-ERK/p-p38MAPK signaling axis induced by manipulating miR-331-3p (**Figure 3G**). The above results confirm that circ_0060927 can factually accelerate the malignant biological behavior by targeted binding with miR-331-3p in NSCLC.

### miR-331-3p prohibits NSCLC cell malignant phenotypes by targeting the MAPK pathway

**Figure 4:**
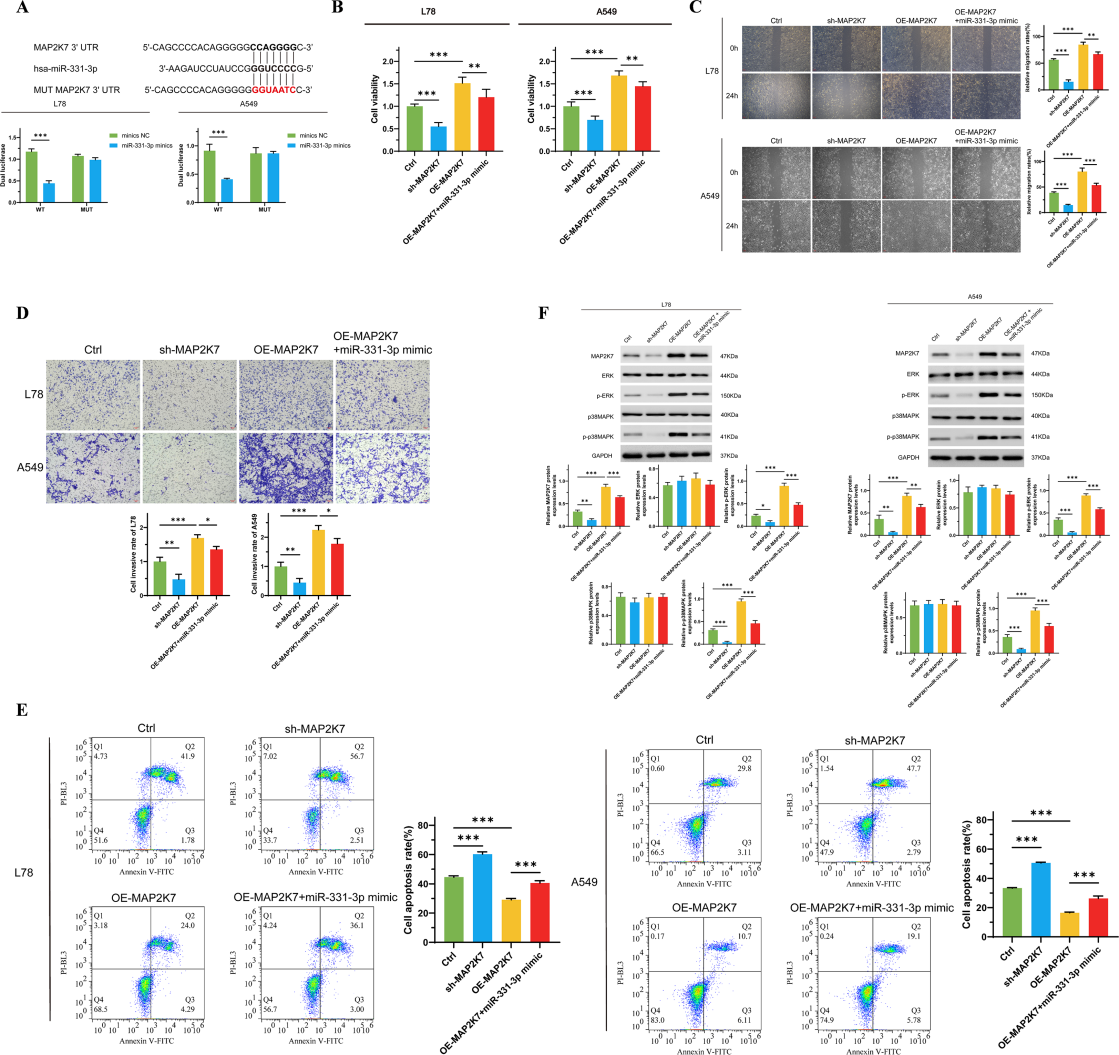
MiR-331-3p impedes NSCLC cells’ malignant biological behaviors through suppressing the ERK/MAPK signaling pathway. **(A)** Dual luciferase reporter assay verified the direct binding effect between miR-331-3p and MAP2K7. **(B)** The change of L78 and A549 cells’ proliferation after overexpression of MAP2K7 and miR-331-3p. **(C)** L78 and A549 cells’ invasion ability alteration after overexpression of MAP2K7 and miR-331-3p. **(D)** Cell mortality after overexpression of MAP2K7 and miR-331-3p. **(E)** The impact of MAP2K7 and miR-331-3p overexpression on cell apoptosis. **(F)** The expression of MAP2K7 and the phosphorylation of ERK and p38MAPK after MAP2K7 and miR-331-3p overexpression in L78 and A549 cells. n=3 *(p < 0.05), **(p < 0.01), ***(p < 0.001).

The vigor of the ERK/MAPK pathway is associated with mediating various malignant biological behaviors of tumor cells (33, 34). There is also evidence that this signaling pathway is a potential effector for miR-331-3p (22). Therefore, we predicted the binding site of MAP2K7 with miR-331-3p using the TargetScan online tool. MAP2K7, an ingredient of the MAP kinase kinase family, can specifically activate MAPK and ERK (35). Through dual-luciferase reporter assays, we constructed cell lines with MAP2K7 binding site mutations in L78 and A549 cells, while overexpressing miR-331-3p in both wild-type and mutated cells. We found that compared to wild-type miR-331-3p high-expressing cells, tumor cells with binding site mutations exhibited higher fluorescence levels (**Figure 4A**), indicating the specific binding of miR-331-3p to MAP2K7 protein in NSCLC cells. Subsequently, to verify the biological impact of this binding, we carried out a series of phenotypic experiments. Compared to cells overexpressing MAP2K7 alone, we observed reduced cell proliferation (**Figure 4B**), uplifted apoptosis (**Figure 4E**), and decreased cell migration (**Figure 4C**) and invasion (**Figure 4D**) in tumor cells co-overexpressing MAP2K7 and miR-331-3p. As demonstrated by Western Blotting experiments, the phosphorylation levels of ERK and p38MAPK were increased in cells overexpressing MAP2K7, while co-overexpression of miR-331-3p could reverse the phosphorylation levels of ERK and p38MAPK (**Figure 4F**). The above results indicate that in non-small cell lung cancer cells, miR-331-3p inhibits the malignant phenotypes of tumor cells by suppressing the activity of the ERK/MAPK signaling pathway.

### METTL14 drives m6A modification

**Figure 5:**
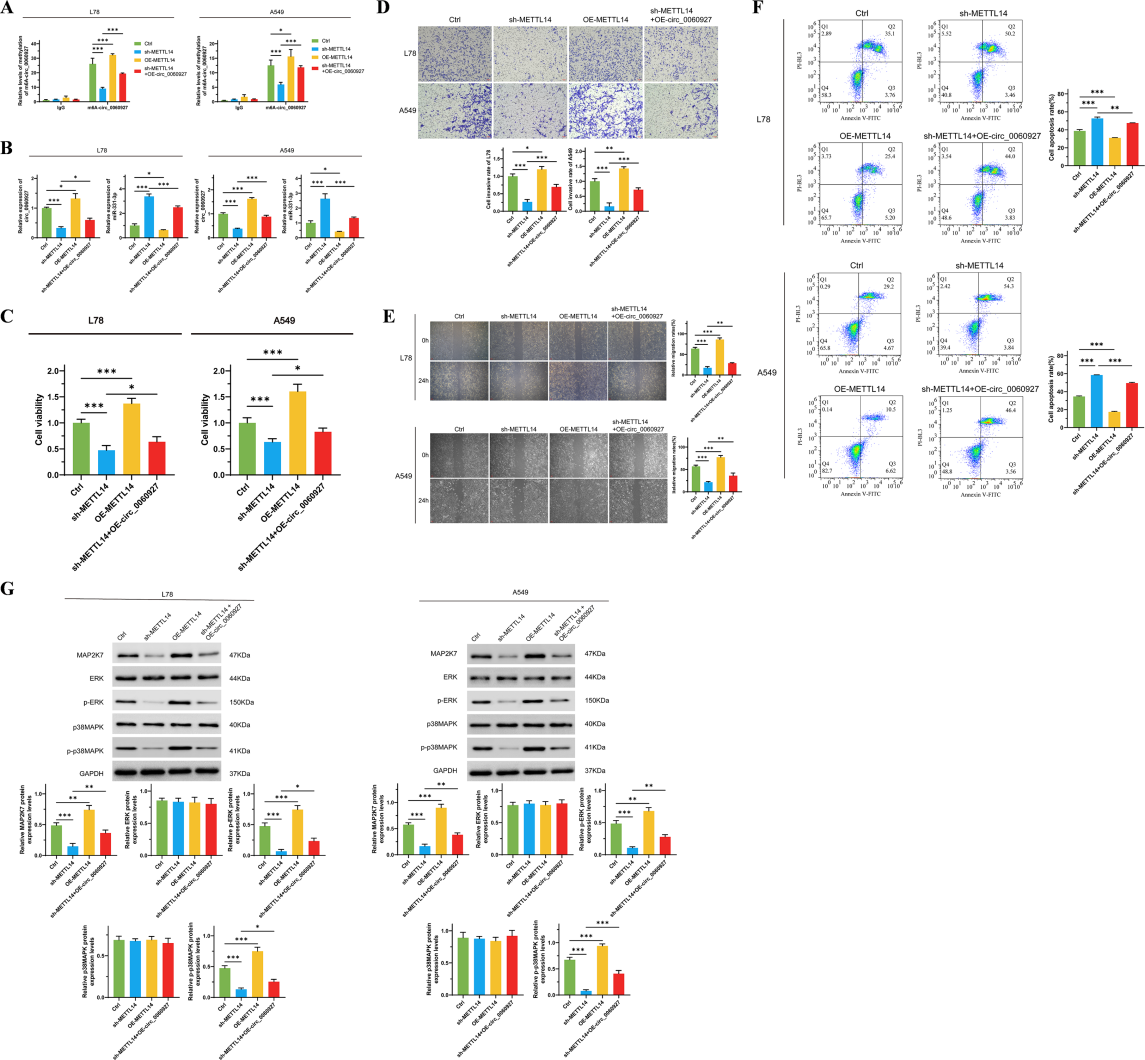
METTL4 promotes the intracellular expression of circ_0060927 based on inducing circ_0060927 m6A methylation modification. **(A)** The regulatory relation between the m6A methylation level of circ_0060927 and METTL14. **(B)** METTL14 modulated miR-331-3p expression negatively by enhancing circ_0060927 expression level. **(C)** METTL14 induced L78 and A549 cells’ proliferation by targeting circ_0060927. **(D)** METTL14 facilitated L78 and A549 cells invasion ability by targeting circ_0060927. **(E)** METTL14 accelerated L78 and A549 cells movement by targeting circ_0060927. **(F)** Circ_0060927 restored cell apoptosis which was prohibited by METTL14 overexpression. **(G)** The influence of METTL14 on ERK/MAPK signaling cascade. n=3 *(p < 0.05), **(p < 0.01), ***(p < 0.001).

To further clarify the high m6A level of circ_0060927 originates from the methylation modification mediated by METTL14, we manipulated the expression of METTL14 positively and negatively in L78 and A549 cells, respectively. We found that after inhibiting METTL14 expression, the circ_0060927 m6A modification level significantly decreased; conversely, overexpression of METTL14 could upregulate the circ_0060927 m6A modification level (**Figure 5A**) in tumor cells. Increasing endogenous expression of circ_0060927 while inhibiting METTL14 expression could partially restore the m6A modification level (**Figure 5A**) of circ_0060927 in L78 and A549 cells. Down-regulated METTL4 expression was accompanied by the reduction of circ_0060927 and an increase of miR-331-3p, but circ_0060927 overexpression could also convert the change of expression of circ_0060927 and miR-331-3p induced by METTL4 impairment (**Figure 5B**). Similar to previous results, inhibiting METTL14 expression could impair the proliferation ability (**Figure 5C**) and motility of tumor cells (**Figures 5D, E**), promoting an increase in cell apoptosis (**Figure 5F**); at the same time, it downregulated the expression of MAP2K7, p-ERK, and p-p38MAPK molecules (**Figure 5G**). Upregulating the expression level of circ_0060927 could reverse the damage caused by METTL14 deficiency in L78 and A549 cells, including enhanced cell viability and motility (**Figures 5C-E**), decreased apoptosis levels (**Figure 5F**), and promoted the expression of MAP2K7 protein as well as the phosphorylation of ERK and p38MAPK proteins (**Figure 5G**). The above evidence confirmed that METTL14 is an upstream driving factor for the high methylation of circ_0060927 in non-small cell lung cancer cells.

## Discussion

The current landscape of research on NSCLC emphasizes the significant and multifaceted role of circRNAs in the intricate processes of tumor development and progression, highlighting their importance in the field of oncology (36, 37). Notably, circ_0060927 has been identified as a crucial factor in modulating the activity of miR-331-3p specifically in colorectal cancer, underscoring its potential implications in various malignancies (21). In this research, we first demonstrate that circ_0060927 is markedly upregulated in NSCLC cell lines, specifically L78 and A549, when compared to normal human lung epithelial BEAS-2B cells, thereby validating its potential role as an oncogene that could contribute to tumorigenesis. CircRNAs participate in regulating a wide array of tumor biological processes through various mechanisms, which include the regulation of gene expression and interactions with proteins, thereby influencing cellular behavior and tumor dynamics (38). For instance, circRNA hsa_circ_0008035 has been found to promote the proliferation and invasion of gastric cancer cells by adsorbing miR-375, illustrating the diverse functional roles these molecules can play in different cancer types (39). Interestingly, the expression levels of circRNAs exhibit specificity across various types of cancer; for example, circRNA ITCH is recognized as a tumor suppressor in multiple malignancies, showcasing the complex and sometimes contradictory roles that circRNAs can embody within the tumor microenvironment (40). This dualistic nature of circRNAs in tumors drives an increasing interest and in-depth research into their diverse functions and potential therapeutic implications.

Our results demonstrate that circ_0060927 directly targets miR-331-3p, which subsequently leads to significant changes in the expression levels of MAP2K7 and various components of the ERK/MAPK signaling pathway, a crucial network involved in cellular processes. Following the knockdown of circ_0060927, we observed a notable upregulation of miR-331-3p, which strongly supports the idea that circ_0060927 functions as a molecular sponge, effectively sequestering miR-331-3p and thereby influencing the intricate downstream signaling pathways that are vital for cellular communication and response mechanisms. MiRNA is a flock of small RNA molecules that play a paramount role in gene expression modulation by binding to mRNA targets, inhibiting its translation or leading to its degradation (41). CircRNA targeting miRNA is one of the important mechanisms of gene expression regulation (42, 43). The interaction between circRNA and miRNA in cancer has been widely studied. In breast cancer, circRNA hsa_circ_001783 inhibits miR-200c-3p through the sponge effect, promoting the proliferation and invasion of tumor cells (44). 256 differentially expressed circRNAs have been found to have significant interactions with 20 miRNAs in pancreatic ductal adenocarcinoma (45). These interactions may be involved in tumor development by affecting pathways such as cell proliferation and apoptosis. By constructing a circRNA-miRNA-mRNA regulatory network, researchers are able to identify specific circRNAs and miRNAs associated with tumors, providing new directions for the development of tumor biomarkers (46).

METTL14 is one of the key enzymes for m6A methylation and is an important component of the m6A methyltransferase complex (MTC) (47). METTL14 promotes the addition of m6A to RNA molecules by recognizing specific RNA sequences, a process that has a significant impact on RNA stability and translation efficiency (48, 49). METTL14 exerts a dual impact in the occurrence and progression of various cancers, acting else as a tumor suppressor gene or as an oncogene, with its specific function depending on the type of tumor and its microenvironment (50). In gastric cancer and breast cancer, lower grade of expression of METTL14 is associated with poorer prognosis, while overexpression of METTL14 in certain types of tumors is related to tumor proliferation and metastasis (50, 51). Additionally, the expression patterns of METTL14 vary significantly across different types of tumors. For example, METTL14 expression in lung cancer is higher than that in normal lung tissue and is associated with aggressiveness and metastatic ability of the tumor (52). Nevertheless, the downregulation of METTL14 is considered an important factor in the tumorigenesis and metastasis of breast cancer cells, possibly functioning relies on affecting the factors’ expression related to the Wnt signaling pathway (53). In lymphoma, the expression of METTL14 is also considered an important prognostic indicator, which related to the degree of tumor differentiation and patients’ survival rate, with low expression of METTL14 potentially indicating poorer clinical outcomes (54). During this research, we meticulously verified that the methylation mediated by METTL14 serves as the fundamental reason behind the notably high expression levels of circ_0060927. The alteration of m6A levels plays a crucial role in affecting both the stability and expression of circ_0060927, which in turn has significant implications for the regulation of miR-331-3p and the ERK/MAPK signaling pathway. Although we did not generate m6A-deficient mutants of circ_0060927 to directly verify causality, previous studies provide strong evidence that m6A methylation plays a critical regulatory role in circRNA biology. In hepatocellular carcinoma, METTL14 directly binds to and installs m6A on circSTX6, and the regulatory effect disappears when m6A sites are mutated, indicating METTL14-dependent m6A regulation of circRNA (55). Mechanistically, m6A promotes circRNA turnover via YTHDF2-mediated endoribonuclease cleavage, to increase circRNA stability upon ALKBH5 modulation, and to drive circRNA translation in an m6A-dependent manner. Consistent with these observations, reducing global m6A or knocking down METTL14 decreases circ-ZNF609 abundance and stability *in vivo* and *in vitro*, underscoring that METTL14-m6A might be closely related to circRNA stability and function (56). In addition, studies in bladder cancer demonstrate that specific circRNAs can scaffold the WTAP/METTL3/METTL14 complex to remodel m6A on target mRNAs and thereby modulate chemosensitivity, highlighting the potential bidirectional interplay between circRNAs and the m6A machinery in cancer (57). This complex interplay of molecular regulators and their associated phenotypic outcomes underscore the potential for targeting this regulatory axis. However, our study did not directly evaluate whether m6A modification is indispensable for circ_0060927’s oncogenic function, which remains a key limitation. In future work, we aim to generate m6A-site-mutant constructs of circ_0060927 and assess their functional consequences, to definitively determine whether METTL14-dependent m6A methylation is causally required for its role in NSCLC progression.

Despite these intriguing facts, this study still confronts several significant limitations that must be carefully considered and taken into account. First, the primary focus on only two NSCLC cell lines, specifically L78 and A549, and all experiments were performed *in vitro*. The absence of animal models prevents us from validating the functional role of circ_0060927 and the METTL14-mediated circ_0060927/miR-331-3p/ERK-MAPK axis within a physiological context. This limitation leaves a gap in understanding how this regulatory cascade operates in the complex tumor microenvironment *in vivo*. Secondly, the present study did not include data from primary NSCLC patient tissues, which restricts the direct clinical extrapolation of our findings. Although we attempted to address this issue by analyzing publicly available GEO and TCGA datasets, where circ_0060927’s host gene CYP24A1 was consistently elevated in lung cancer samples, the absence of actual validation in patient remains a notable limitation. Thirdly, while our findings identify circ_0060927 as a potential oncogenic driver and a candidate therapeutic target in NSCLC, the long-term implications of its inhibition remain to be explored. To enhance the relevance and applicability of METTL14 and circRNA as promising novel therapeutic targets for the treatment of NSCLC, future research might encompass a broader range of cell types, patient-derived samples, and *in vivo* models. Given the complexity of circular RNA biology, including their high stability, diverse subcellular localization, and potential off-target effects, future studies might focus on evaluating the durability, specificity, and safety of circ_0060927-targeted interventions in preclinical models. Moreover, the development of delivery platforms, such as circRNA-specific antisense oligonucleotides or nanoparticle-based inhibitors, will be critical to translate these findings into viable therapeutic strategies. This comprehensive approach may further elucidate the METTL14-associated mechanisms, while also proving the methylation changes of circRNA under various physiological and pathological conditions to clarify its biological functions and therapeutic potential.

## Conclusion

In summary, our study reveals that METTL14-mediated m6A modification promotes the expression of circ_0060927, which functions as a ceRNA by sponging miR-331-3p and activating the MAP2K7/ERK-MAPK pathway in NSCLC cells. This highlights a novel epitranscriptomic regulatory axis involving circRNA and m6A methylation in lung cancer progression. These findings provide new insights into the functional role of methylated circRNAs in NSCLC and suggest potential targets for therapeutic intervention. However, the clinical translation of these findings remains challenging. Efficient and specific targeting of circular RNAs *in vivo* remains technically challenging due to their covalently closed loop structures and high stability. The delivery of RNA-based therapeutics to lung tissues without eliciting immune responses or off-target effects requires the development of safe and lung-targeted delivery systems. Future work is needed to evaluate the safety, specificity, and delivery strategies for targeting circ_0060927 *in vivo*, as well as to determine its expression heterogeneity across NSCLC subtypes. Addressing these issues is essential to assess the long-term therapeutic potential of circ_0060927-based interventions.

## Data Availability

The raw data supporting the conclusions of this article will be made available by the authors, without undue reservation.
