# Supplementary material for: Circ_0060927 regulates miR-331-3p/ERK/MAPK pathway reaction in non-small cell lung cancer through METTL14-driven methylation
Source: Front Oncol. 2025 Nov 13;15:1609215. doi: 10.3389/fonc.2025.1609215 (PMC12657173; doi:10.3389/fonc.2025.1609215)
Supplement: Supplementary file 1 [file Supplementaryfile1.docx]

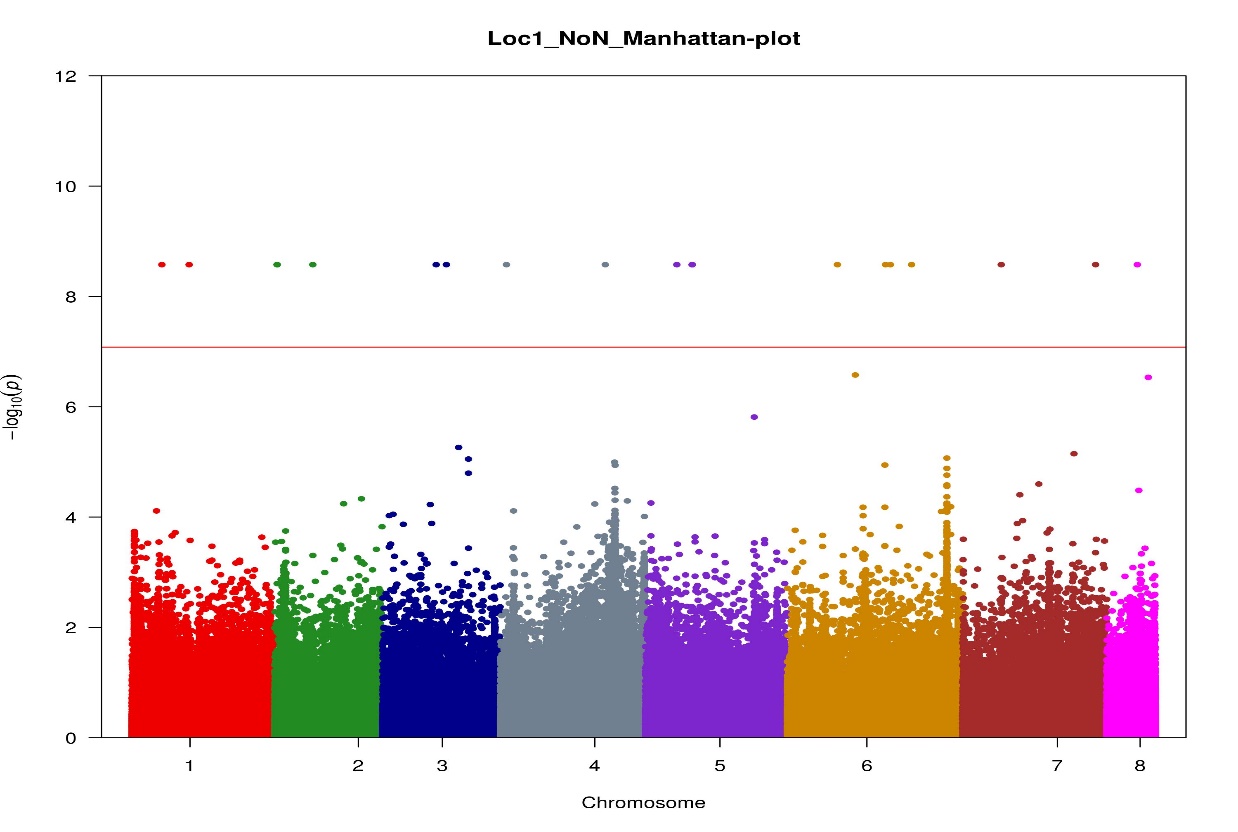


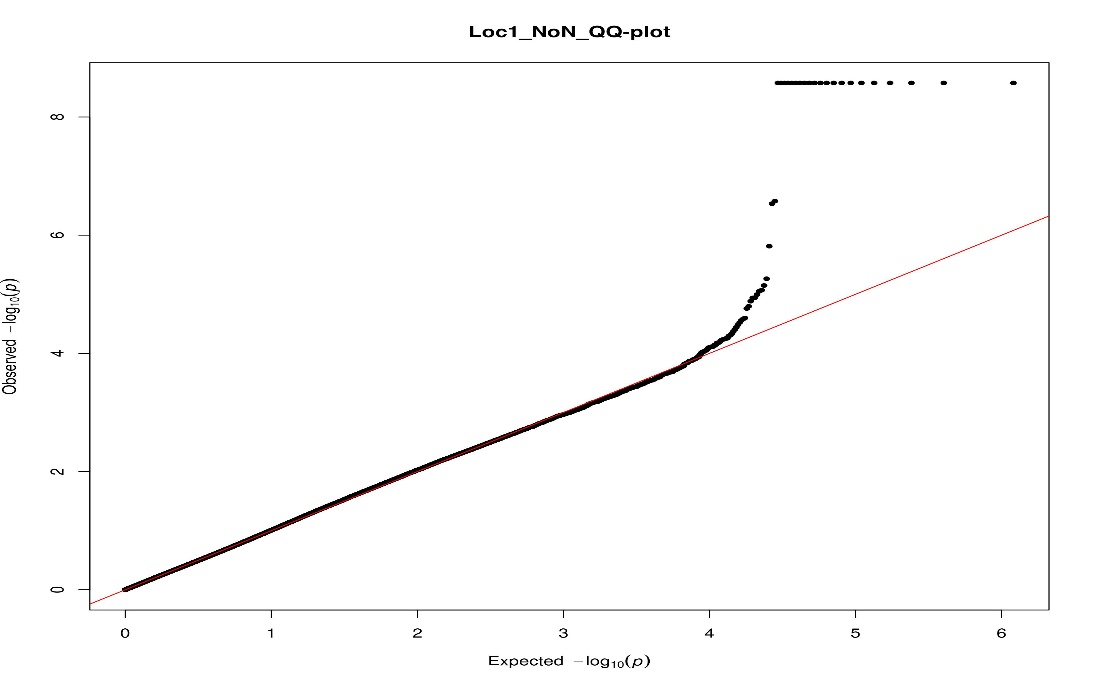


**Supplementary figure 1(a)**: Manhattan plot illustrating SNPs linked to number of nodules with their corresponding statistical significance represented by Q-Q plot for location 1 -Blink model


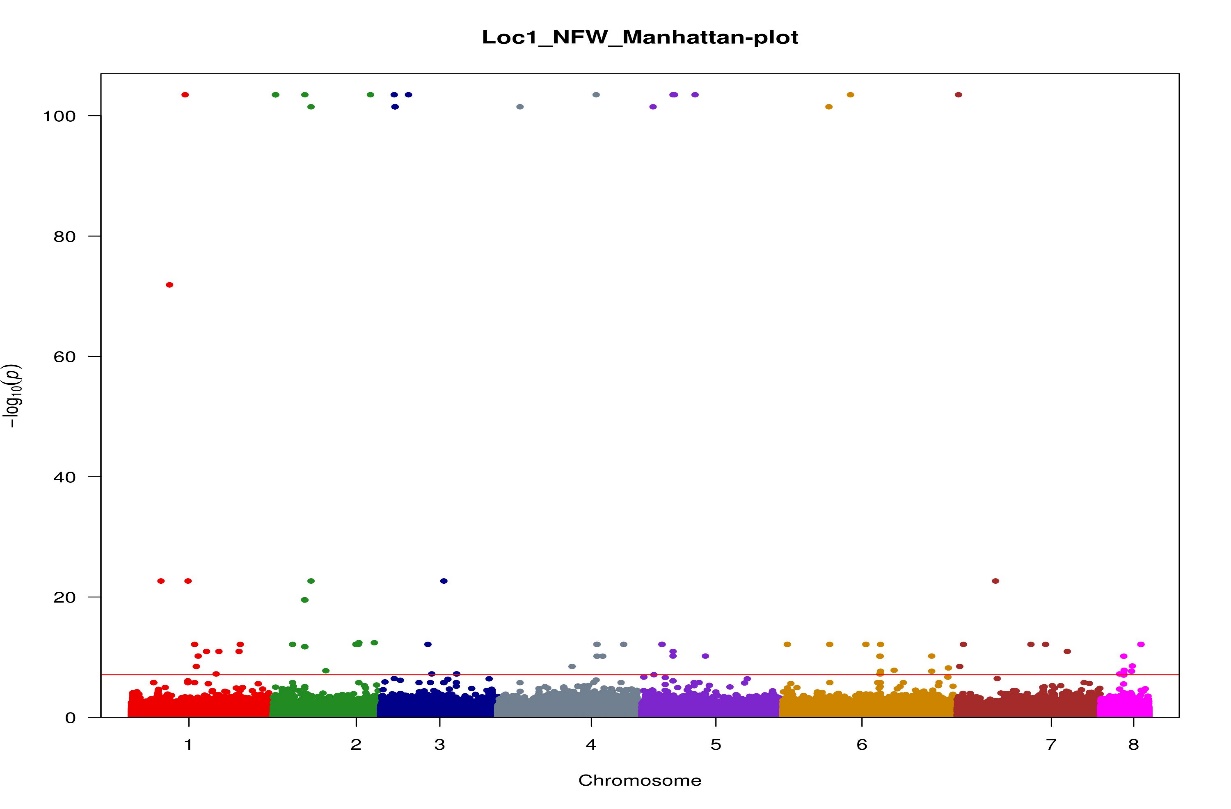


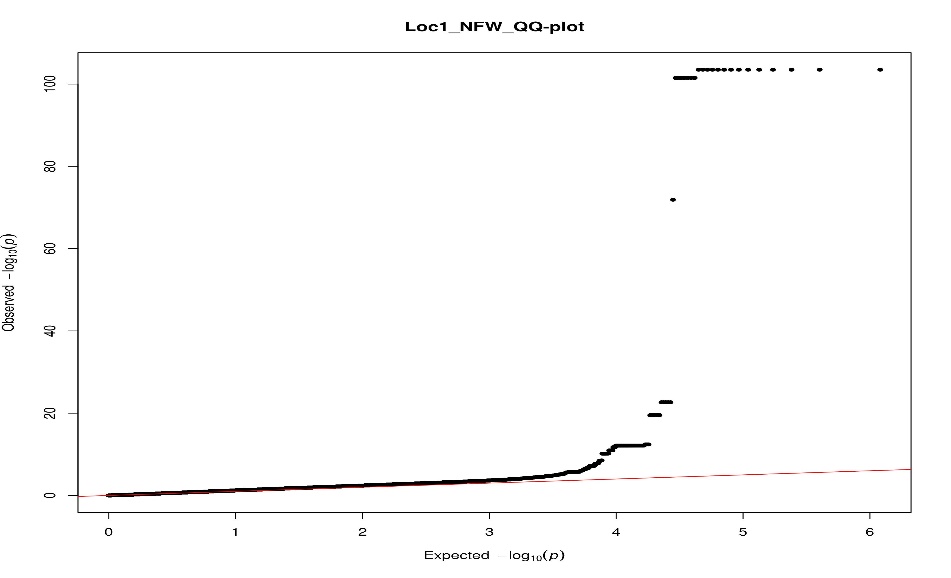


**Supplementary figure 1(b)**: Manhattan plot illustrating SNPs linked to nodule fresh weight with their corresponding statistical significance represented by Q-Q plot for location 1 -Blink model


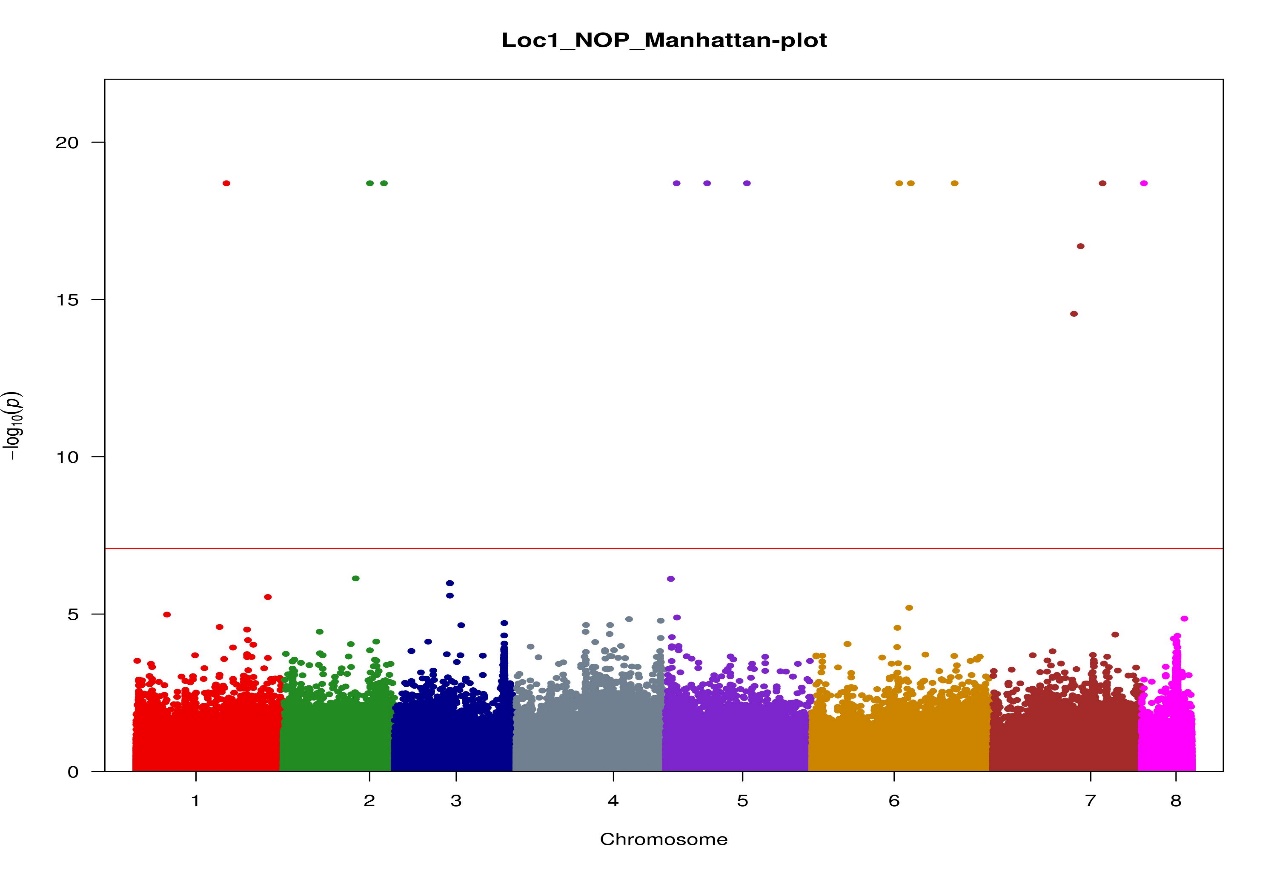


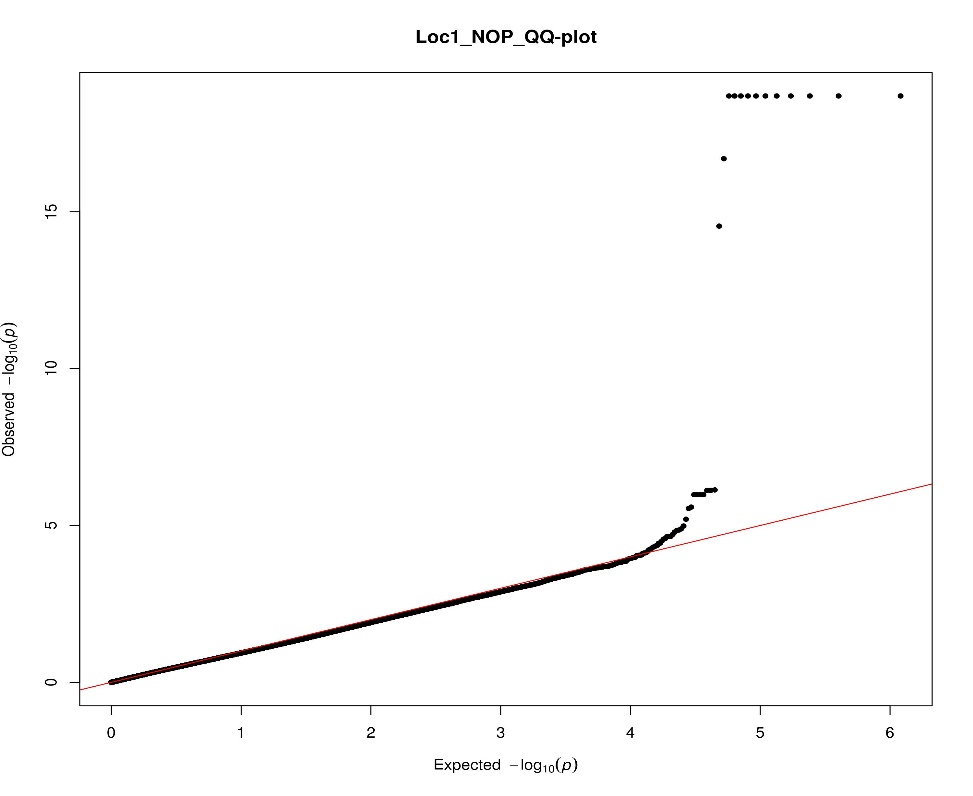


**Supplementary figure 1(c): Manhattan plot illustrating SNPs linked to number of pods with their corresponding statistical significance represented by Q-Q plot for location 1 -Blink model**


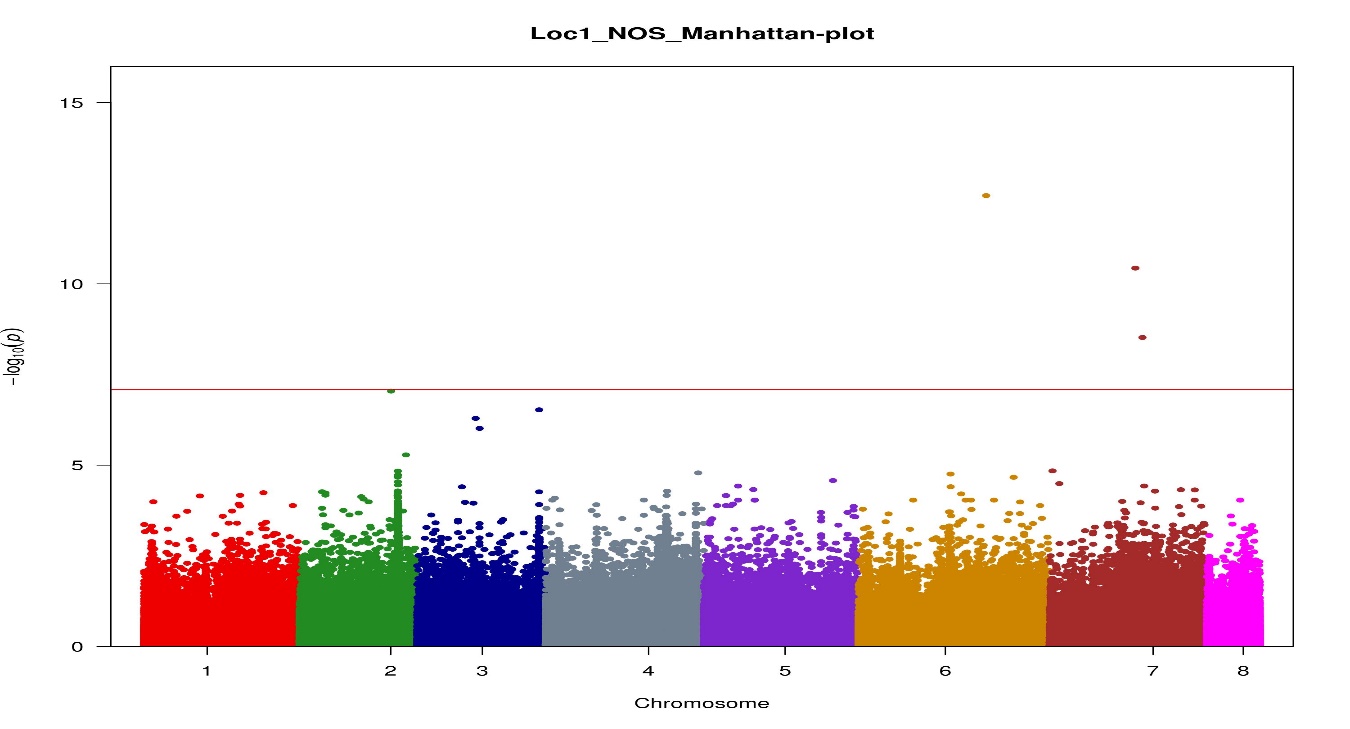


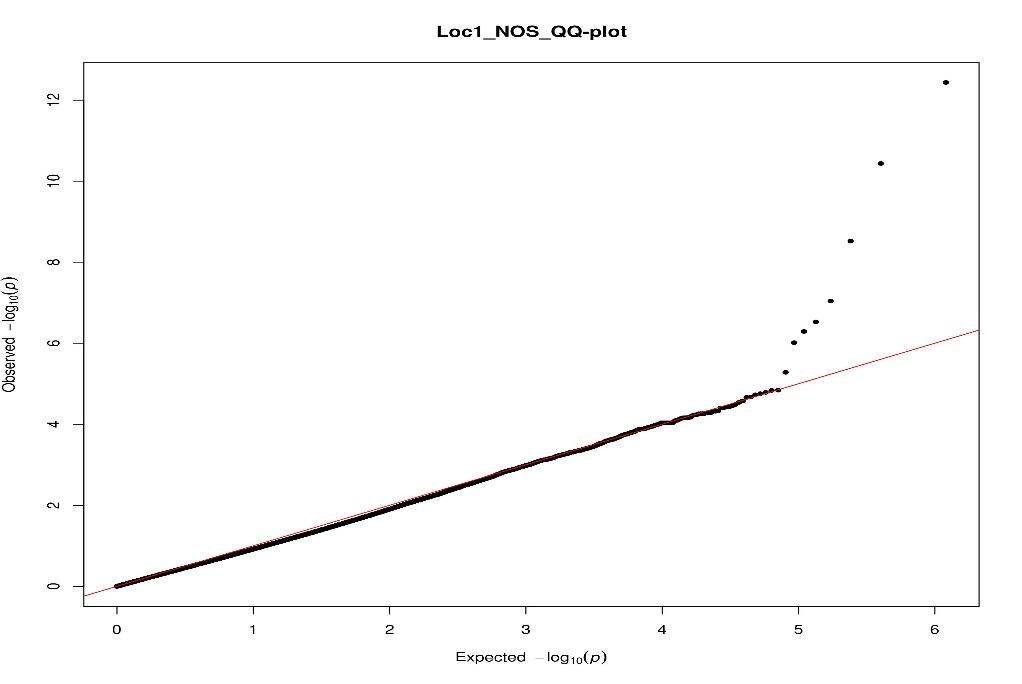


**Supplementary figure 1(d): Manhattan plot illustrating SNPs linked to number of seeds with their corresponding statistical significance represented by Q-Q plot for location 1 -Blink model**


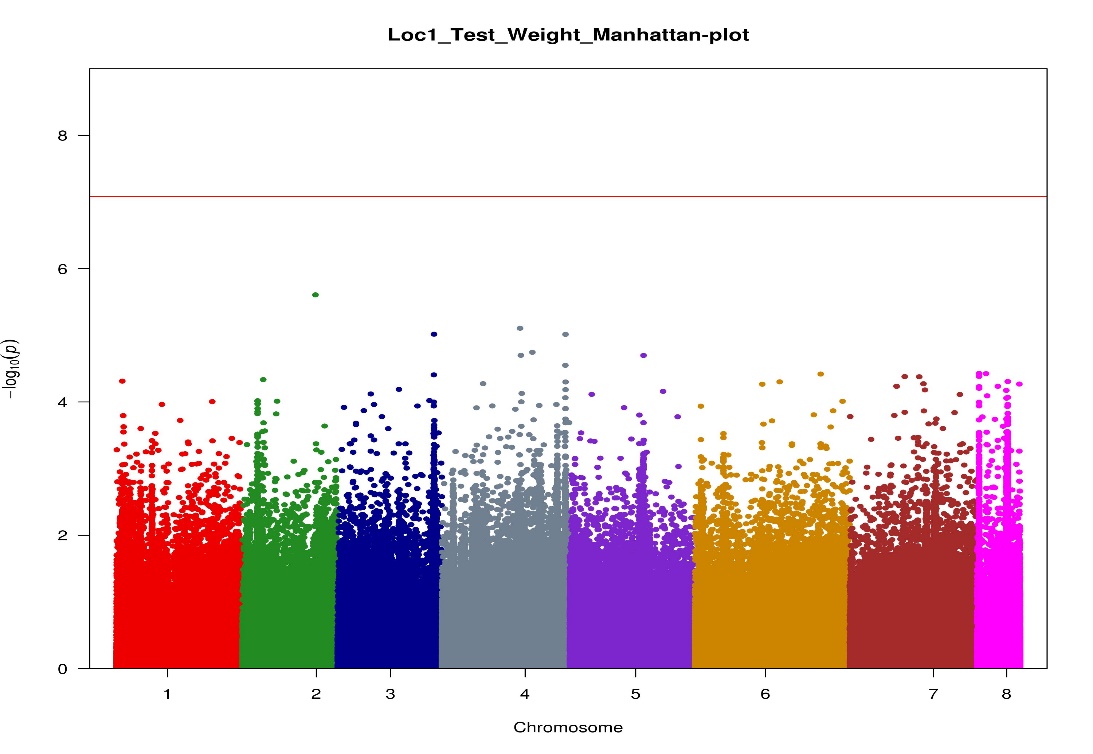


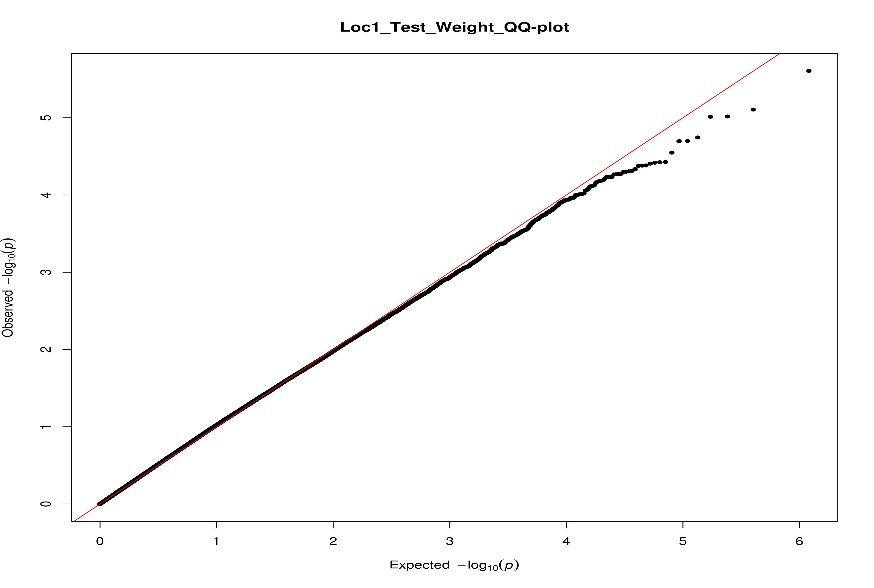


**Supplementary figure 1(e): Manhattan plot illustrating SNPs linked to test weight with their corresponding statistical significance represented by Q-Q plot for location 1 -Blink model**


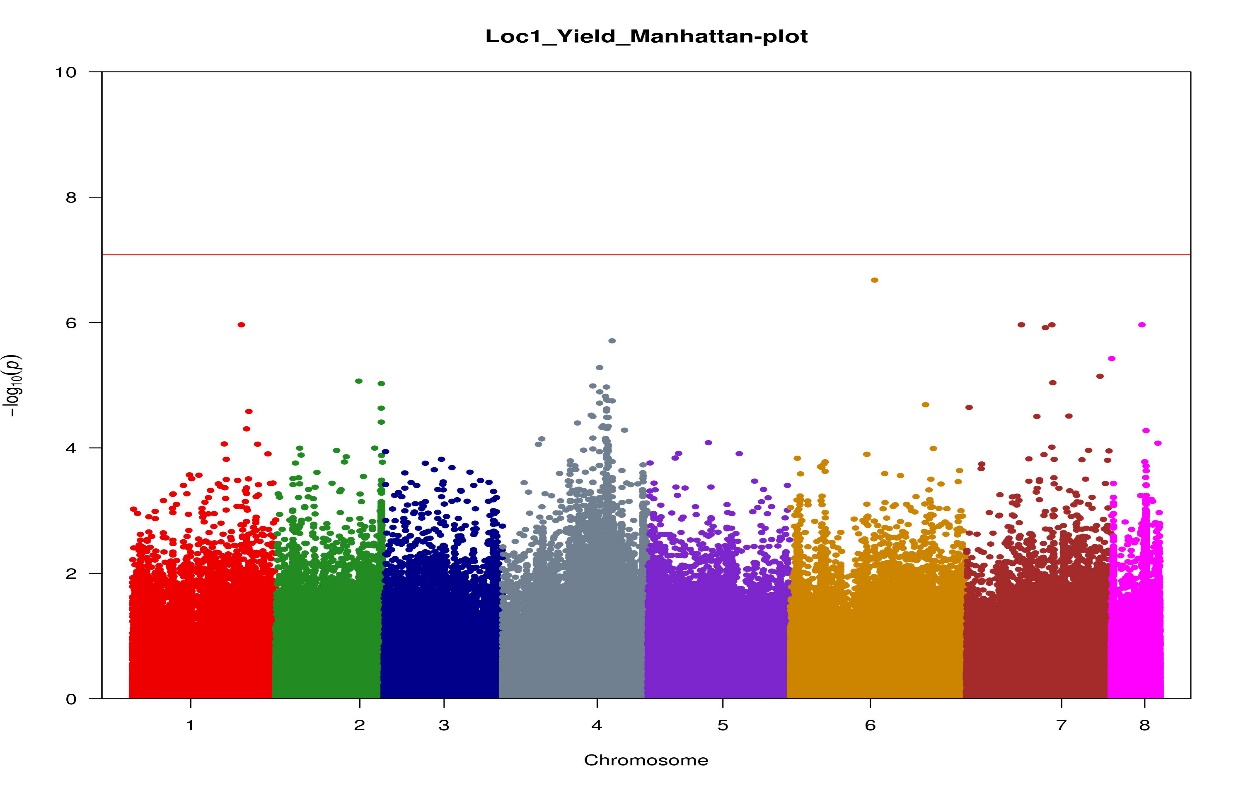


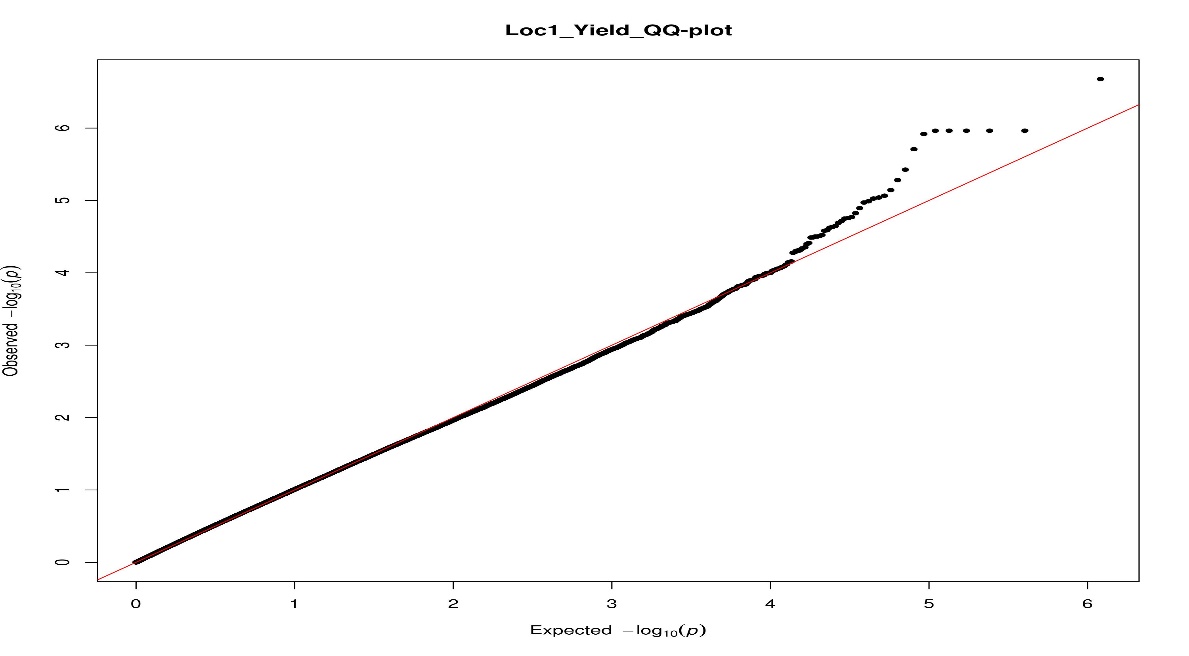


**Supplementary figure1(f): Manhattan plot illustrating SNPs linked to yield with their corresponding statistical significance represented by Q-Q plot for location 1 -Blink model**


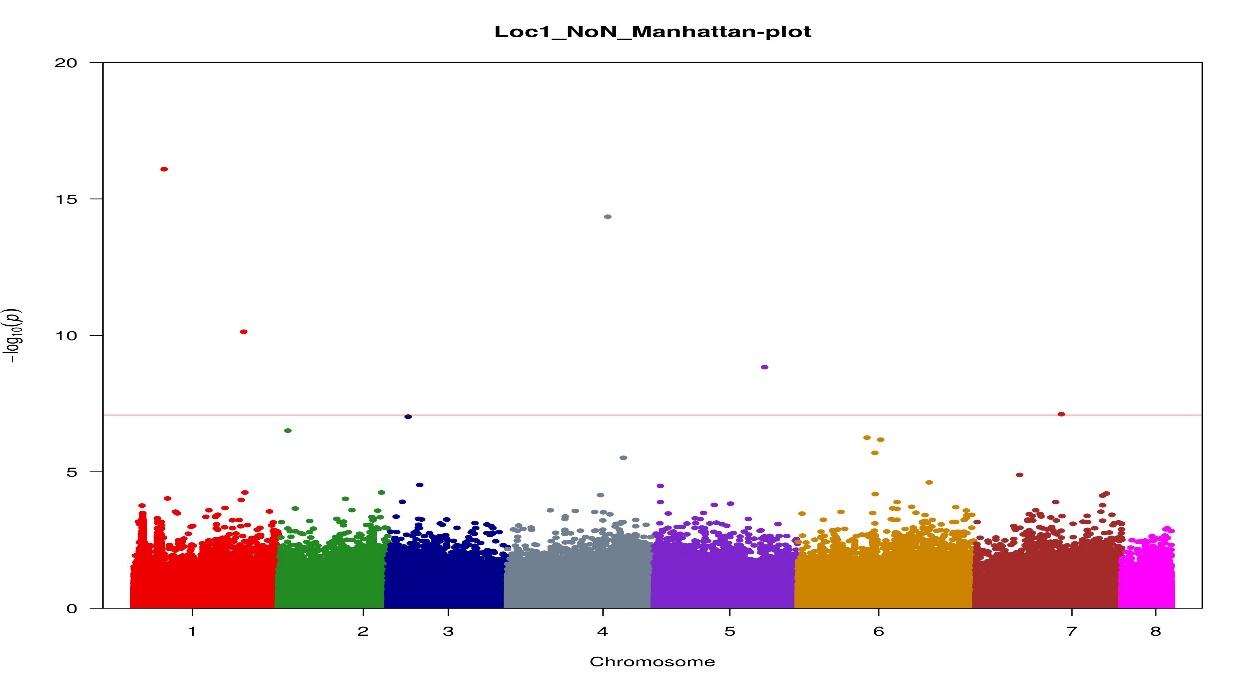


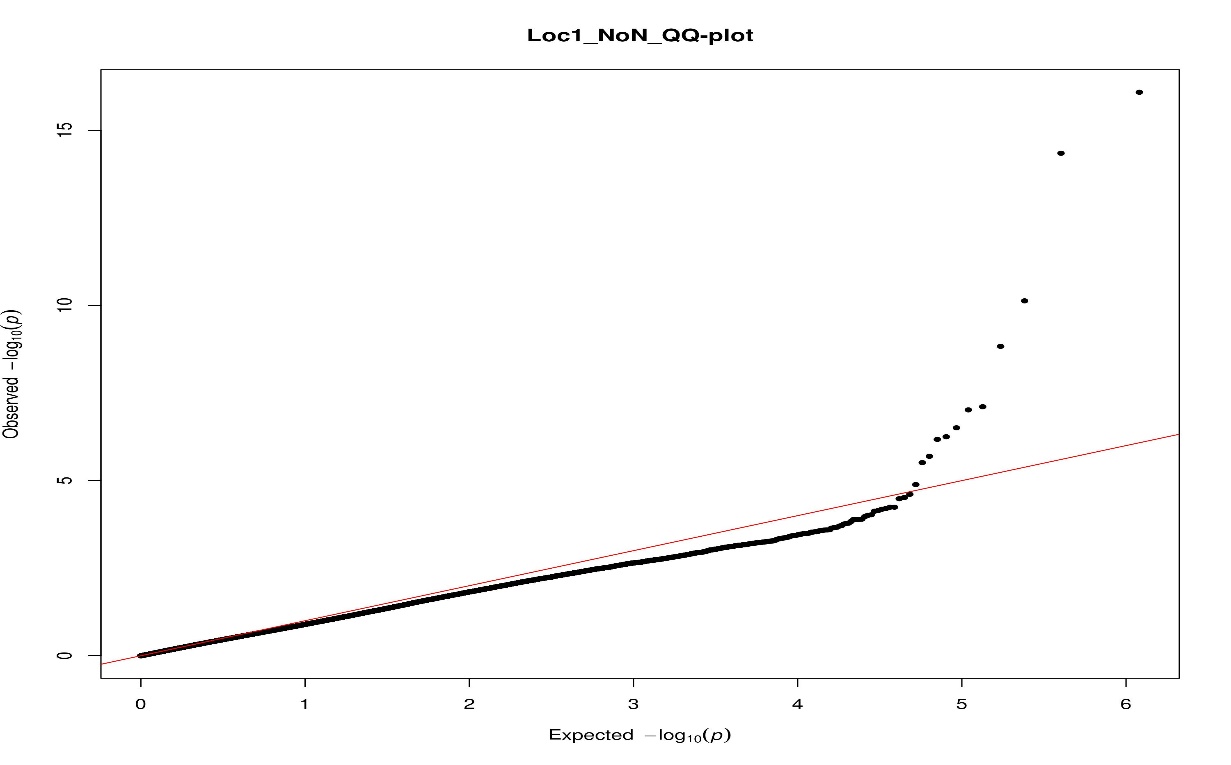


**Supplementary figure 2(a): Manhattan plot illustrating SNPs linked to number of nodules with their corresponding statistical significance represented by Q-Q plot for location 1 -FarmCPU model**


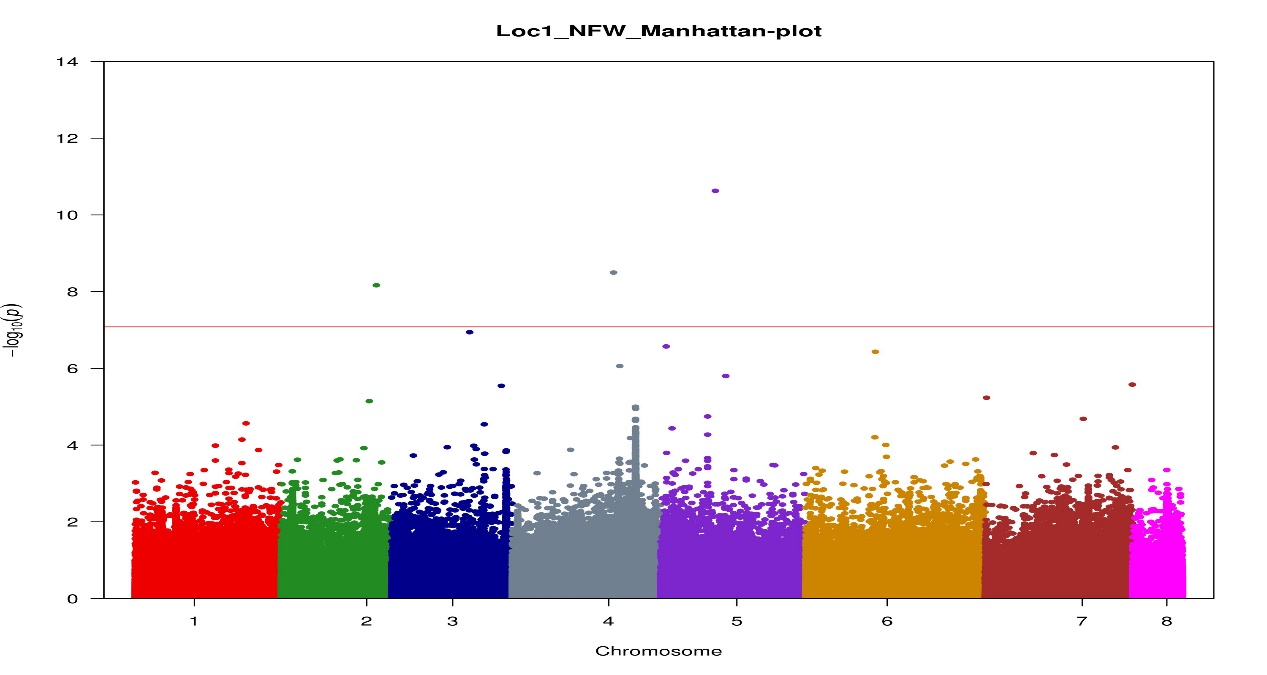


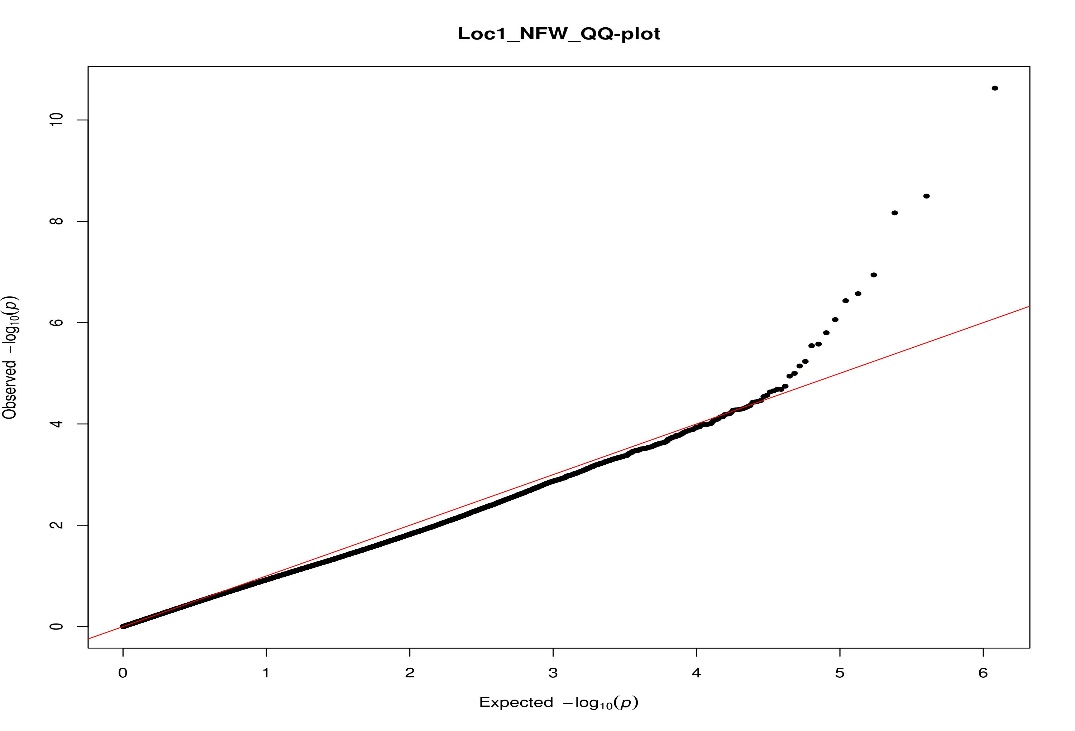


**Supplementary figure 2(b): Manhattan plot illustrating SNPs linked to nodule fresh weight with their corresponding statistical significance represented by Q-Q plot for location 1 -FarmCPU model**


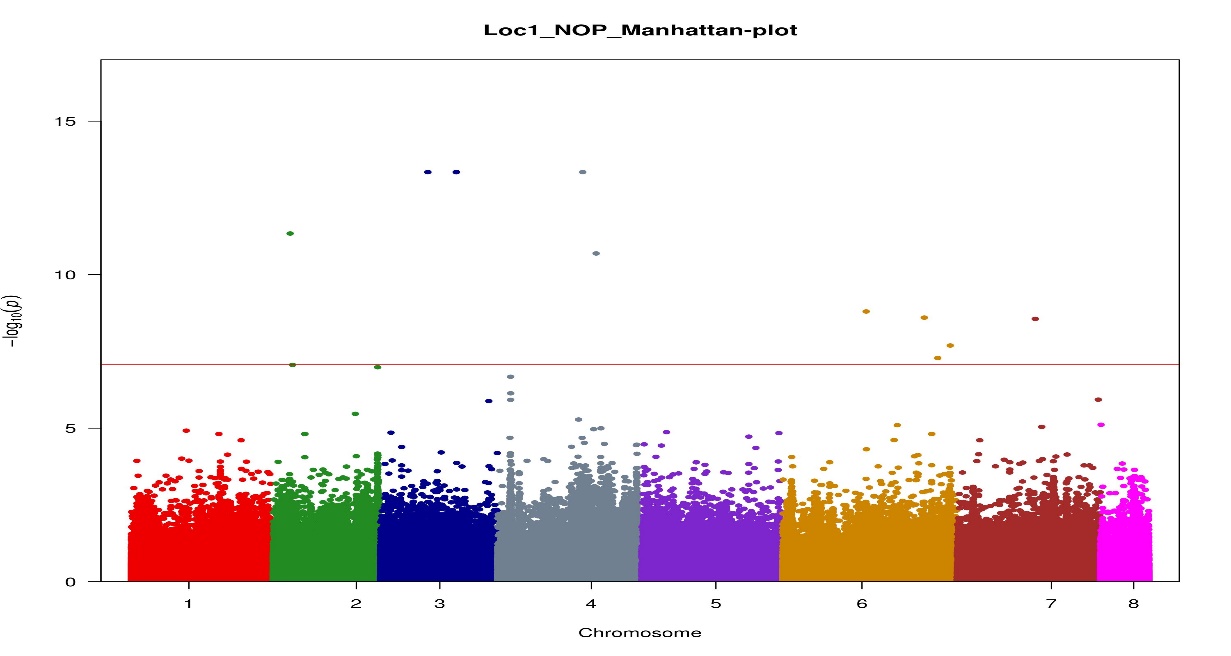


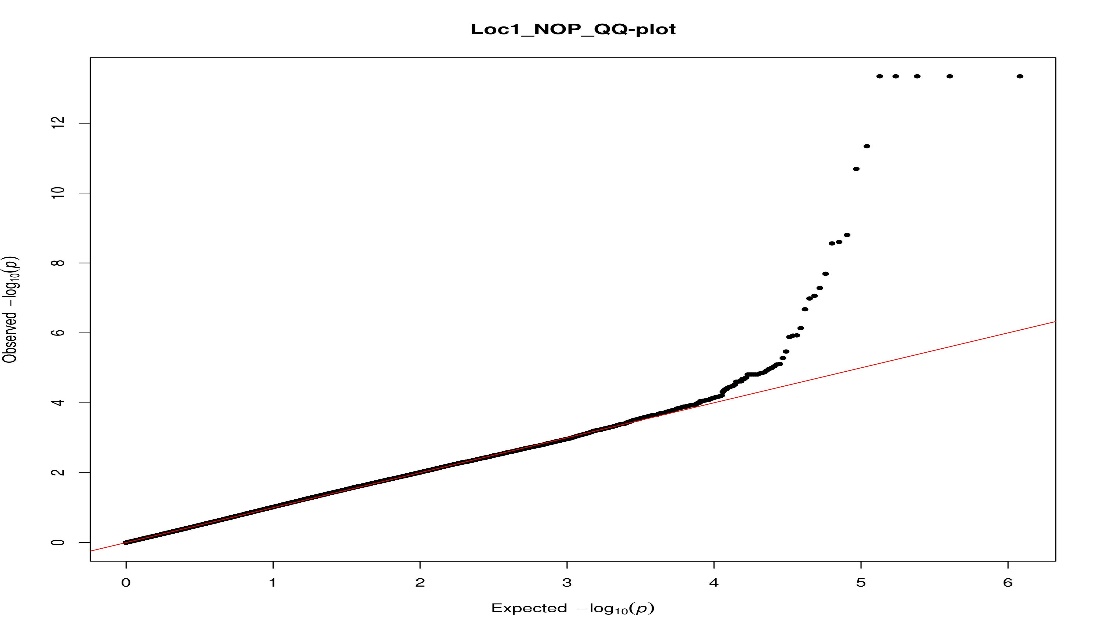


**Supplementary figure 2(c): Manhattan plot illustrating SNPs linked to number of pods with their corresponding statistical significance represented by Q-Q plot for location 1 -FarmCPU model**


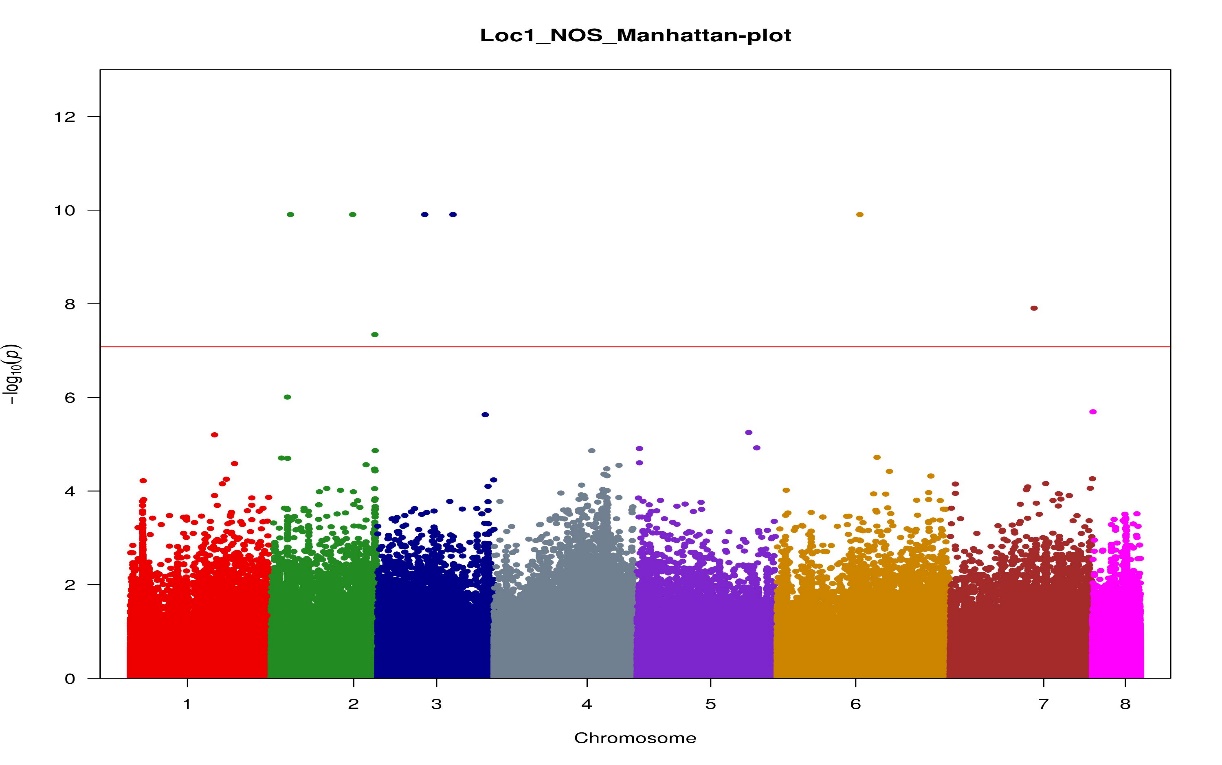


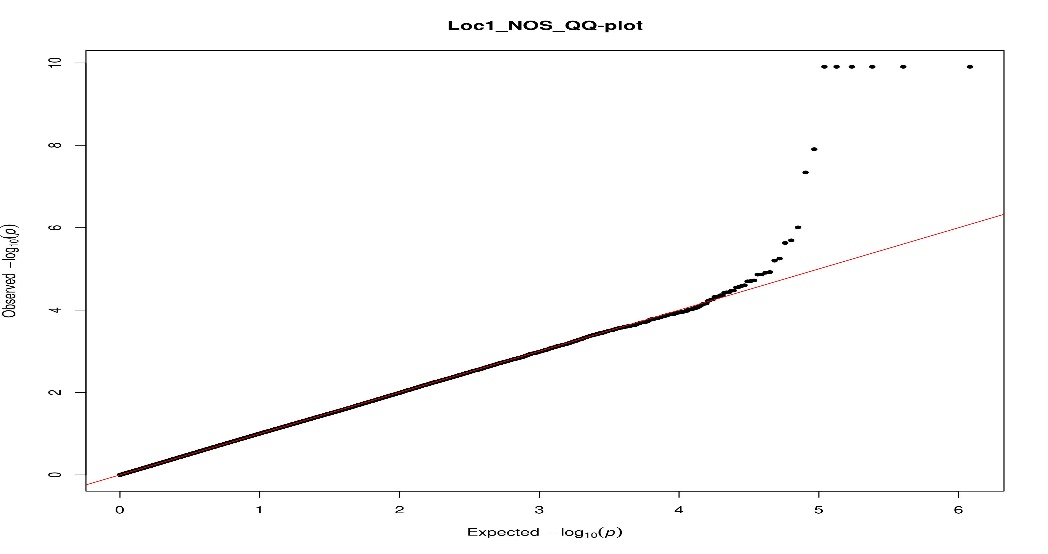


**Supplementary figure 2(d): Manhattan plot illustrating SNPs linked to number of seeds with their corresponding statistical significance represented by Q-Q plot for location 1 -FarmCPU model.**


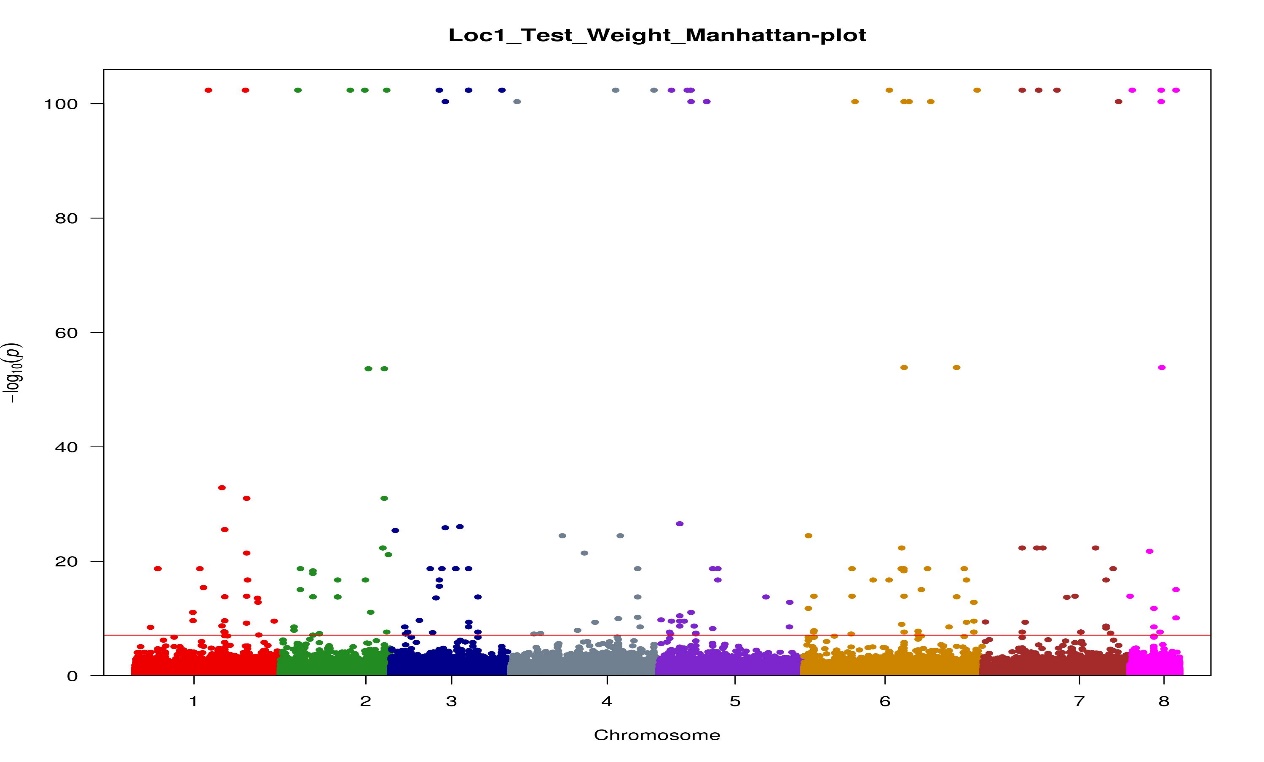


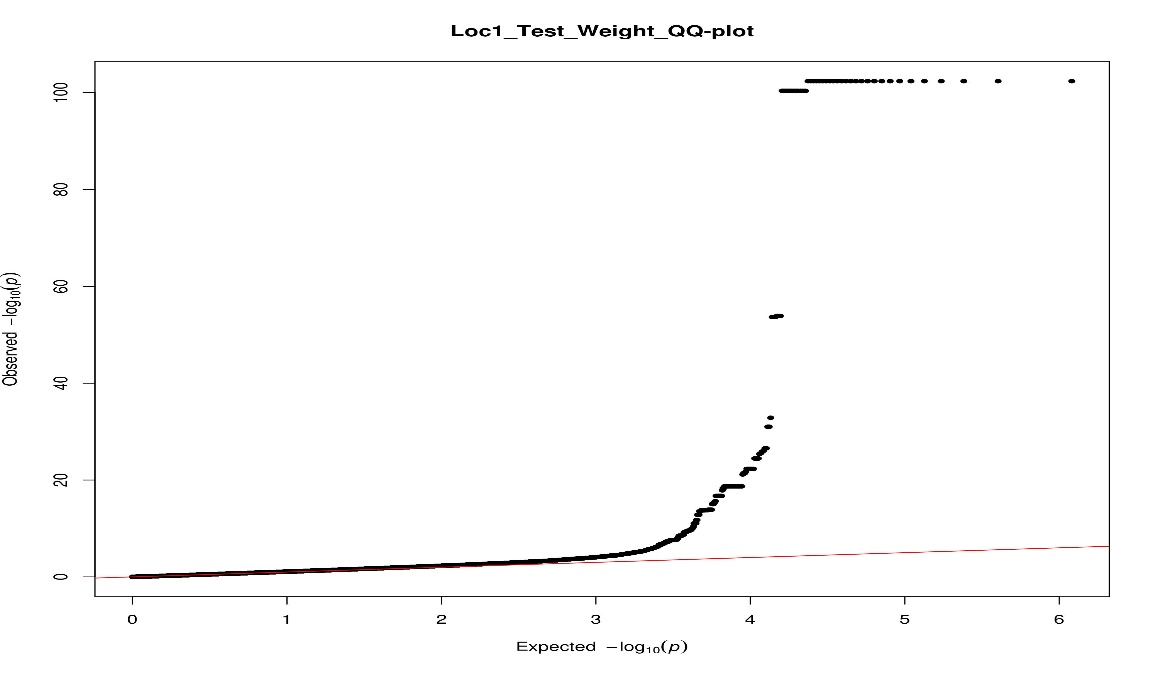


**Supplementary figure 2(e): Manhattan plot illustrating SNPs linked to test weight with their corresponding statistical significance represented by Q-Q plot for location 1 -FarmCPU model**


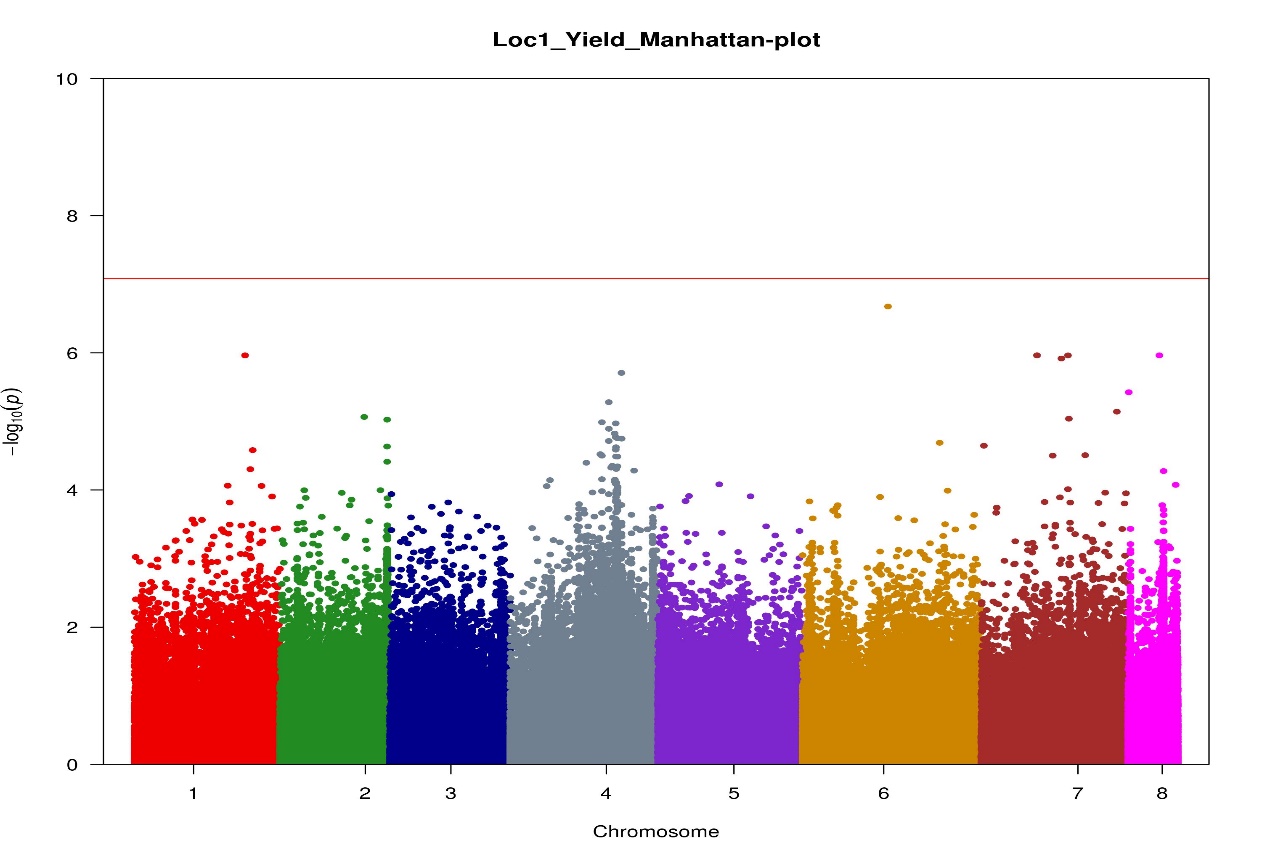


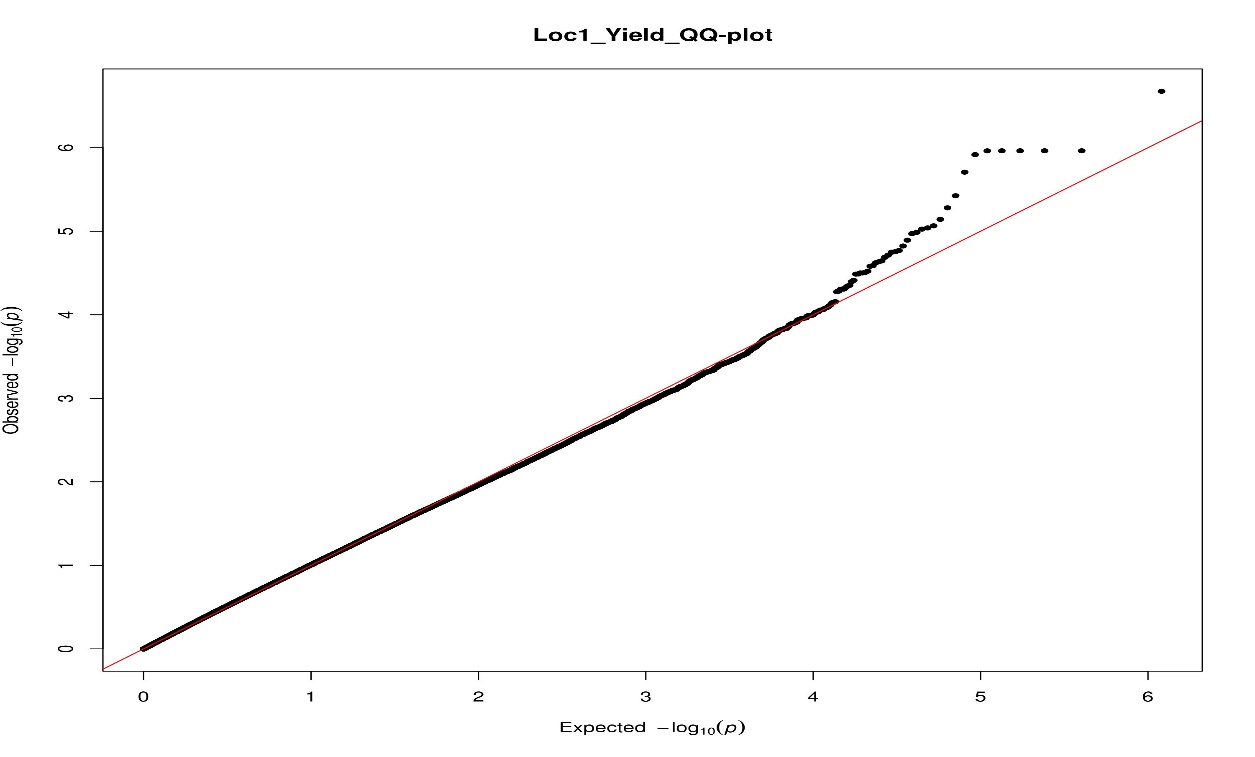


**Supplementary figure 2(f): Manhattan plot illustrating SNPs linked to yield with their corresponding statistical significance represented by Q-Q plot for location 1 -FarmCPU model**
