# Supplementary material for: Circ_0060927 regulates miR-331-3p/ERK/MAPK pathway reaction in non-small cell lung cancer through METTL14-driven methylation
Source: Front Oncol. 2025 Nov 13;15:1609215. doi: 10.3389/fonc.2025.1609215 (PMC12657173; doi:10.3389/fonc.2025.1609215)
Supplement: Supplementary file 2 [file Supplementaryfile2.docx]

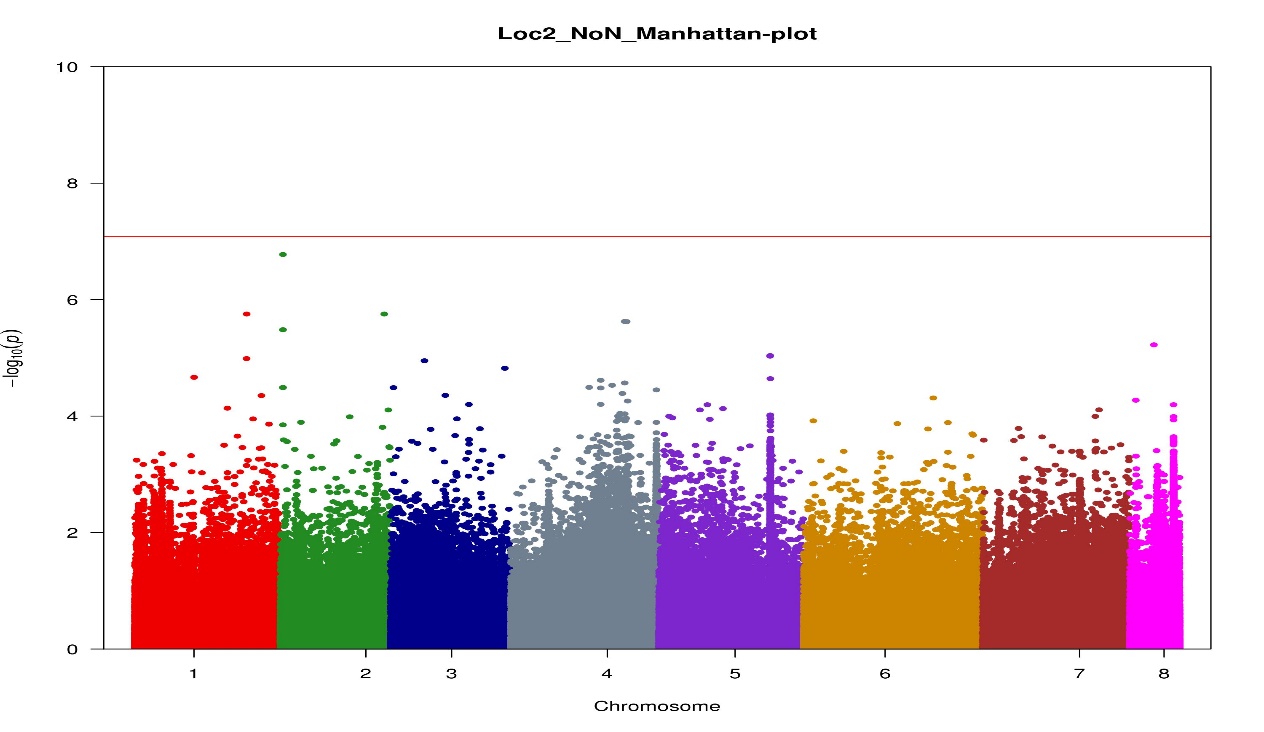


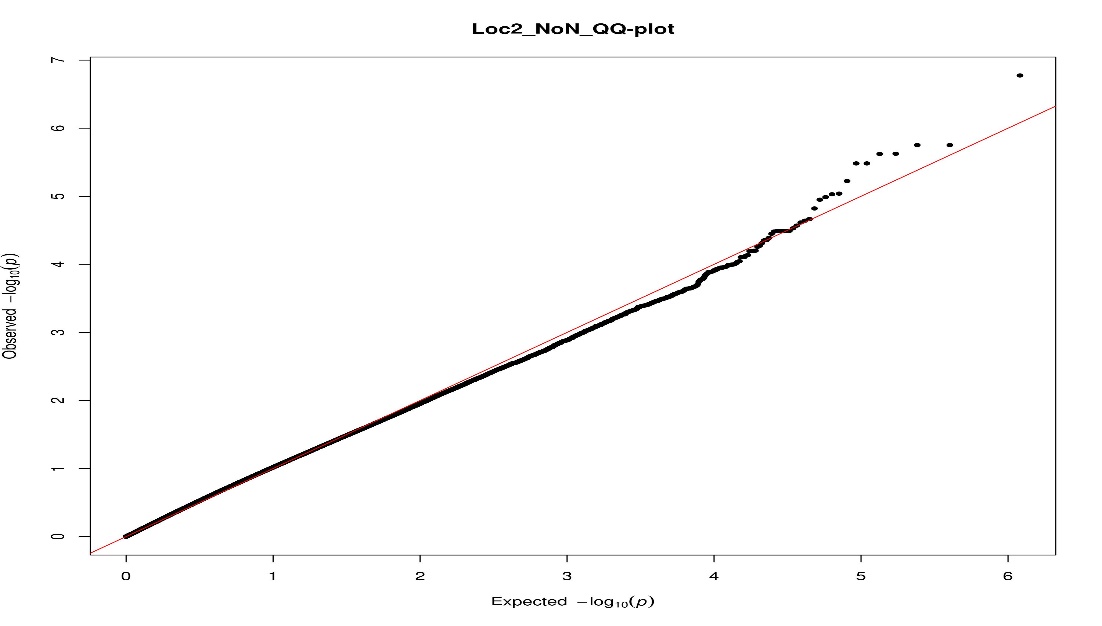


**Supplementary figure 3(a): Manhattan plot illustrating SNPs linked to number of nodules with their corresponding statistical significance represented by Q-Q plot for location 2 -Blink model**


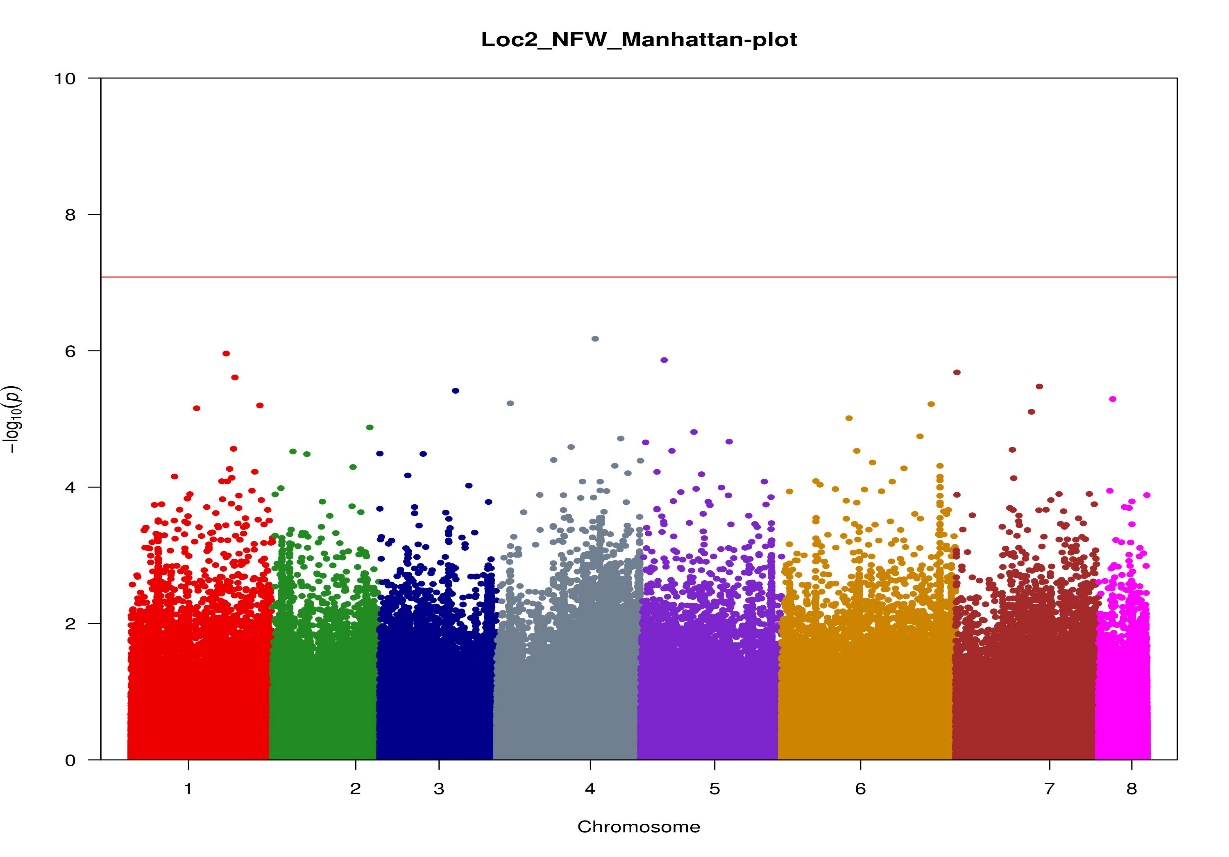


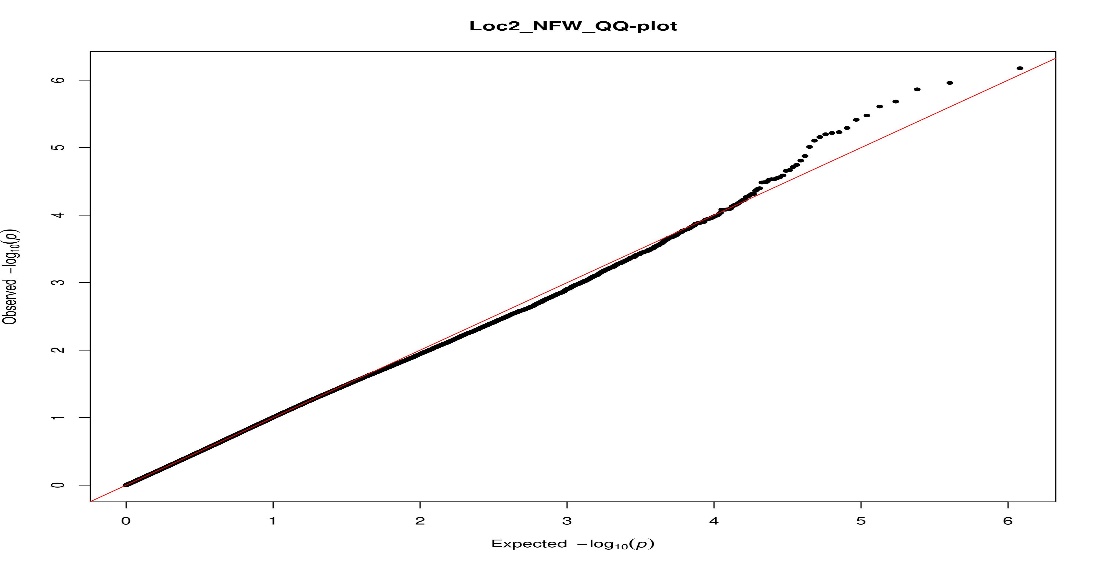


**Supplementary figure 3(b): Manhattan plot illustrating SNPs linked to nodule fresh weight with their corresponding statistical significance represented by Q-Q plot for location 2 -Blink model**


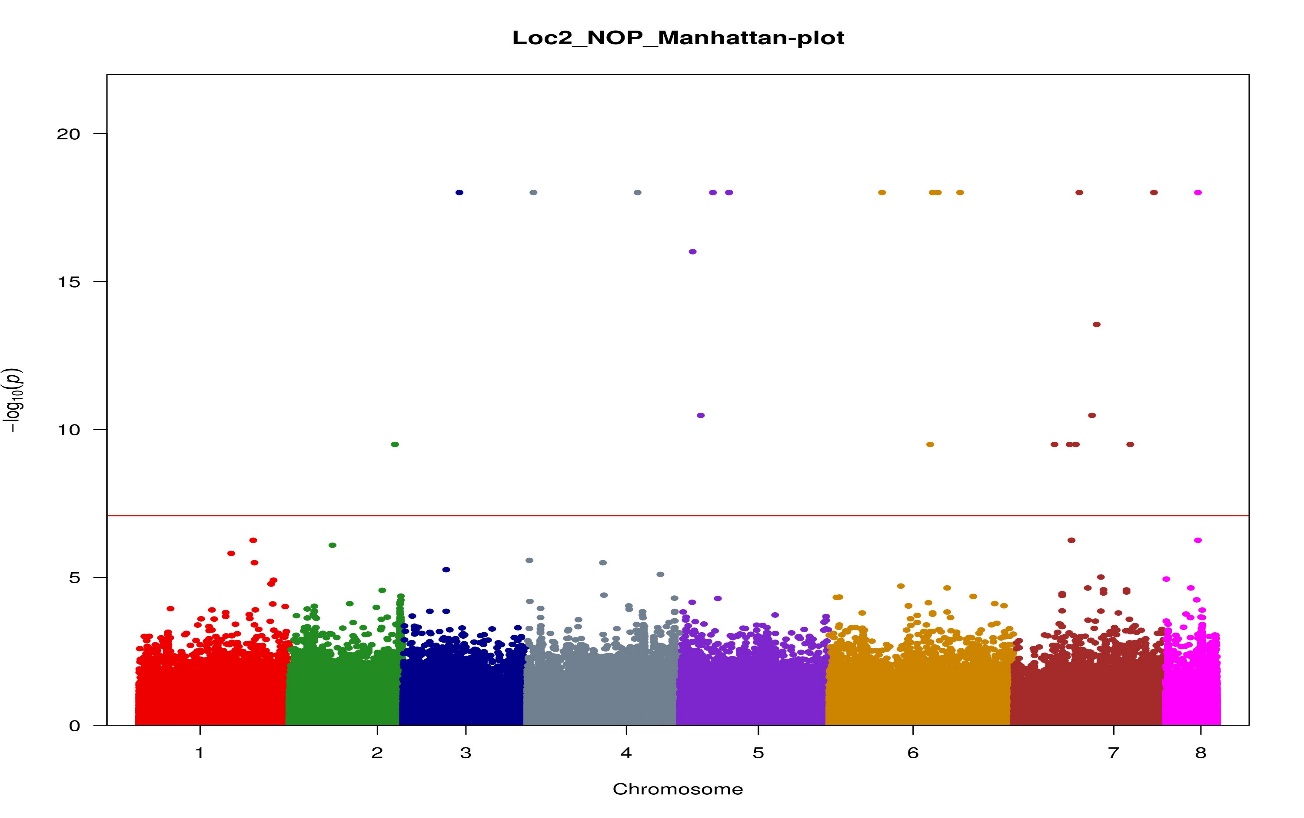


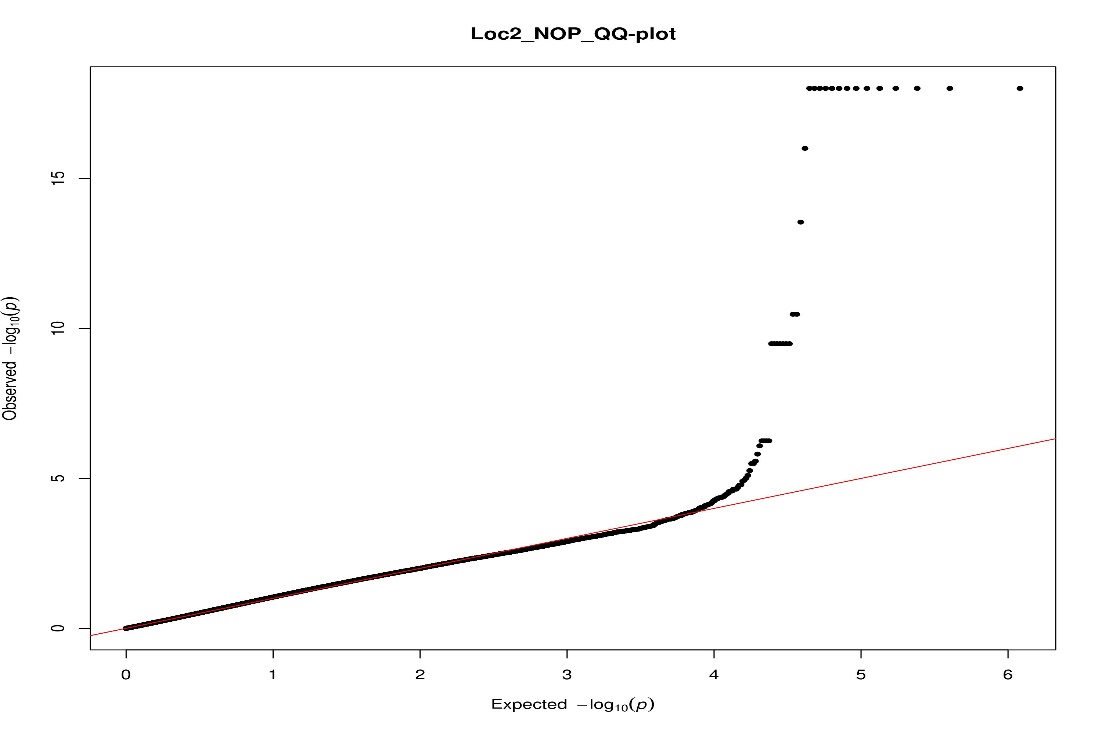


**Supplementary figure 3(c): Manhattan plot illustrating SNPs linked to number of pods with their corresponding statistical significance represented by Q-Q plot for location 2 -Blink model**


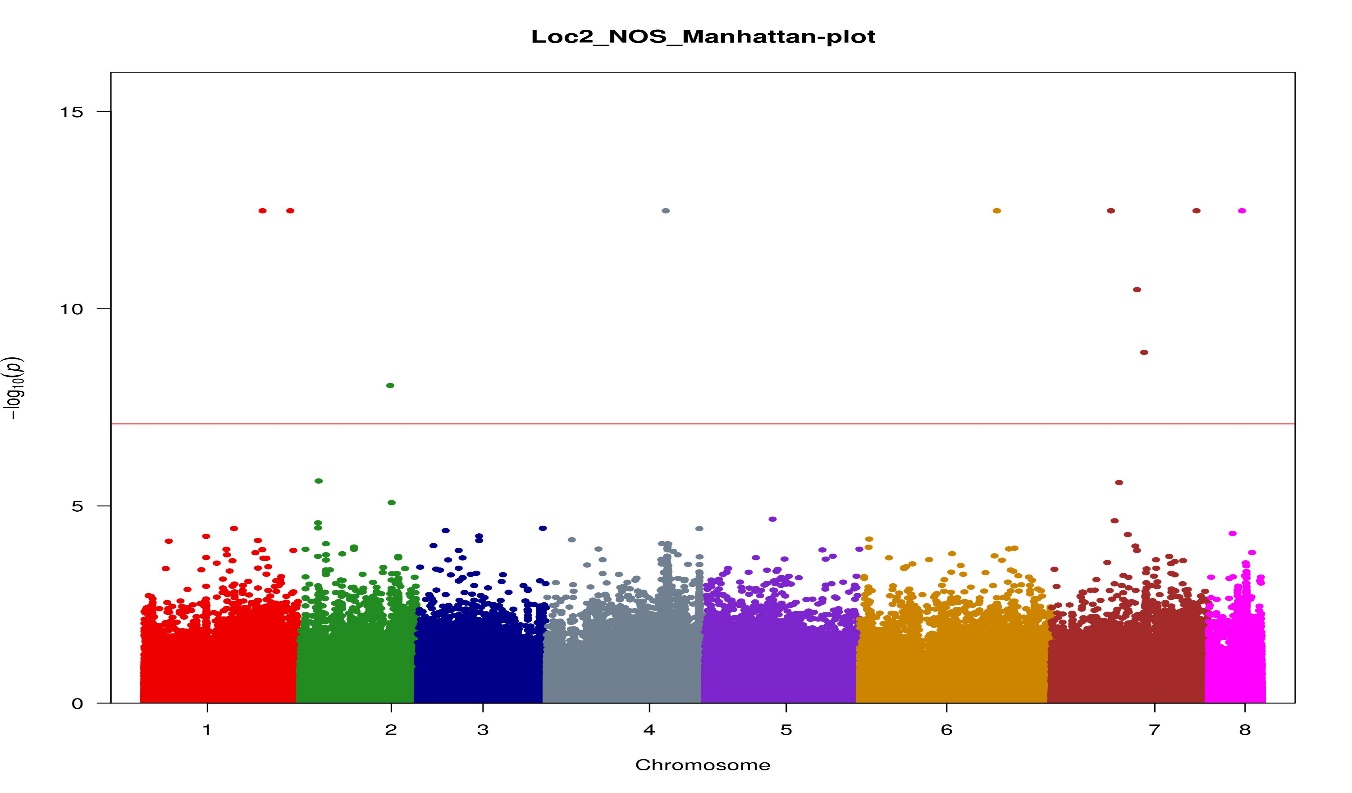


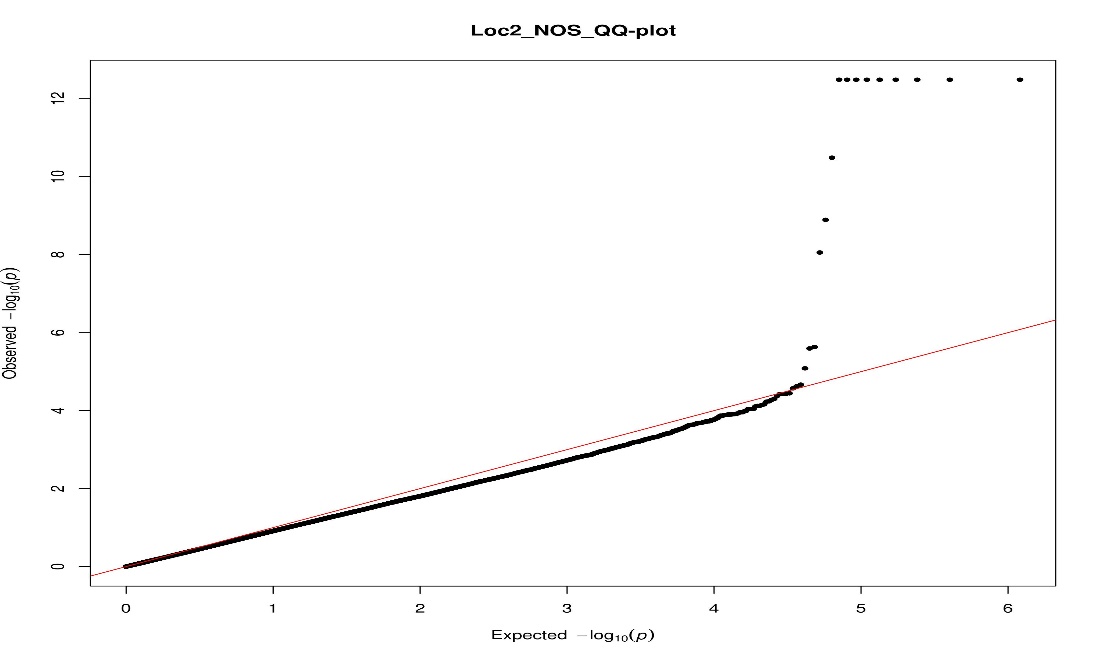


**Supplementary figure 3(d): Manhattan plot illustrating SNPs linked to number of seeds with their corresponding statistical significance represented by Q-Q plot for location 2 -Blink model**


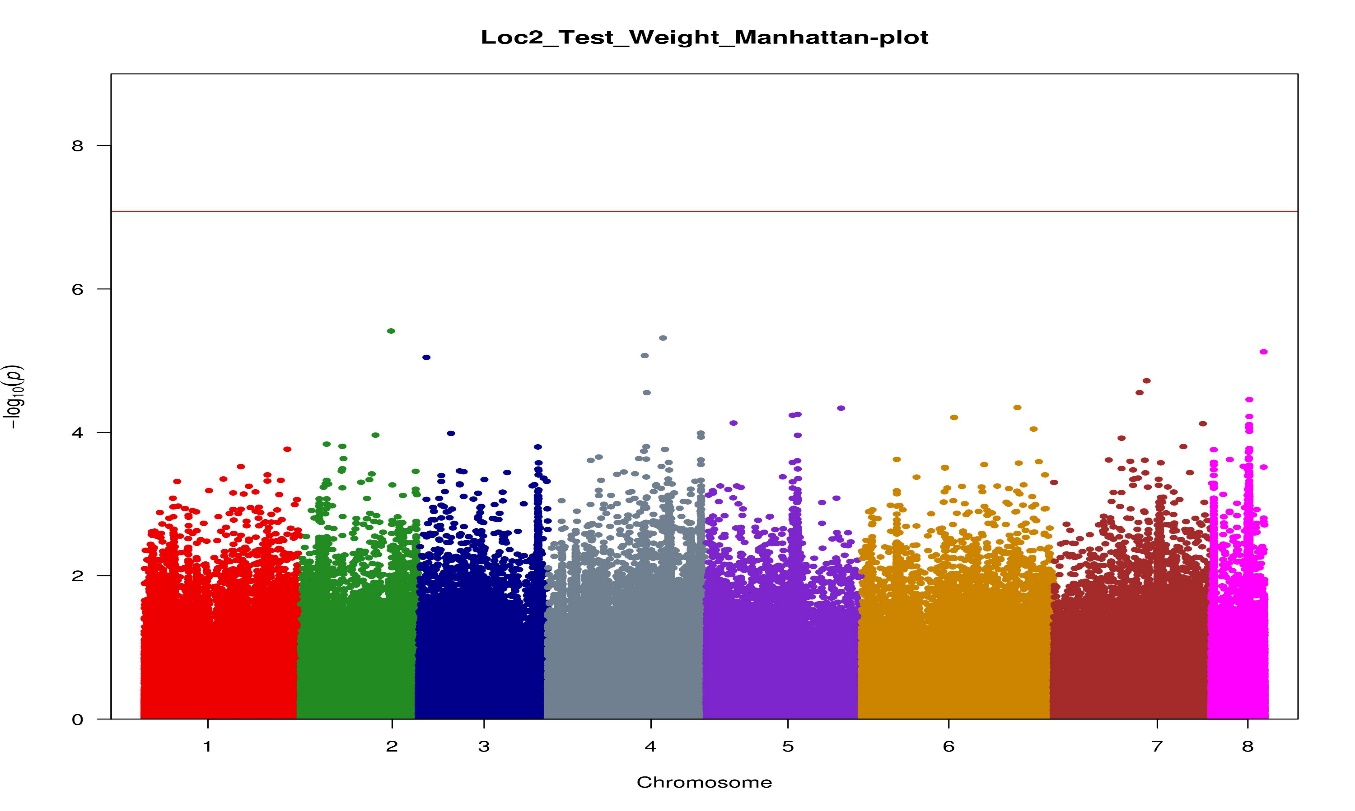


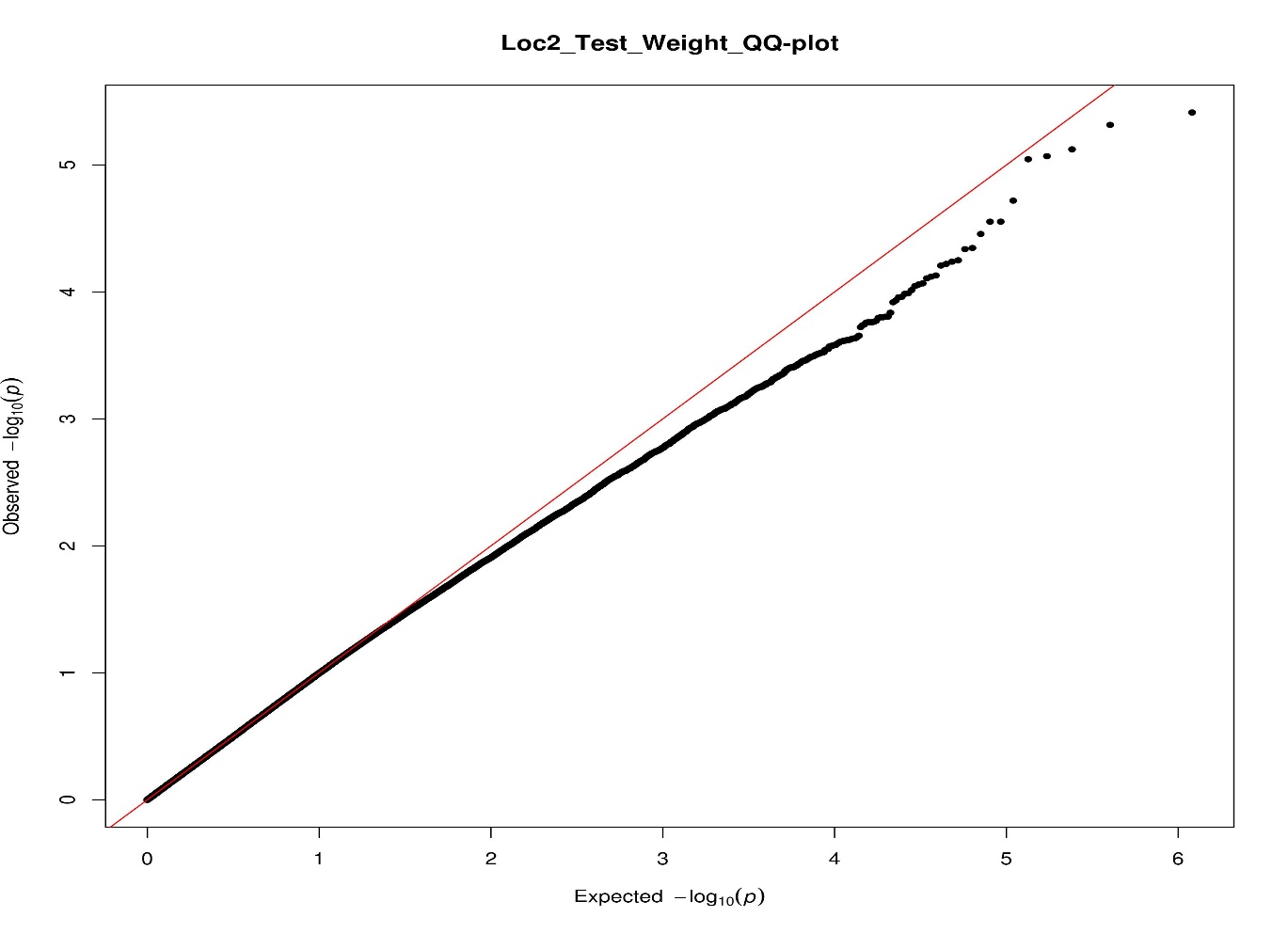


**Supplementary figure 3€: Manhattan plot illustrating SNPs linked to number of test weight location 2 -Blink model**


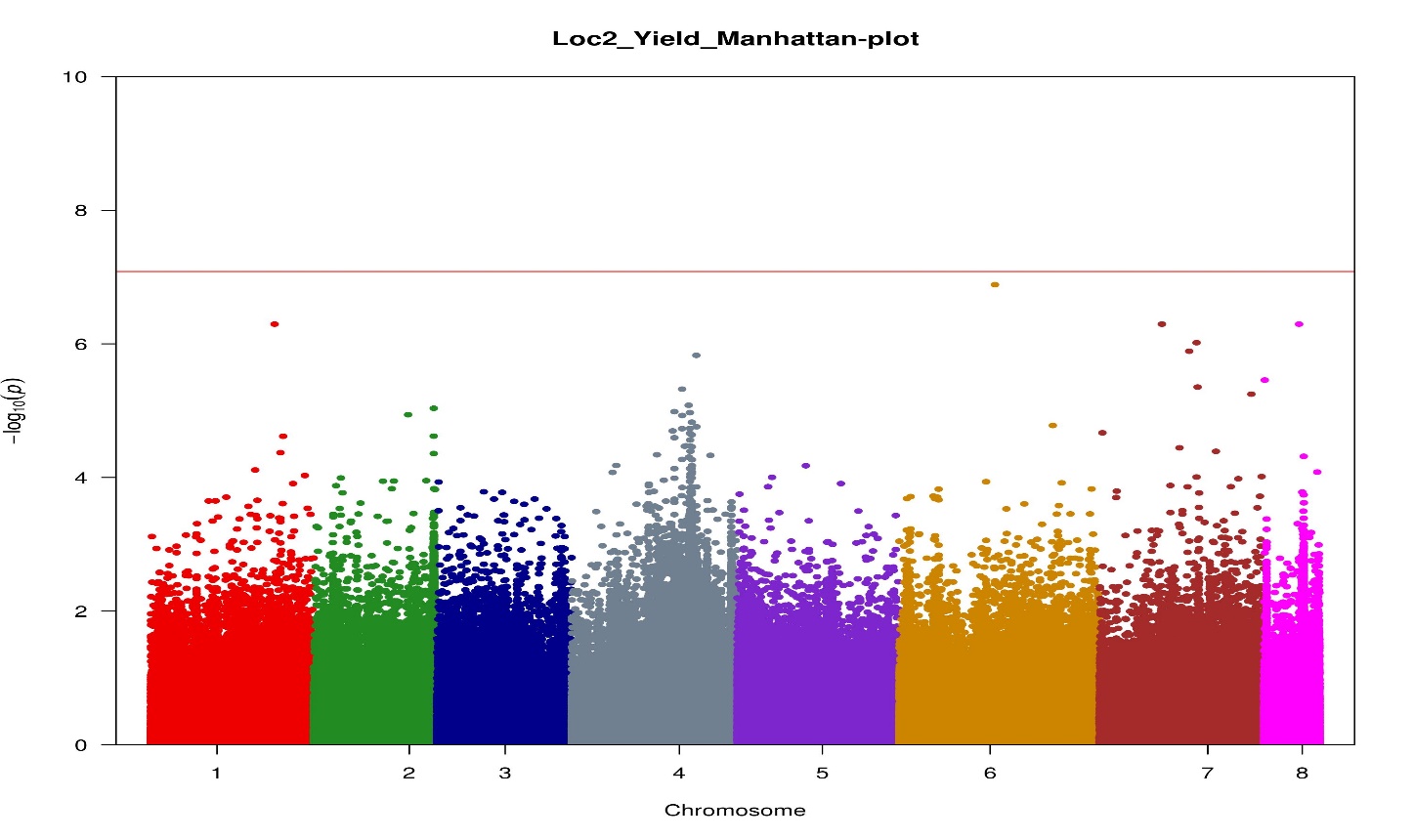


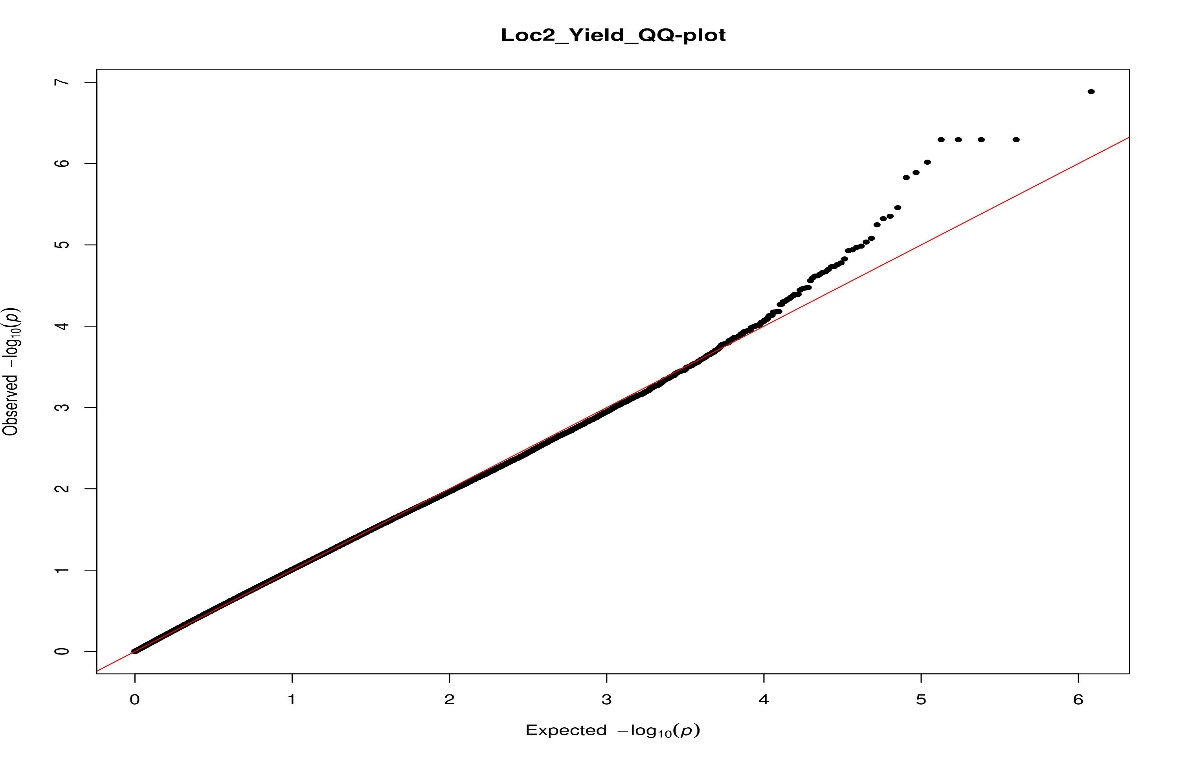


**Supplementary figure 3(f): Manhattan plot illustrating SNPs linked to yield with their corresponding statistical significance represented by Q-Q plot for location 2 -Blink model**


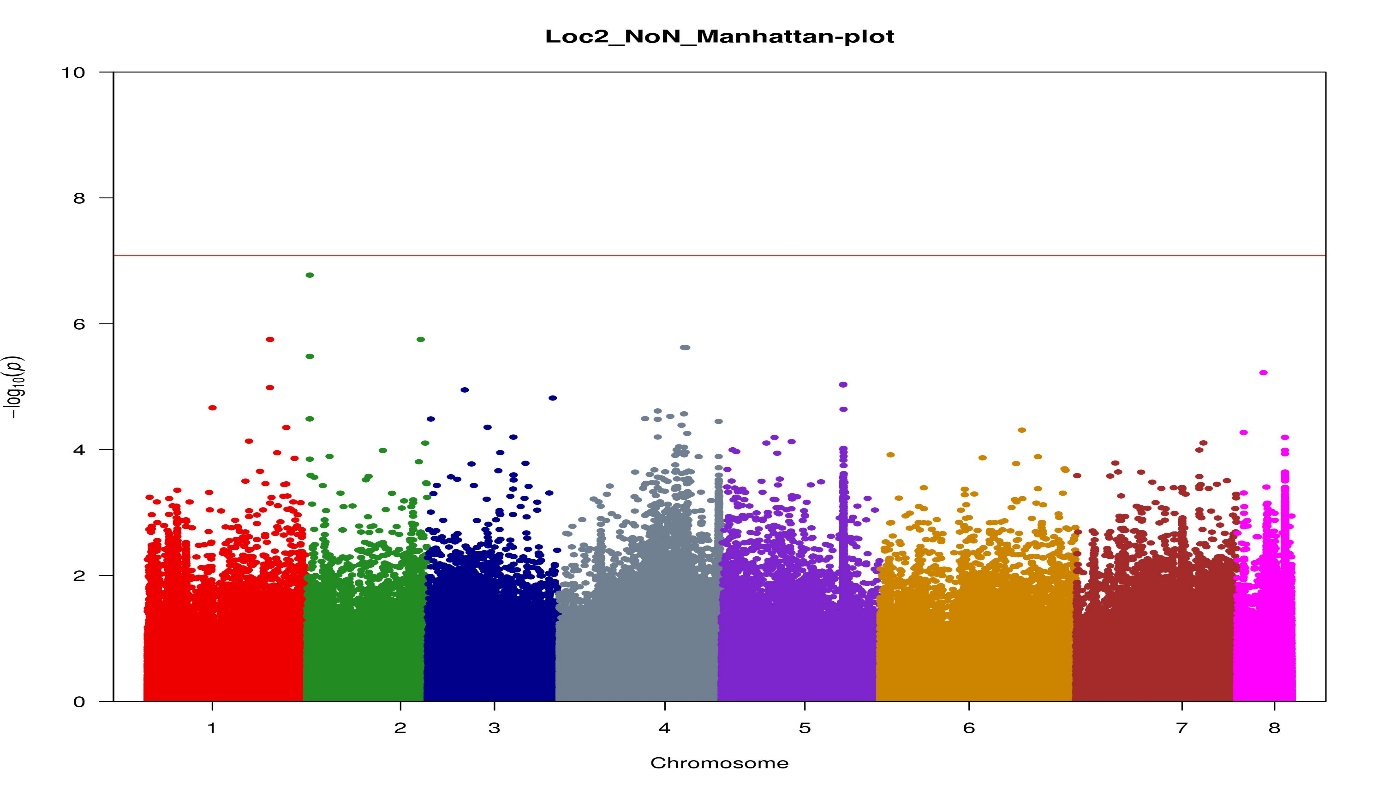


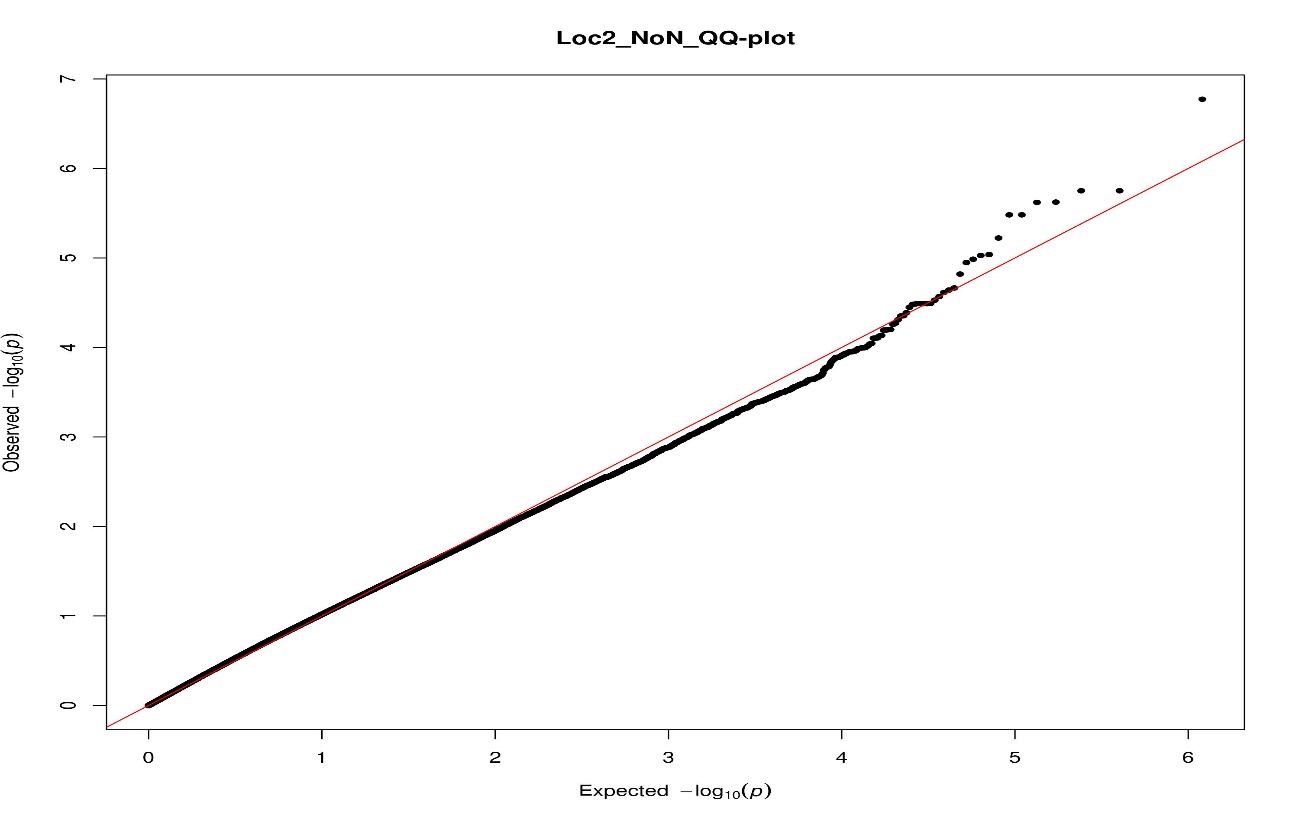


**Supplementary figure 4(a): Manhattan plot illustrating SNPs linked to number of nodules with their corresponding statistical significance represented by Q-Q plot for location 2 -FarmCPU model**


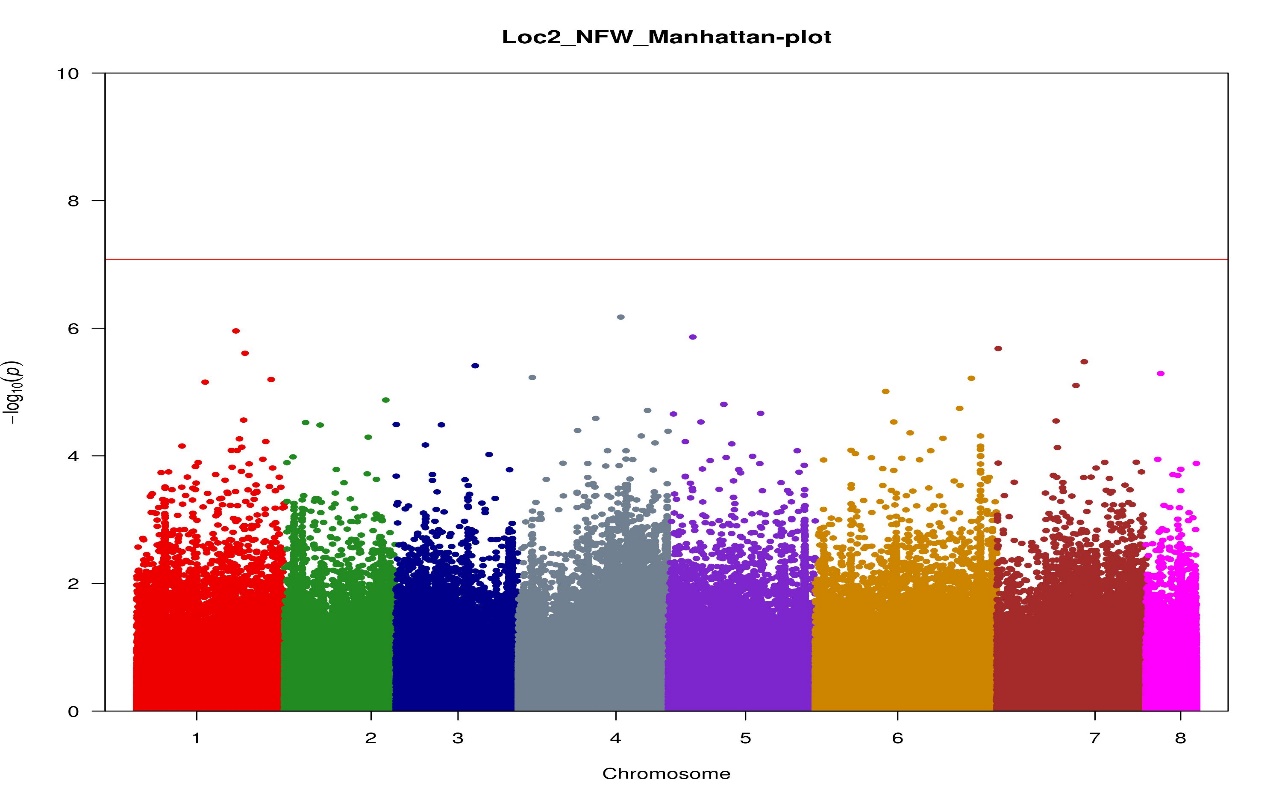


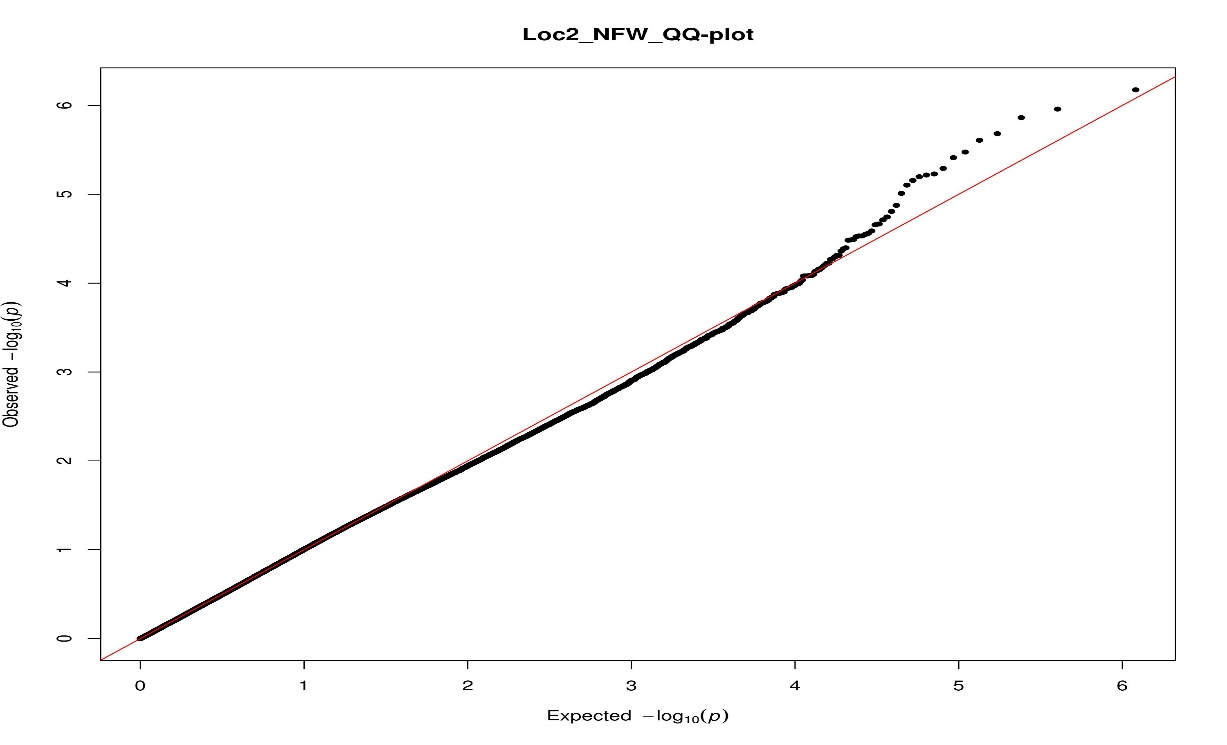


**Supplementary figure 4(b): Manhattan plot illustrating SNPs linked to nodule fresh weight with their corresponding statistical significance represented by Q-Q plot for location 2 -FarmCPU model**


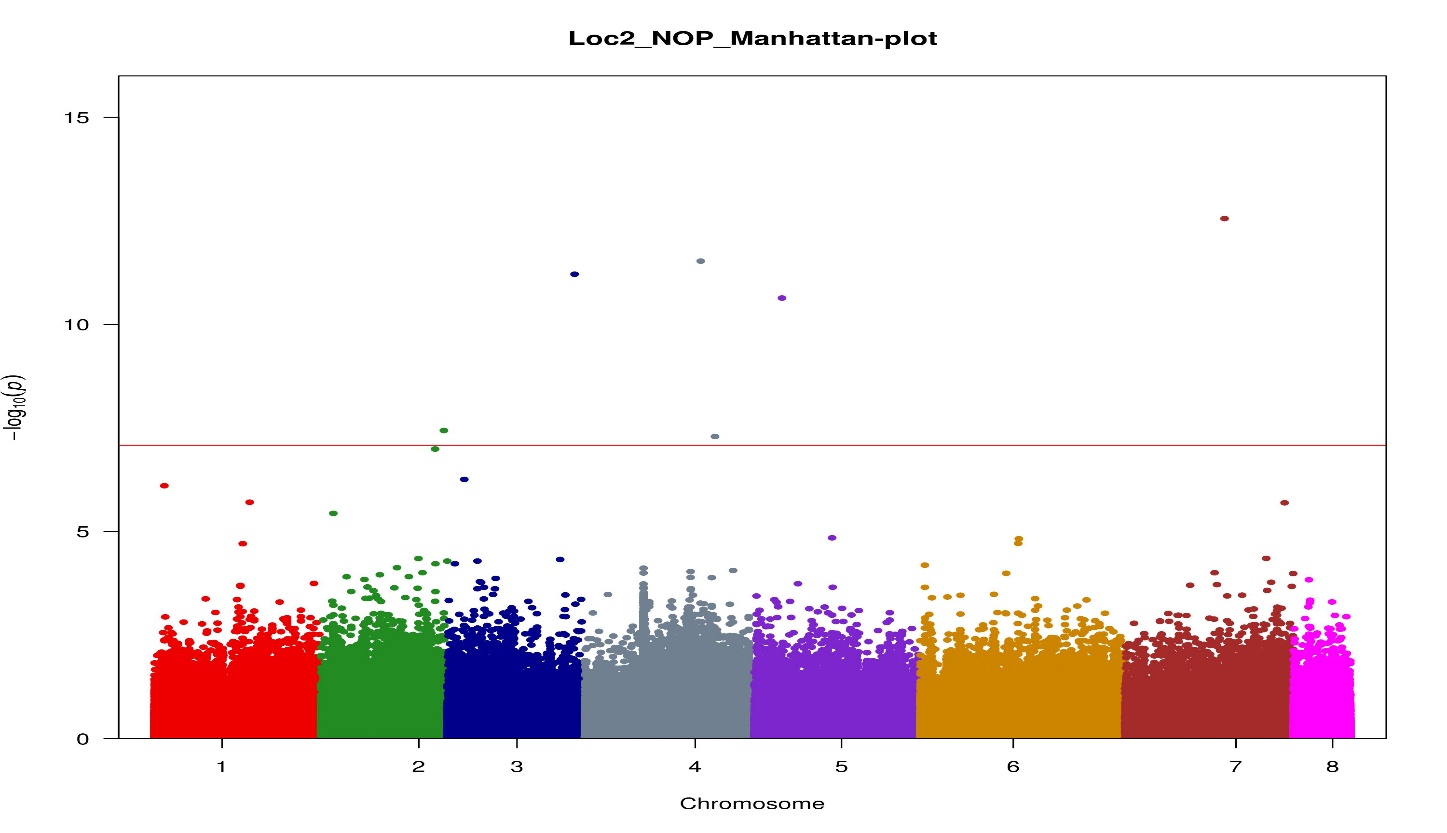


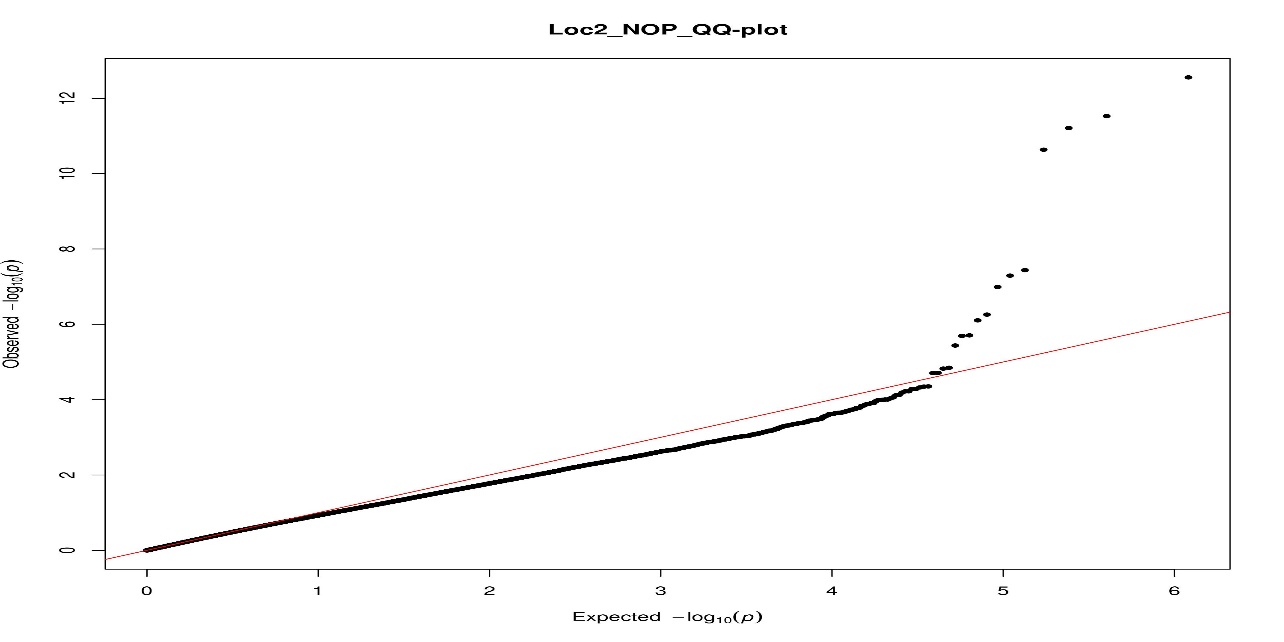


**Supplementary figure 4(c): Manhattan plot illustrating SNPs linked to number of pods with their corresponding statistical significance represented by Q-Q plot for location 2 -FarmCPU model**


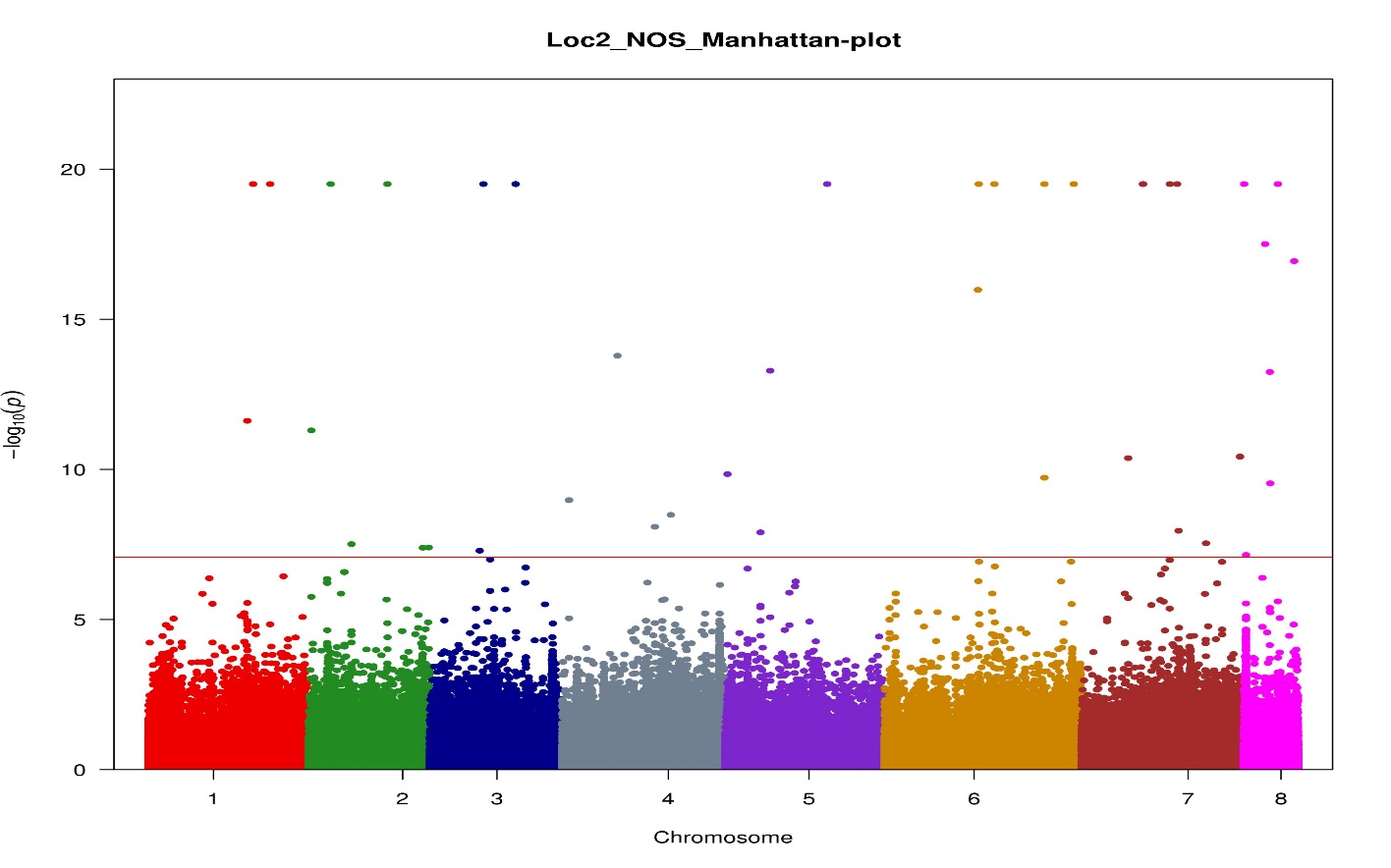


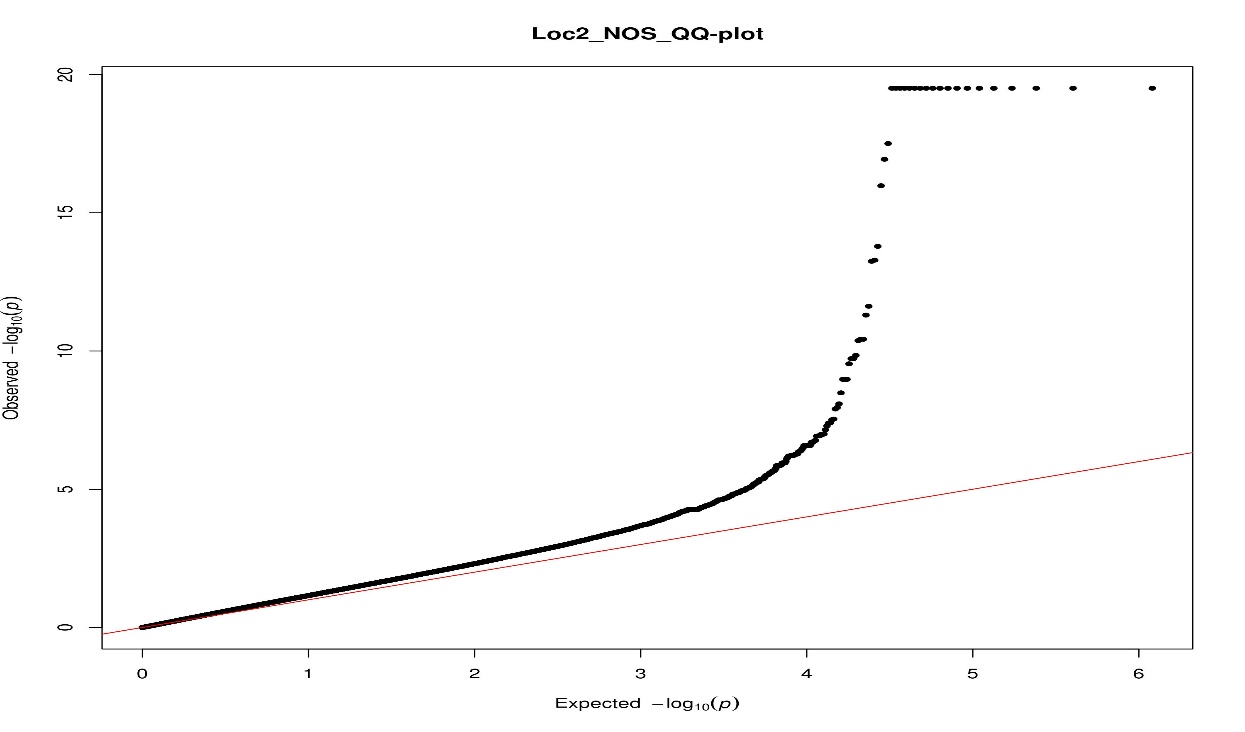


**Supplementary figure 4(d): Manhattan plot illustrating SNPs linked to number of seeds with their corresponding statistical significance represented by Q-Q plot for location 2 -FarmCPU model**


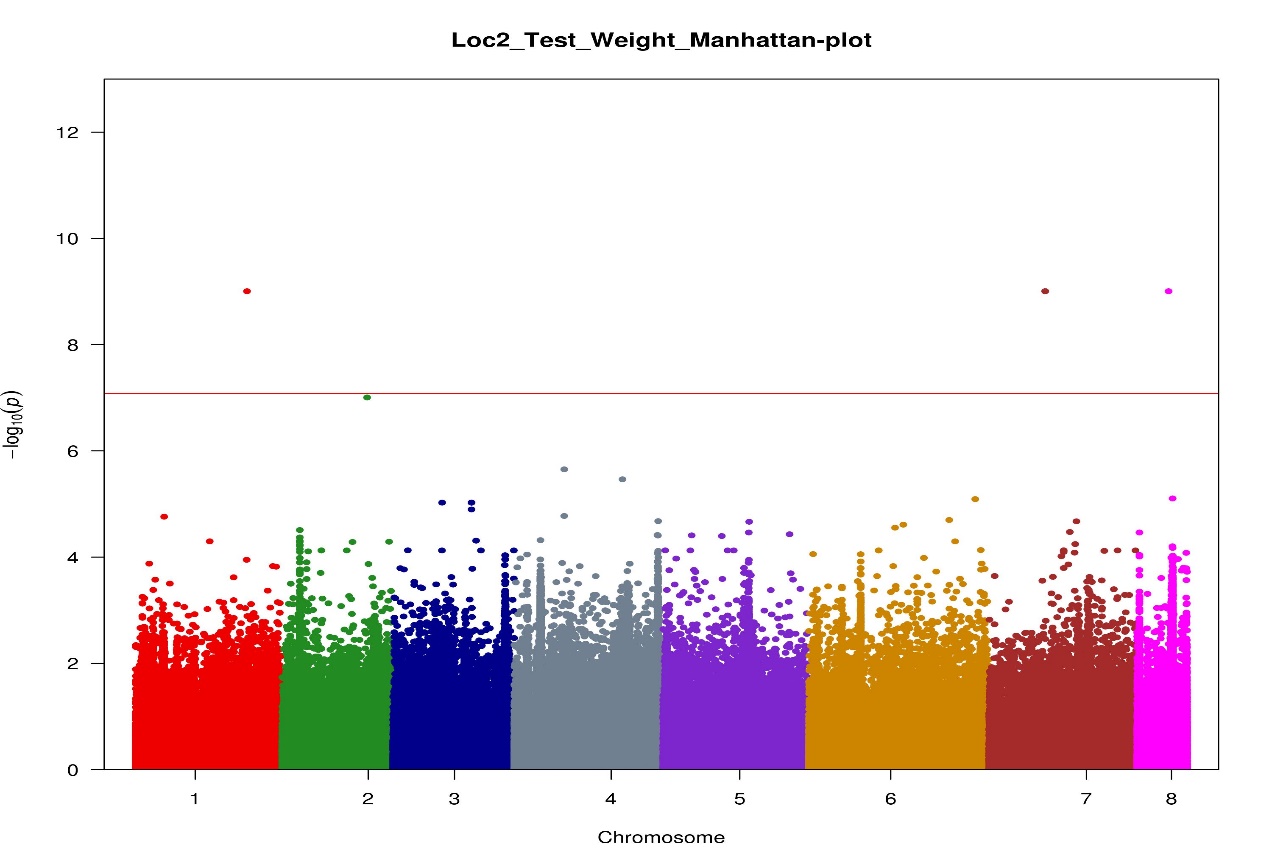


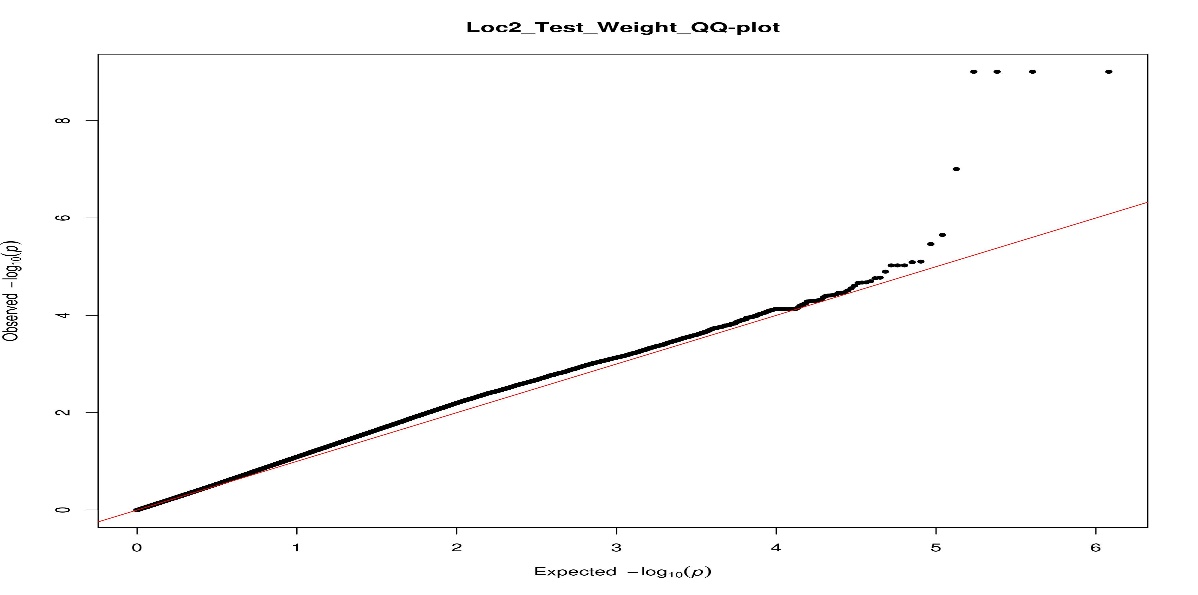


**Supplementary figure 4€: Manhattan plot illustrating SNPs linked to test weight with their corresponding statistical significance represented by Q-Q plot for location 2 -FarmCPU model**


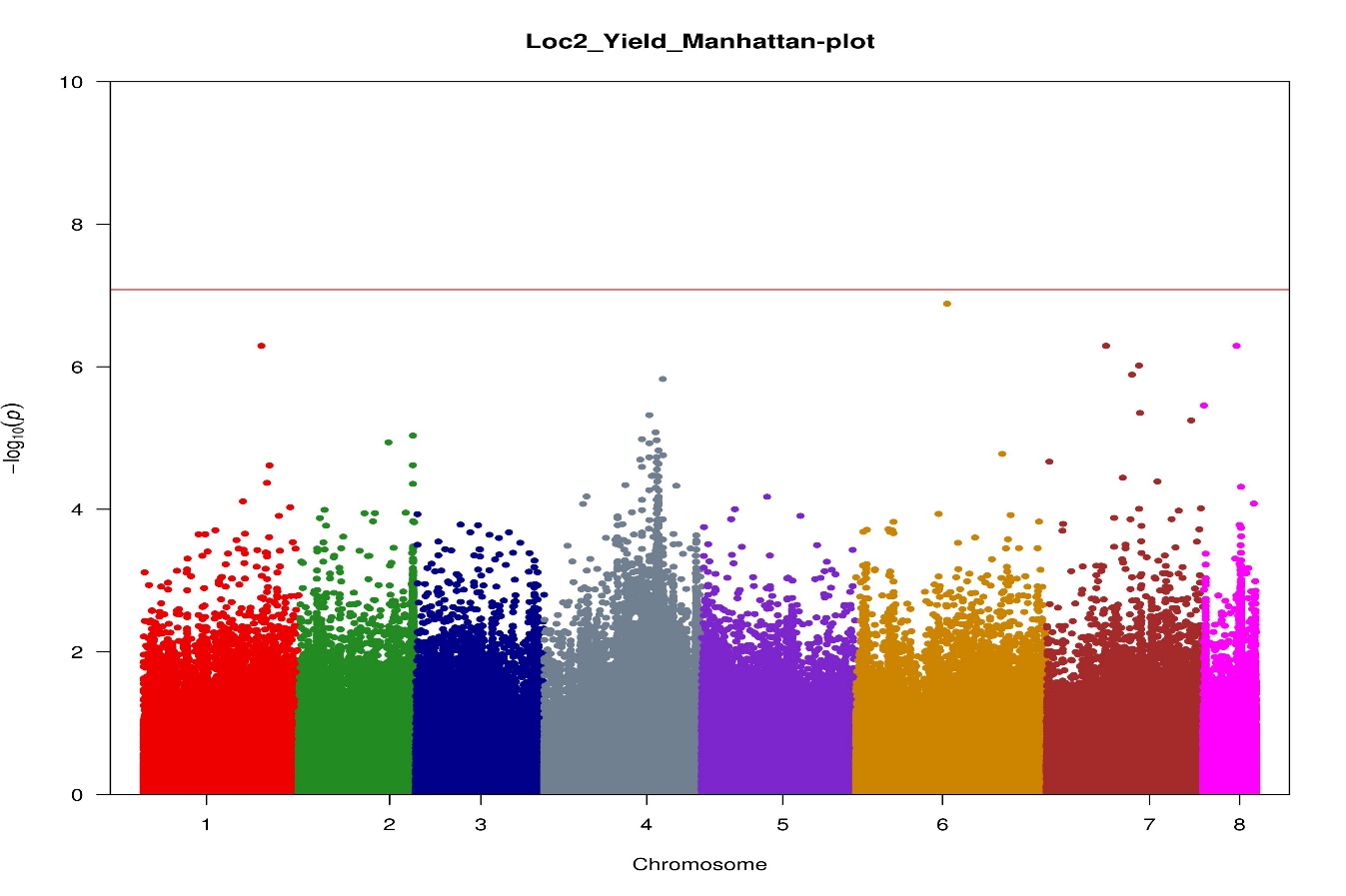


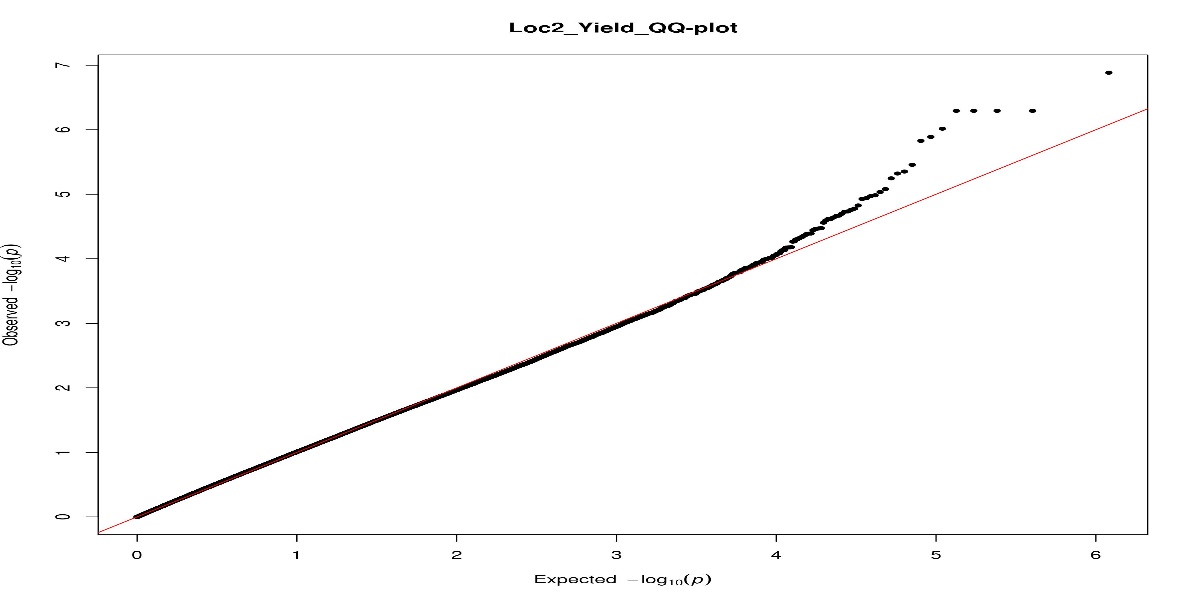


**Supplementary figure 4(f): Manhattan plot illustrating SNPs linked to number of seeds with their corresponding statistical significance represented by Q-Q plot for location 2 -FarmCPU model**
