# Supplementary material for: Circ_0060927 regulates miR-331-3p/ERK/MAPK pathway reaction in non-small cell lung cancer through METTL14-driven methylation
Source: Front Oncol. 2025 Nov 13;15:1609215. doi: 10.3389/fonc.2025.1609215 (PMC12657173; doi:10.3389/fonc.2025.1609215)
Supplement: Supplementary file 3 [file Supplementaryfile3.docx]

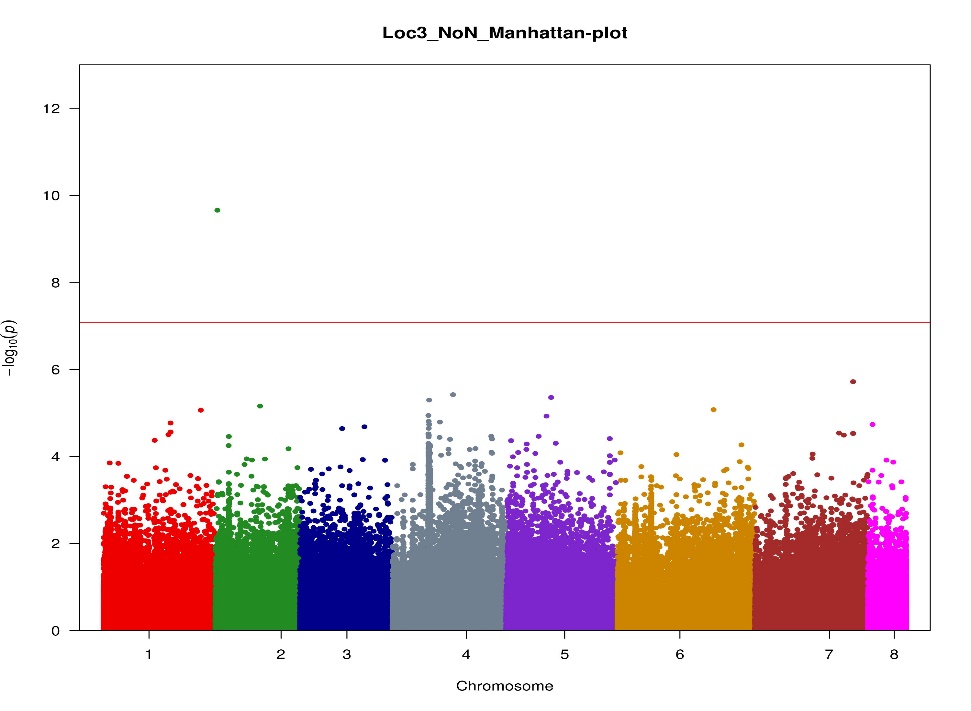


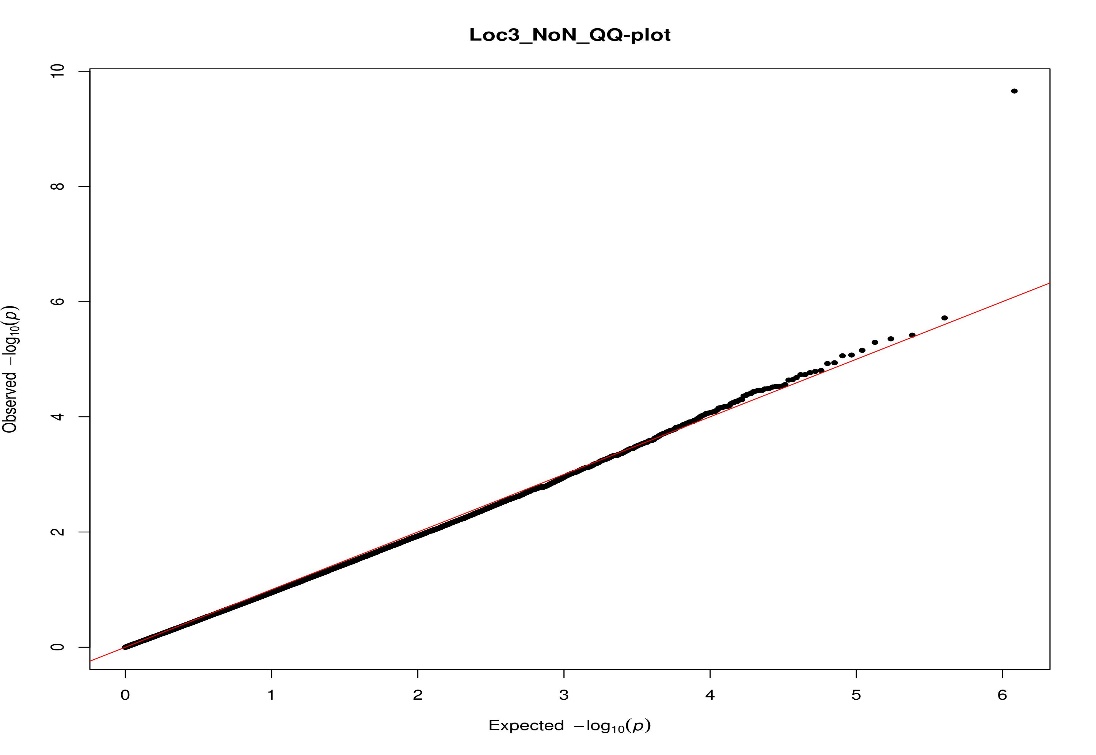


**Supplementary figure 5(a)**: Manhattan plot illustrating SNPs linked to number of nodules with their corresponding statistical significance represented by Q-Q plot for location 3 -Blink model


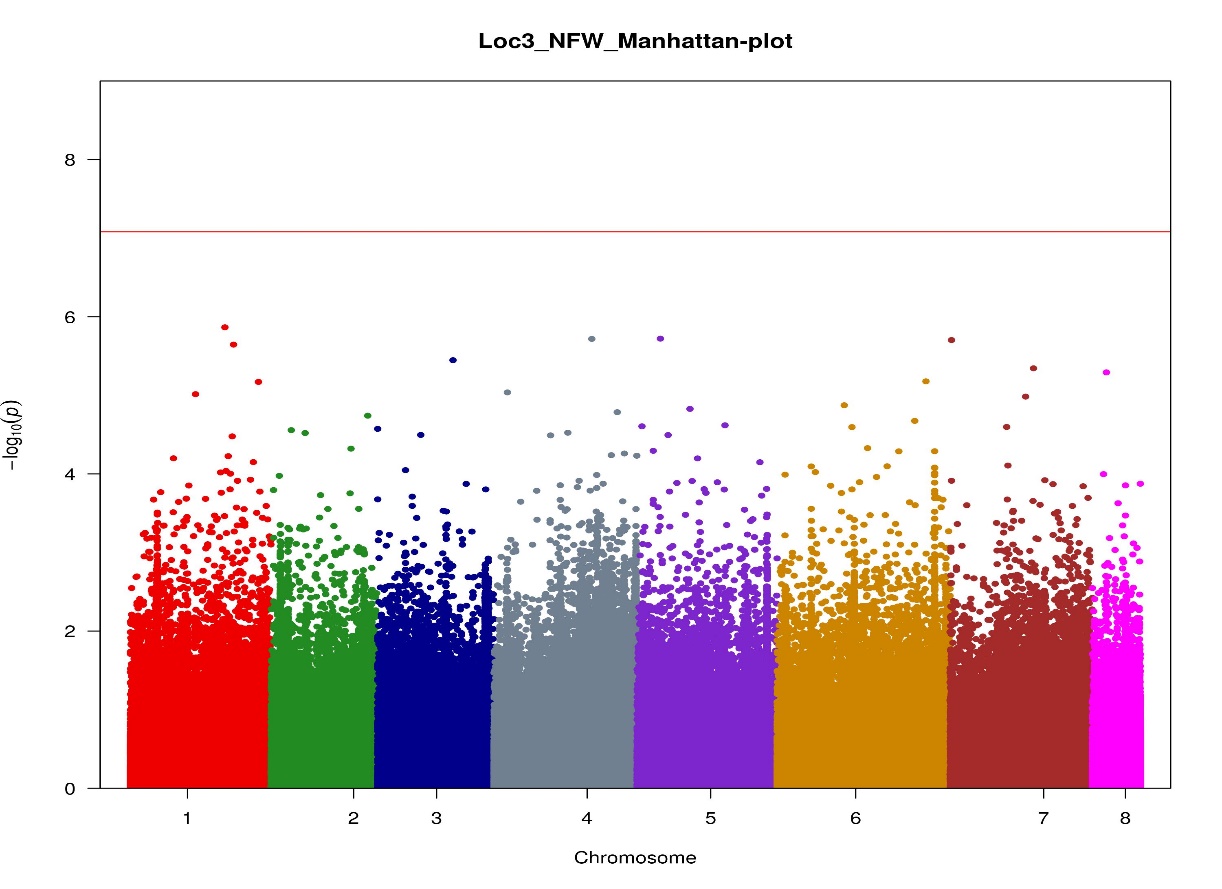


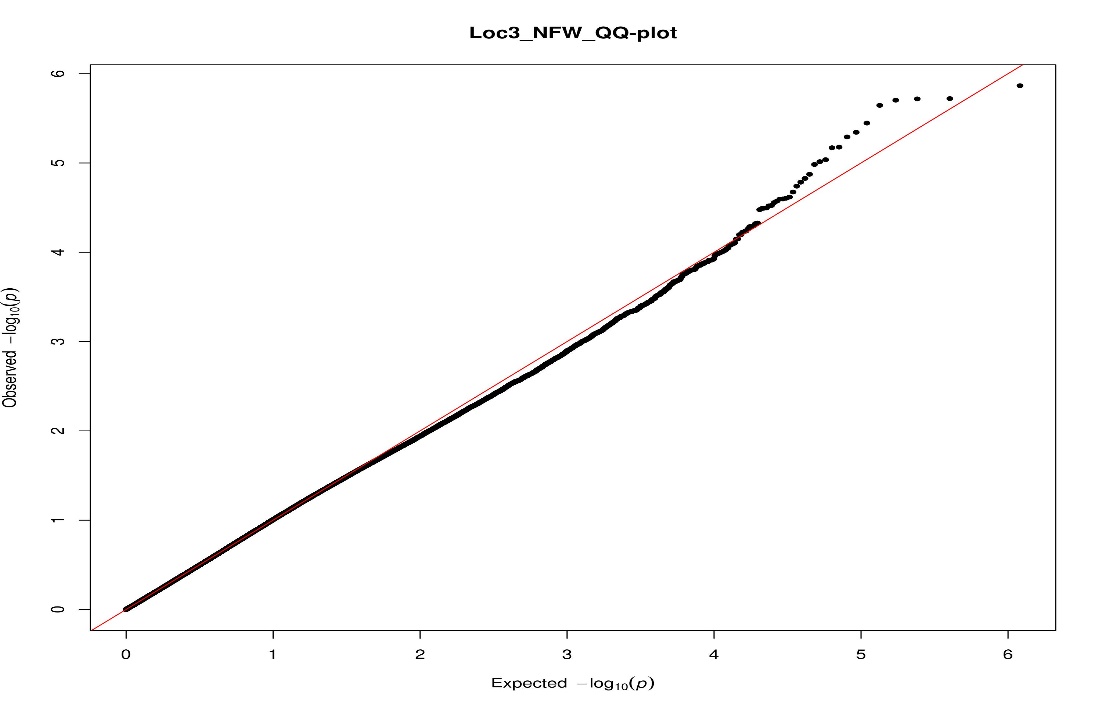


**Supplementary figure 5(b)**: Manhattan plot illustrating SNPs linked to nodule fresh weight with their corresponding statistical significance represented by Q-Q plot for location 3 -Blink model.


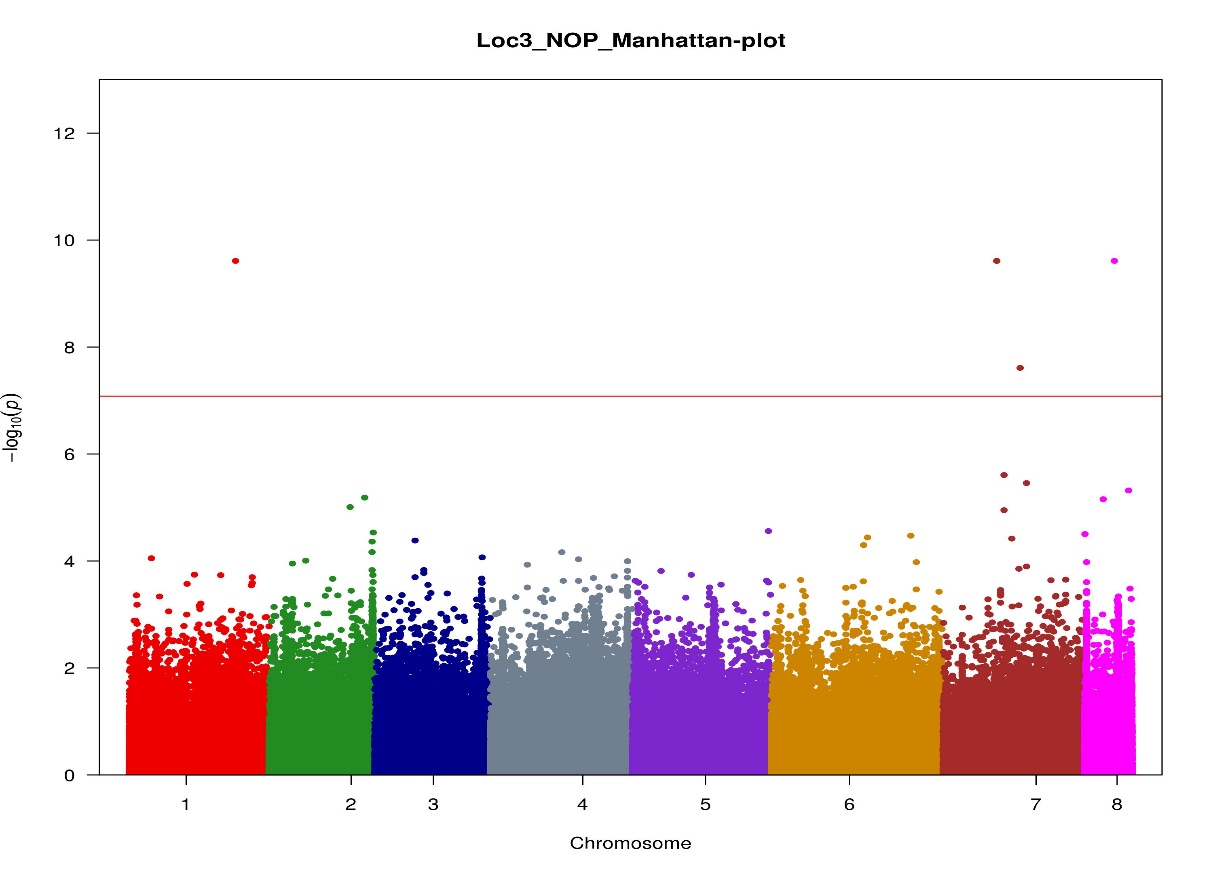


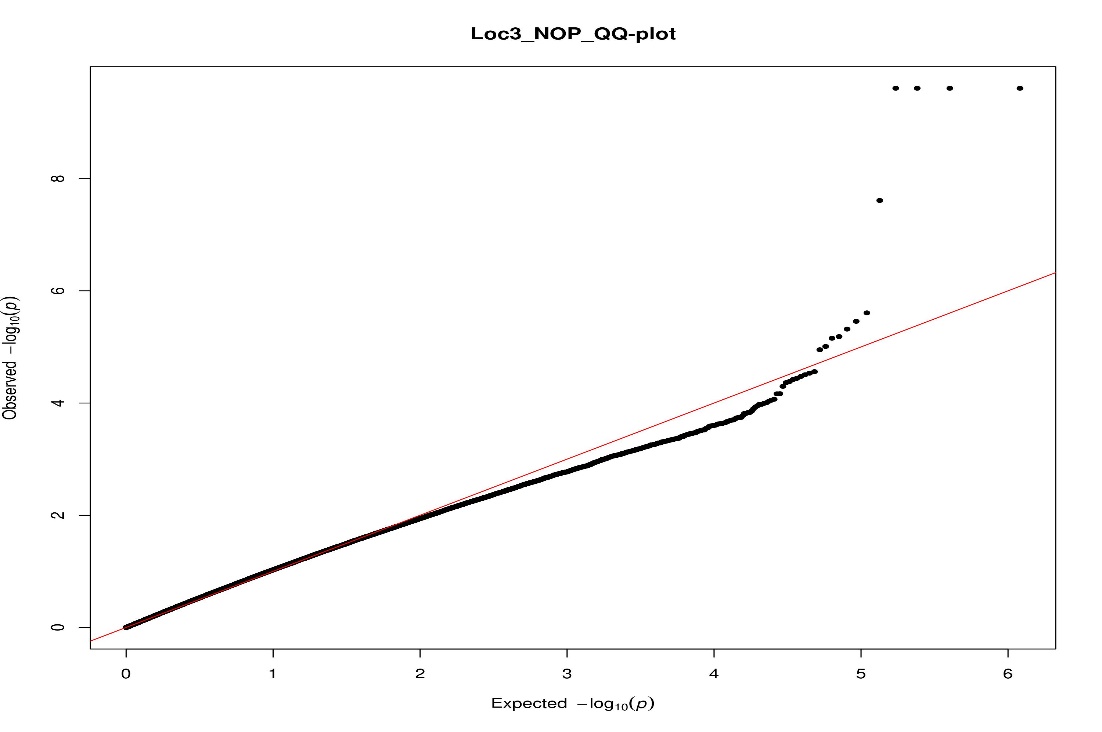


**Supplementary figure 5(c)**: Manhattan plot illustrating SNPs linked to number of pods with their corresponding statistical significance represented by Q-Q plot for location 3 -Blink model


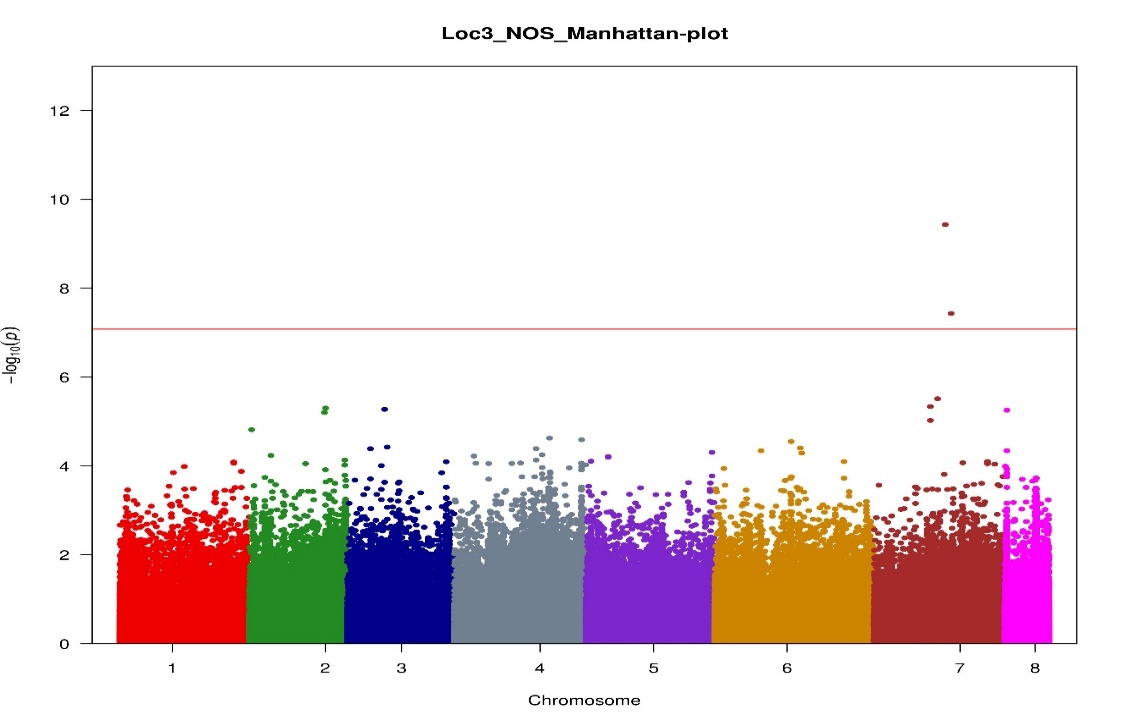


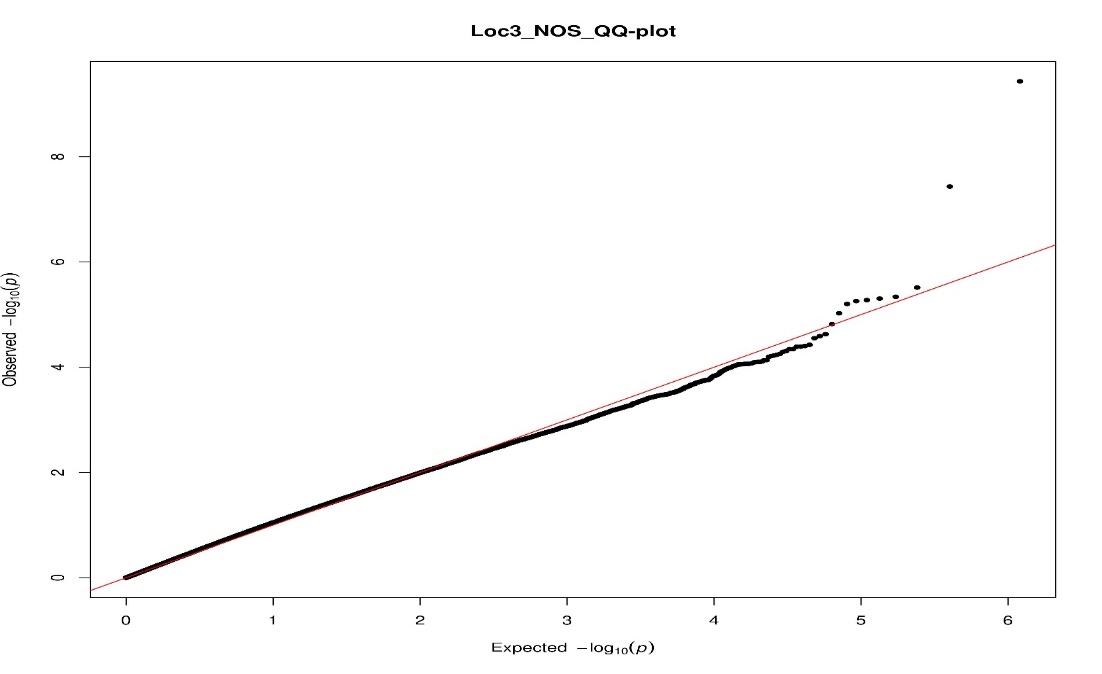


**Supplementary figure 5(d)**: Manhattan plot illustrating SNPs linked to number of seeds with their corresponding statistical significance represented by Q-Q plot for location 3 -Blink model


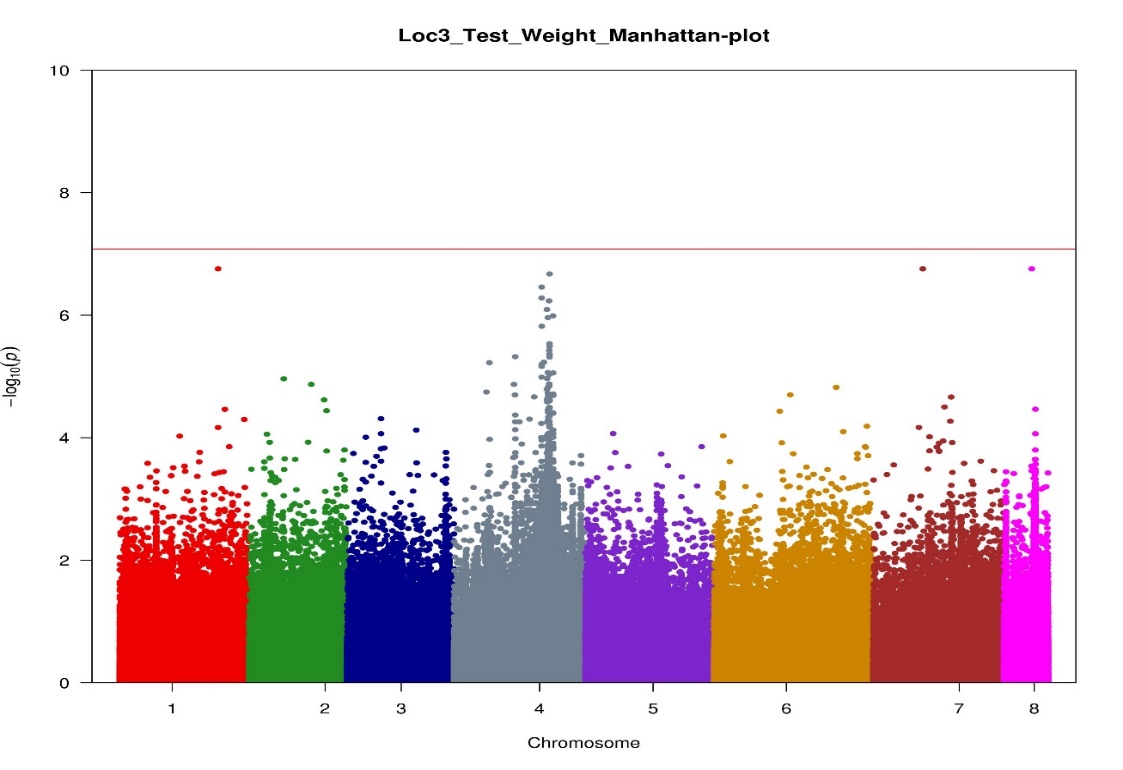


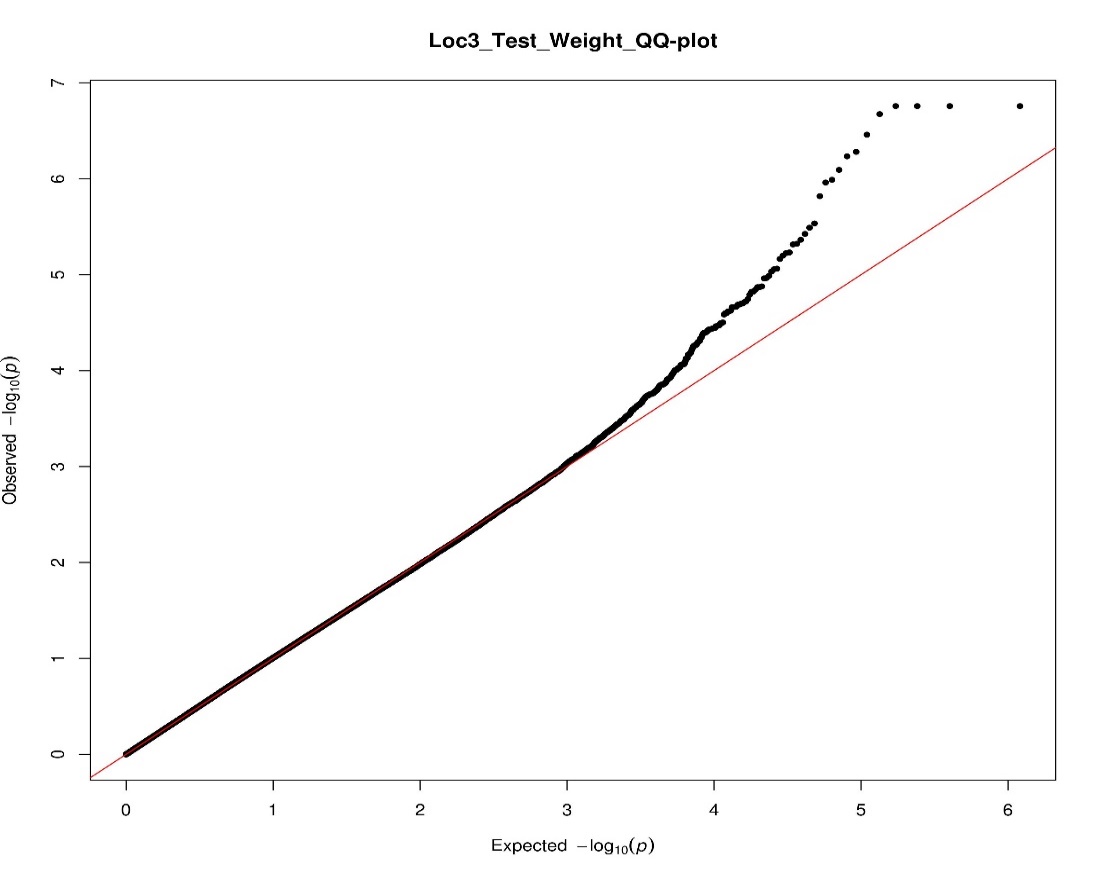


**Supplementary figure 5€**: Manhattan plot illustrating SNPs linked to test weight with their corresponding statistical significance represented by Q-Q plot for location 3 -Blink model


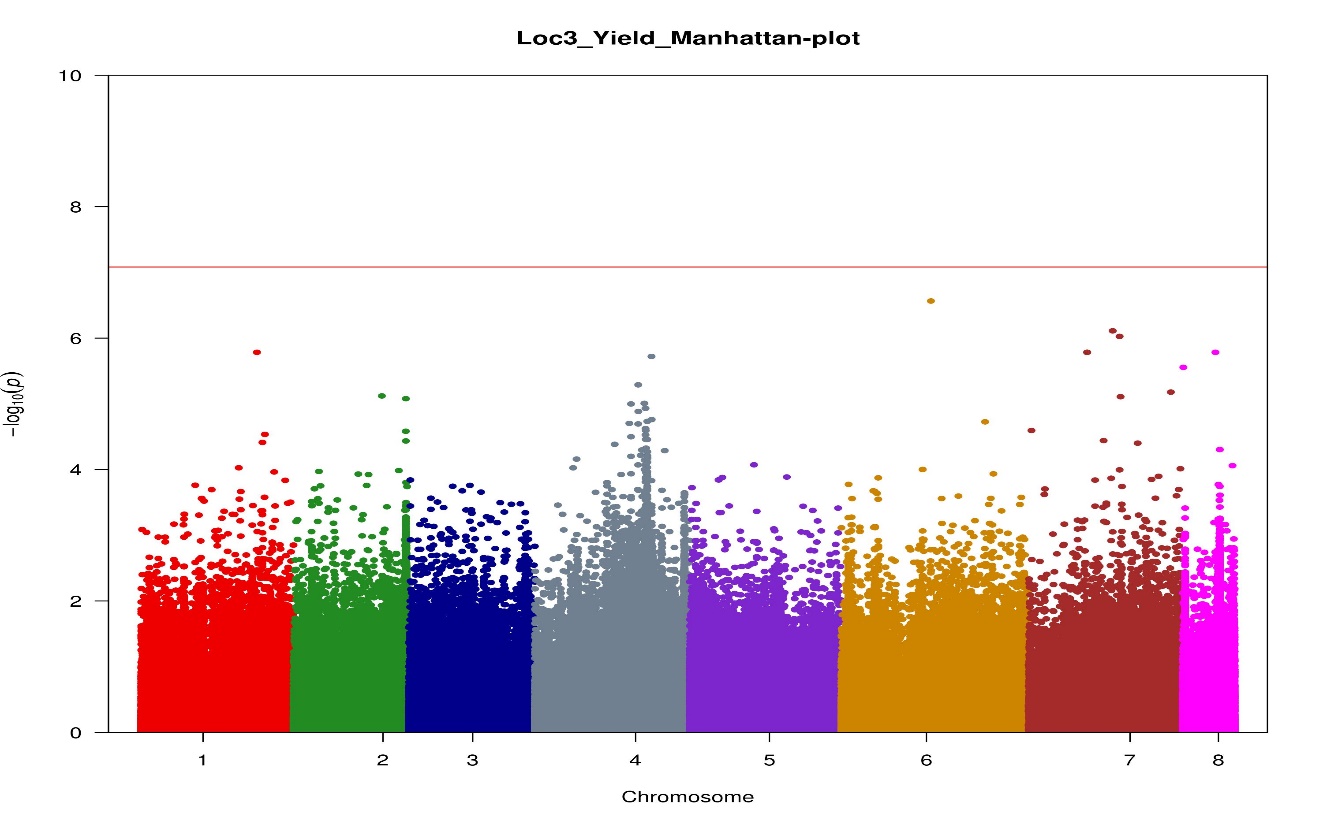


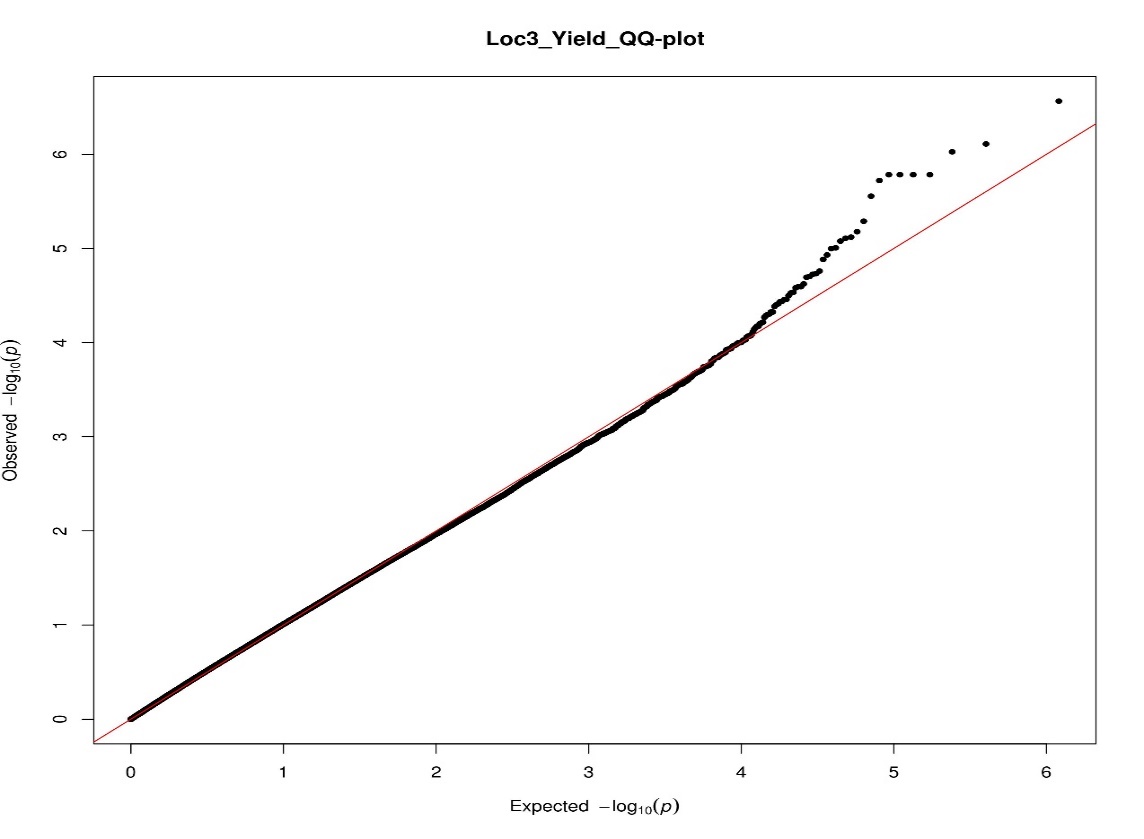


**Supplementary figure 5(f)**: Manhattan plot illustrating SNPs linked to yield with their corresponding statistical significance represented by Q-Q plot for location 3 -Blink model


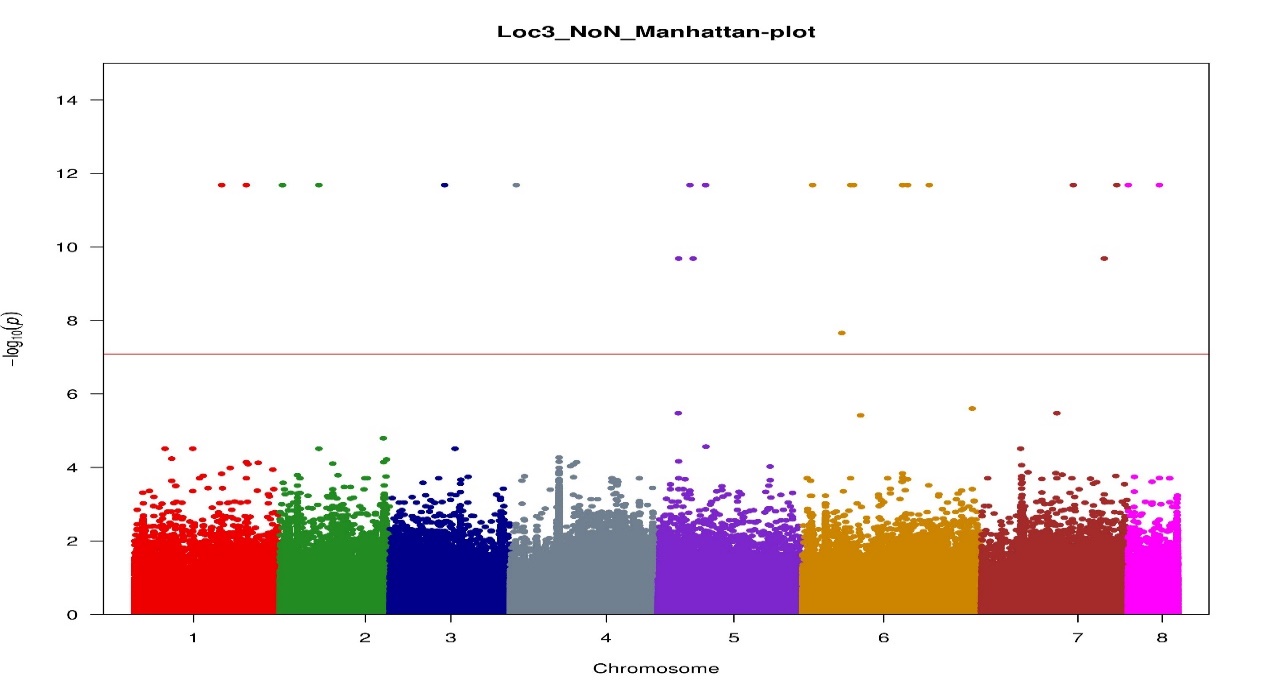


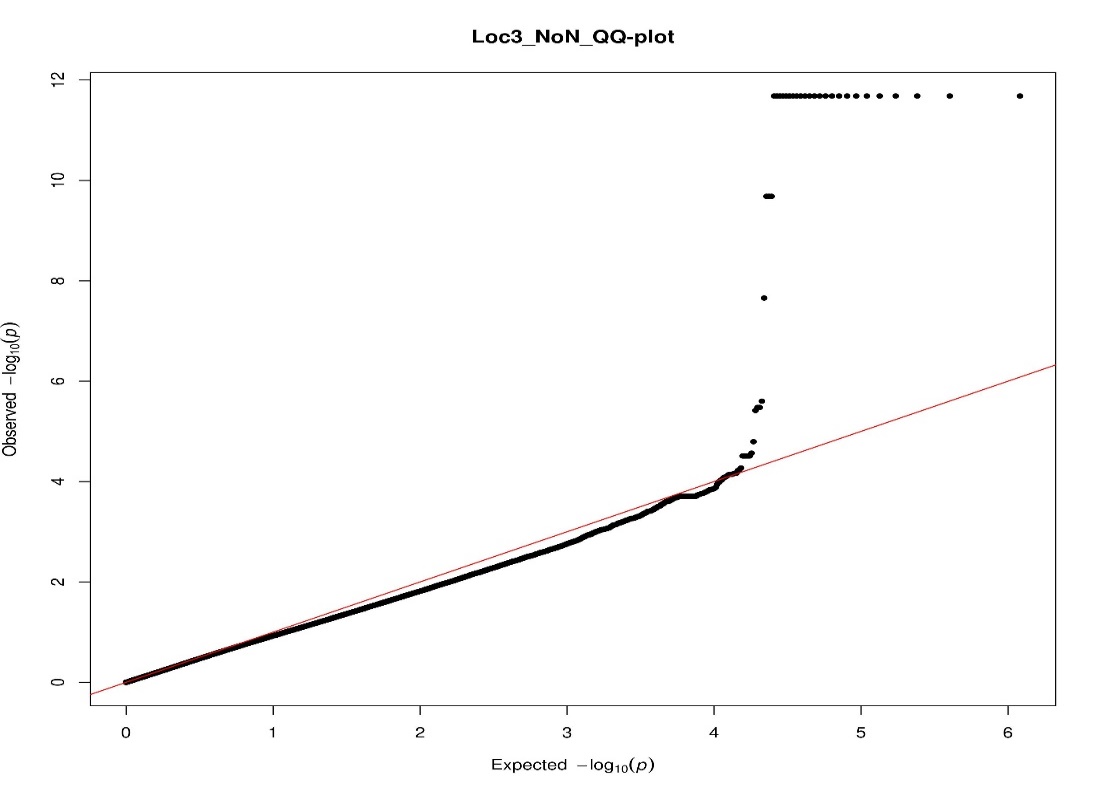


**Supplementary figure 6(a)**: Manhattan plot illustrating SNPs linked to number of nodules with their corresponding statistical significance represented by Q-Q plot for location 3 -FarmCPU model


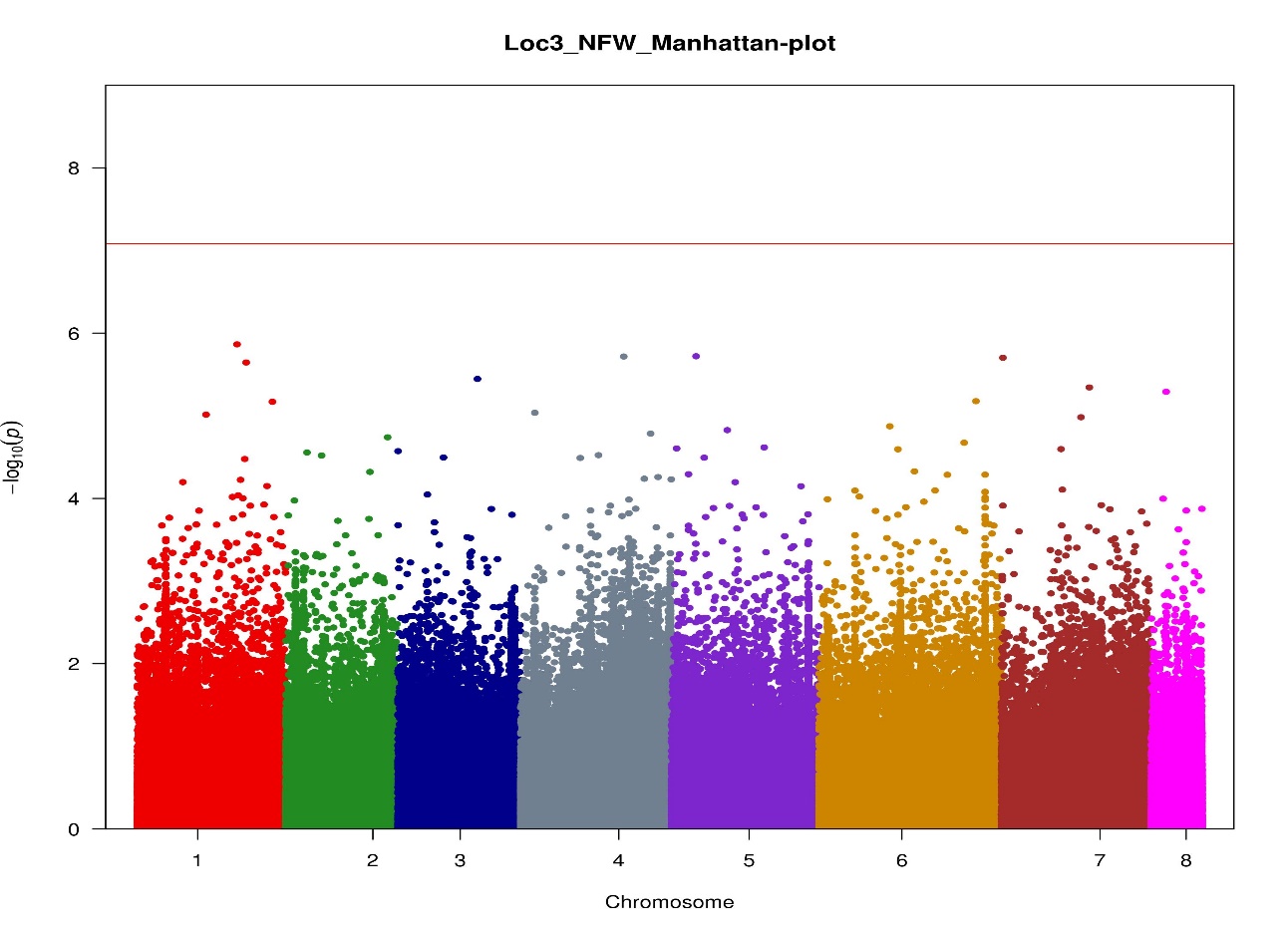


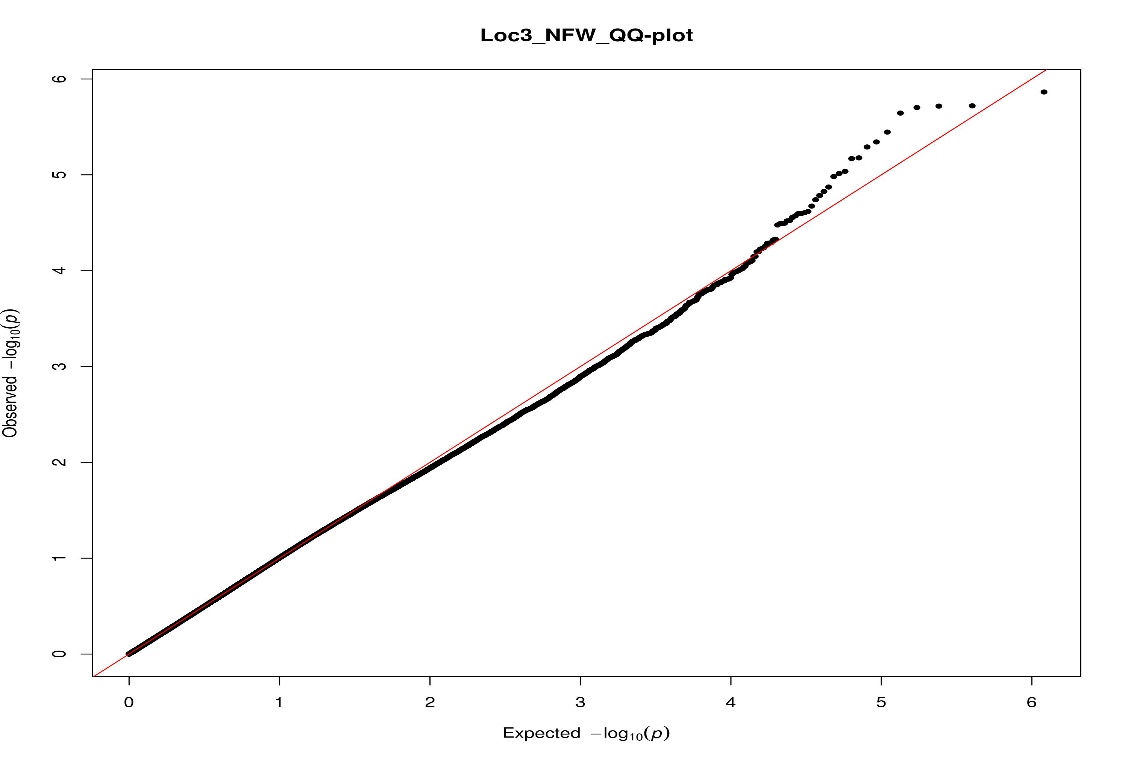


**Supplementary figure 6(b)**: Manhattan plot illustrating SNPs linked to nodule fresh weight with their corresponding statistical significance represented by Q-Q plot for location 3 -FarmCPU model


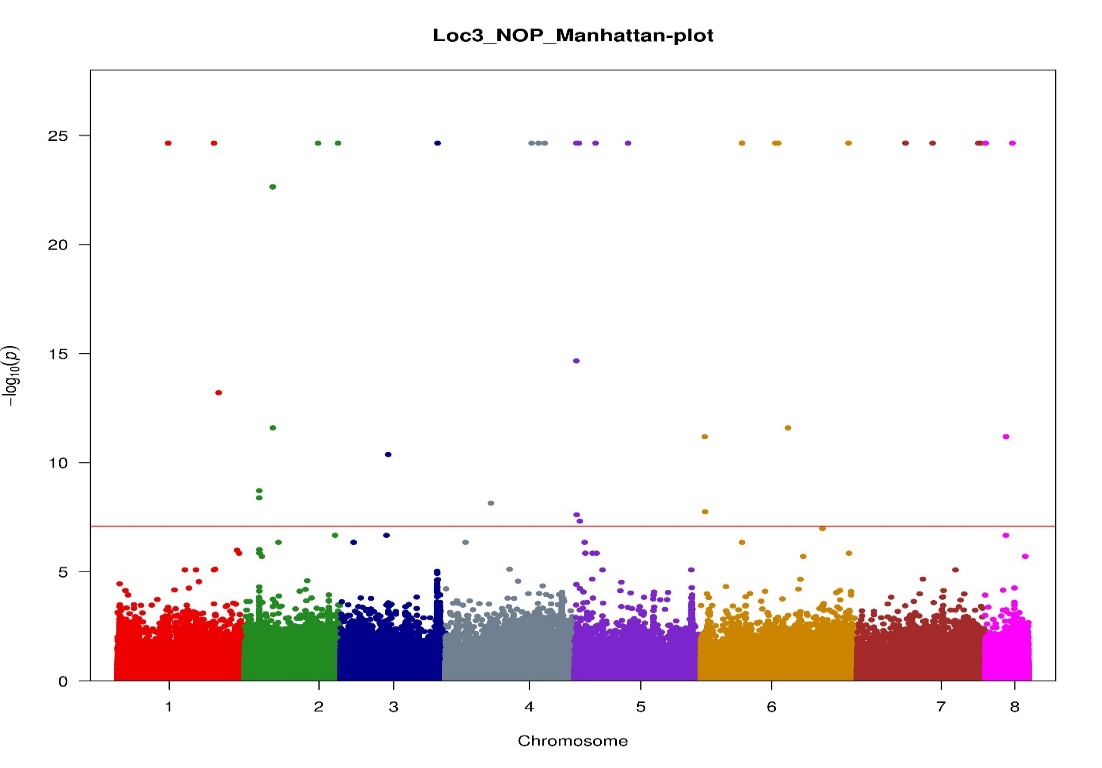


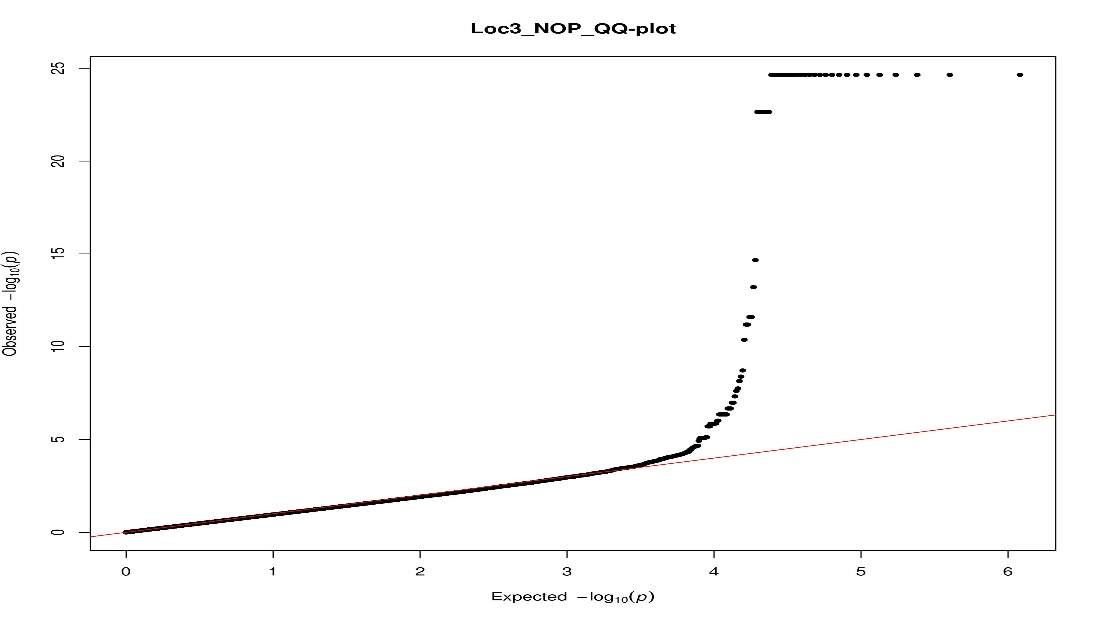


**Supplementary figure 6(c)**: Manhattan plot illustrating SNPs linked to number of pods with their corresponding statistical significance represented by Q-Q plot for location 3 -FarmCPU model


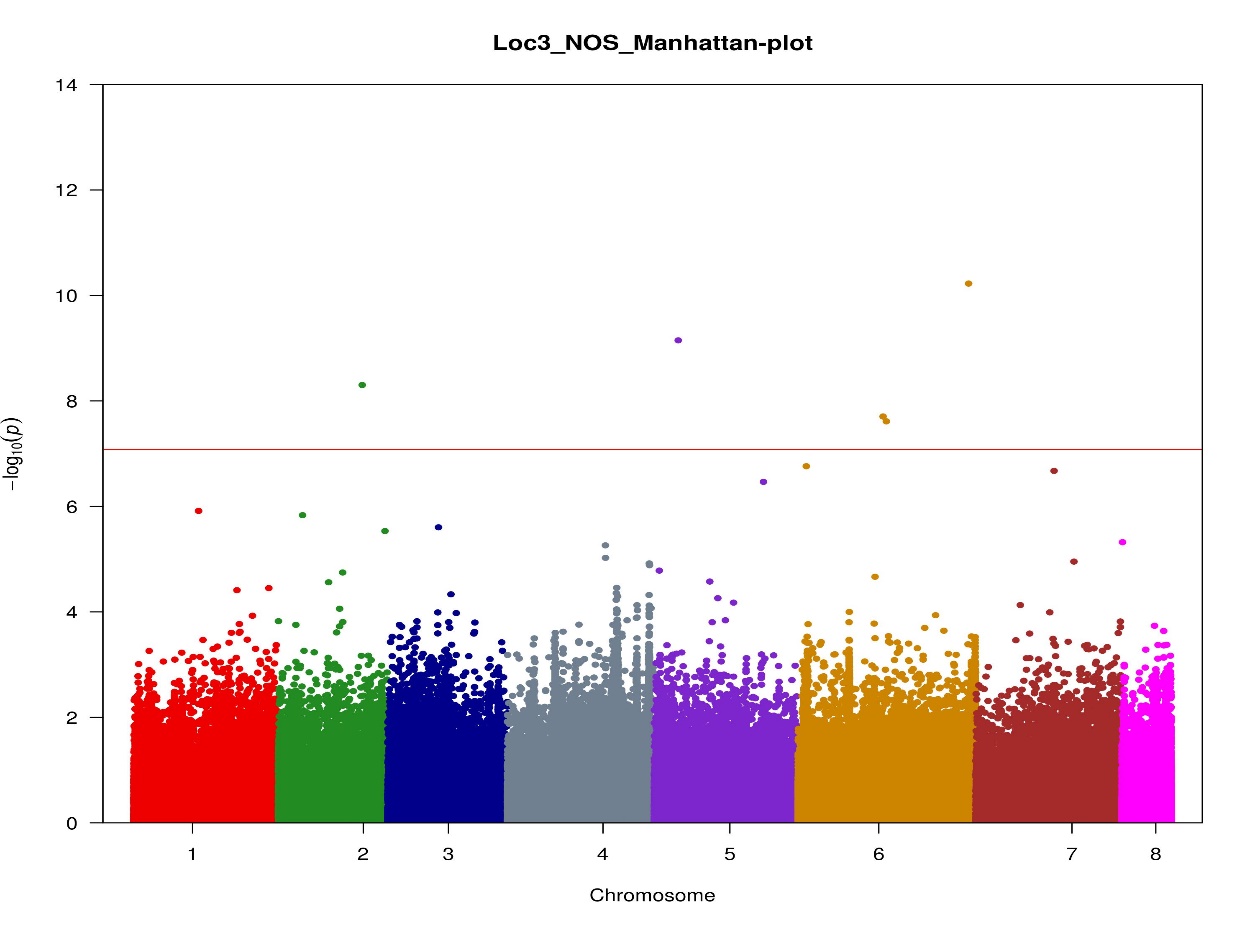


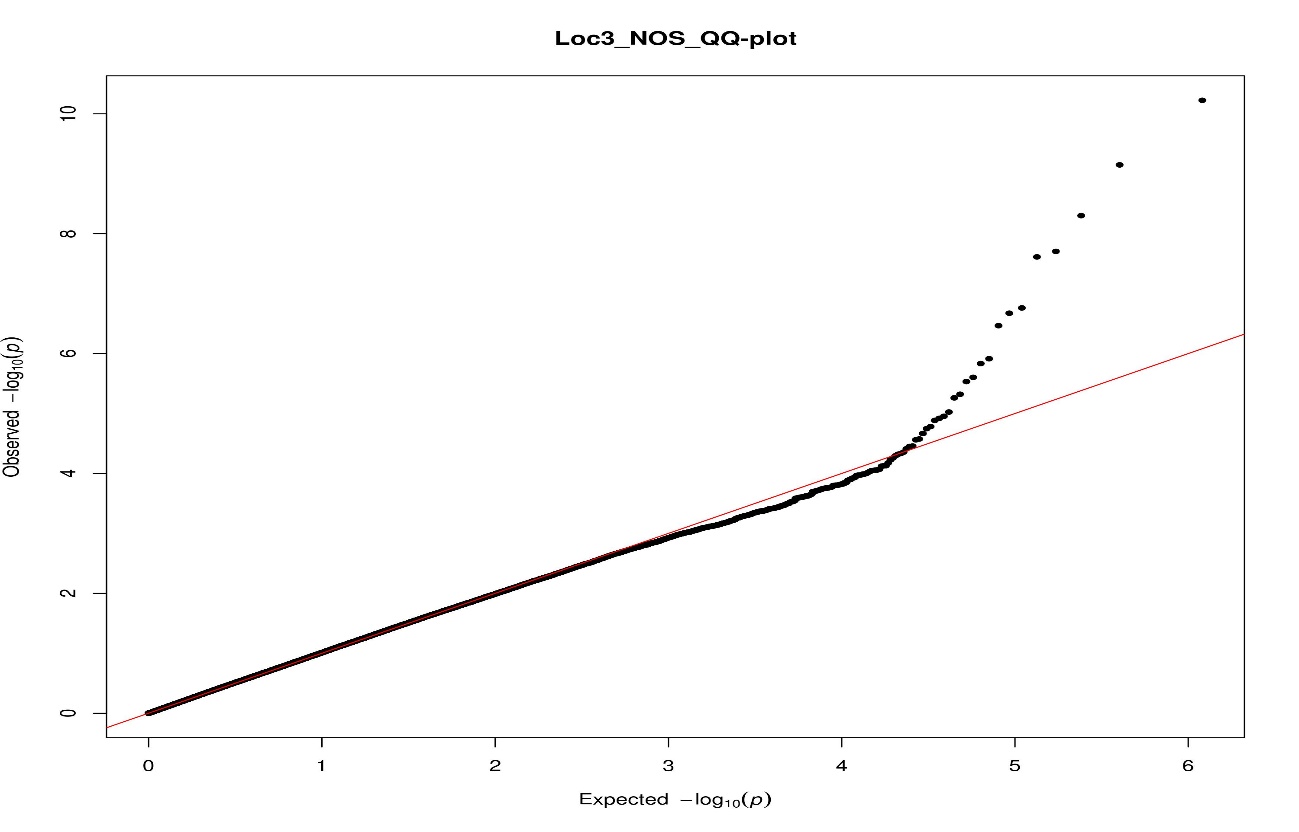


**Supplementary figure 6(d)**: Manhattan plot illustrating SNPs linked to number of seeds with their corresponding statistical significance represented by Q-Q plot for location 3 -FarmCPU model


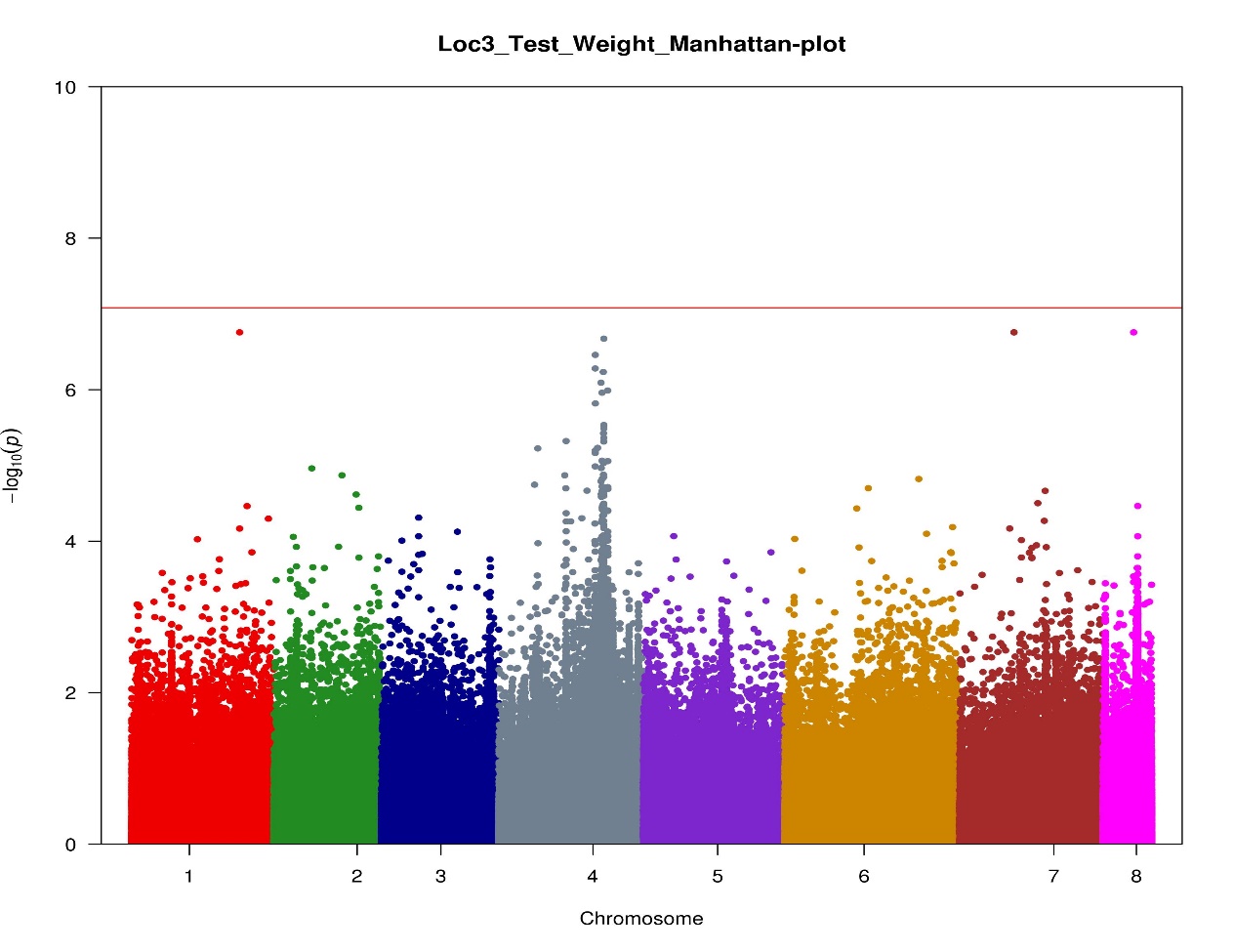


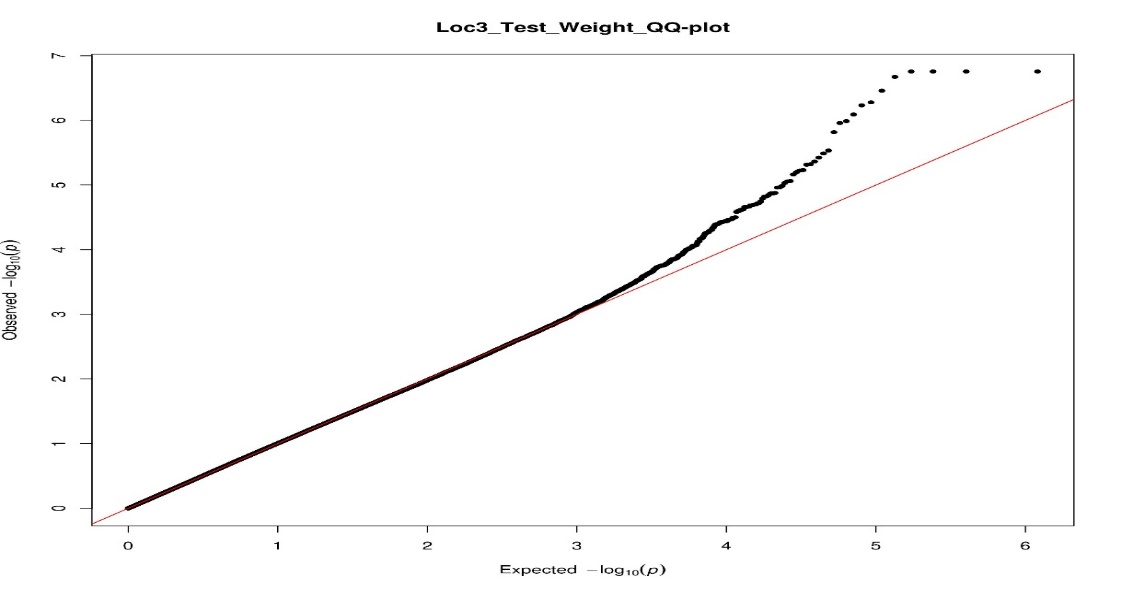


**Supplementary figure 6(e)**: Manhattan plot illustrating SNPs linked to test weight with their corresponding statistical significance represented by Q-Q plot for location 3 -FarmCPU model


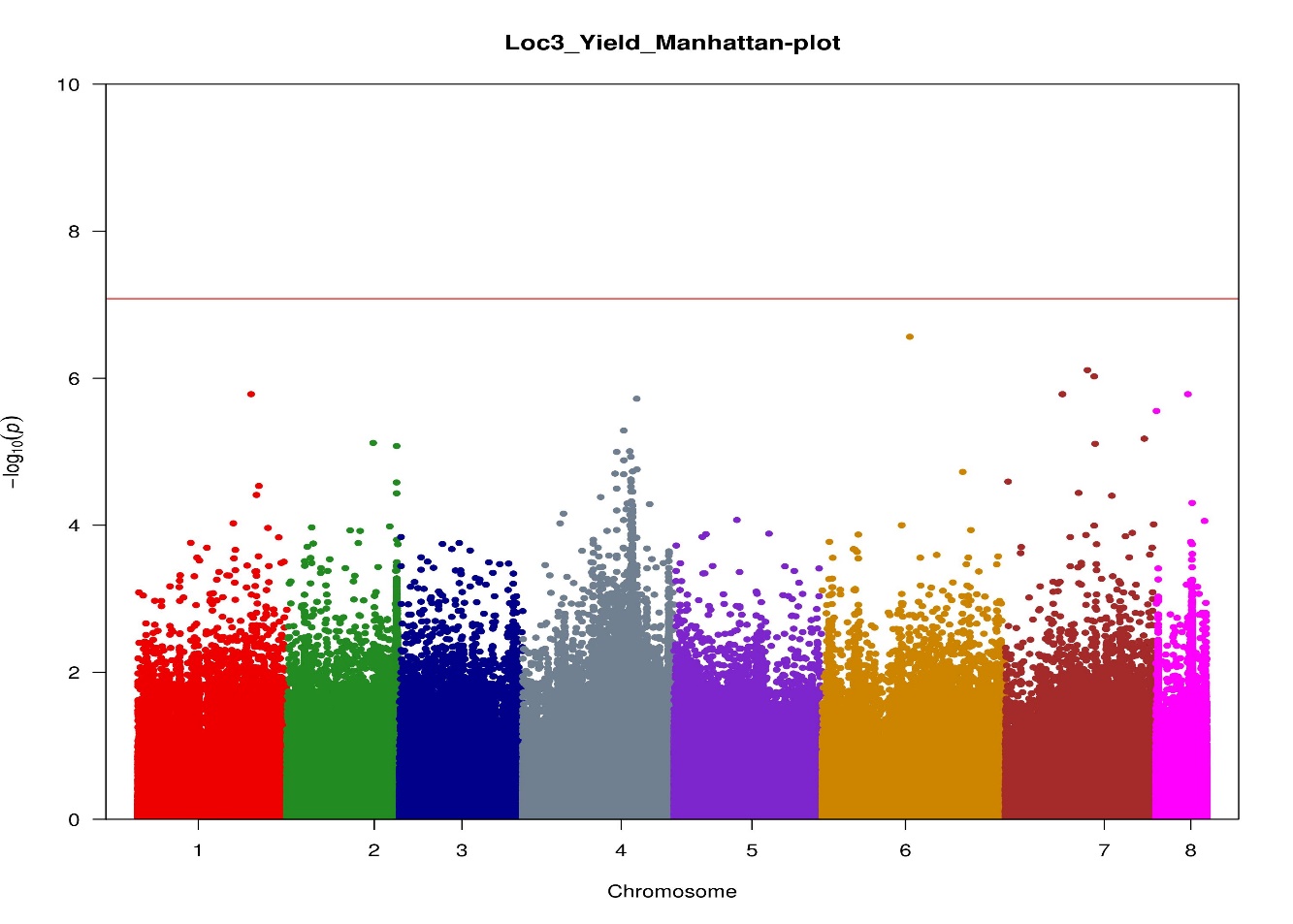


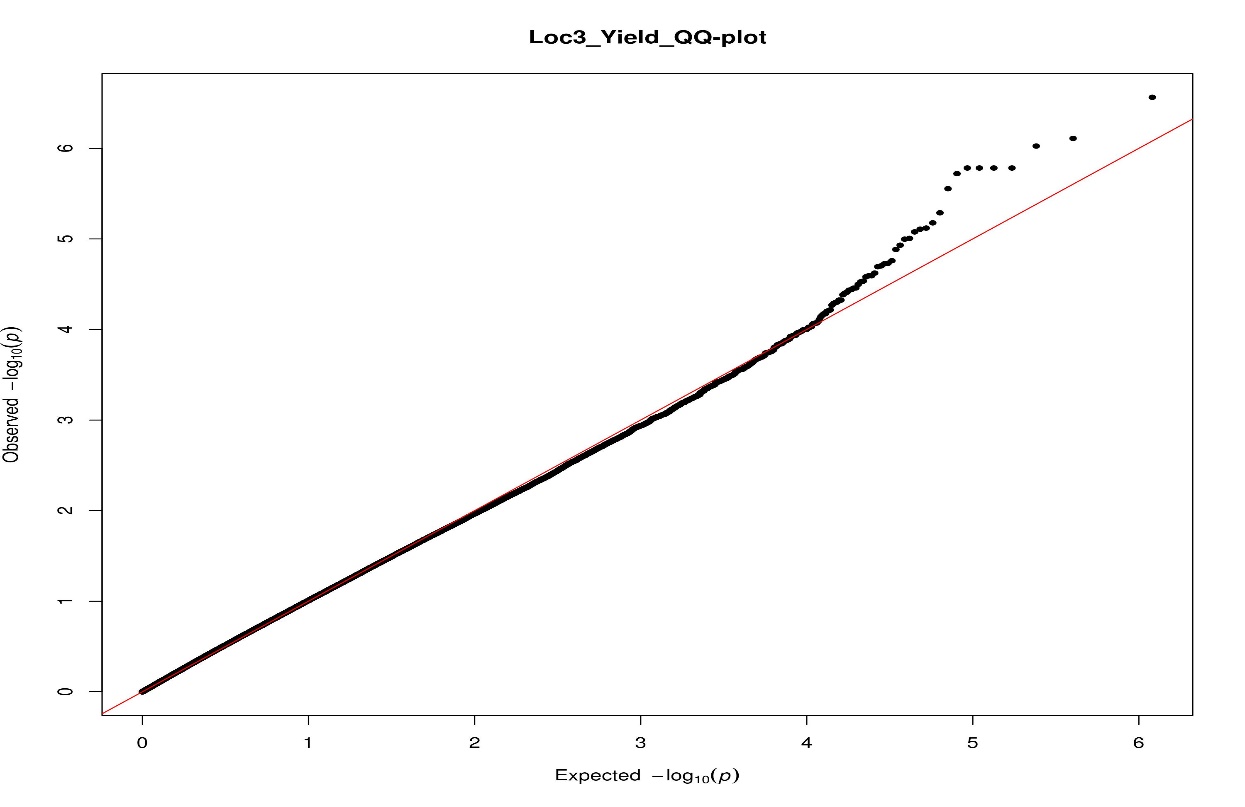


**Supplementary figure 6(f)**: Manhattan plot illustrating SNPs linked to yield with their corresponding statistical significance represented by Q-Q plot for location 3 -FarmCPU model
